# Multi-Dimensional Benchmarking of Liquid Chromatographic Methods for Parabens Determination in Cosmetic Products: Toward Sustainable Analysis

**DOI:** 10.3390/molecules31213828

**Published:** 2026-10-23

**Authors:** Abdulmalik M. Alqarni, Heba Shaaban, Ahmed Mostafa, Shmoukh A. Albugami, Noor Bunajmah, Turki Aldossary, Ghada Alsumain, Mohammed Alqarni, Abdulateef Alqahtani

**Affiliations:** 1 Department of Pharmaceutical Chemistry, College of Pharmacy, Imam Abdulrahman Bin Faisal University, King Faisal Road, P.O. Box 1982, Dammam 31441, Saudi Arabia; amalqarni@iau.edu.sa (A.M.A.); ammostafa@iau.edu.sa (A.M.); 2 College of Pharmacy, Imam Abdulrahman Bin Faisal University, Dammam 31441, Saudi Arabia; 2220007016@iau.edu.sa (S.A.A.); 2220004196@iau.edu.sa (N.B.); 2220002674@iau.edu.sa (T.A.); 2140008293@iau.edu.sa (G.A.); 3 Department of Pharmaceutical Chemistry, College of Pharmacy, Taif University, P.O. Box 11099, Taif 21944, Saudi Arabia; m.aalqarni@tu.edu.sa; 4 Department of Pharmaceutical Chemistry, Faculty of Pharmacy, Al-Baha University, P.O. Box 1988, Al-Baha 65431, Saudi Arabia; aalqhtani@bu.edu.sa

**Keywords:** green chemistry, sustainable analysis, assessment tools, IMOGI, green analysis, SDGs

## Abstract

Background/Objectives: Consumer safety is an important consideration in the use of cosmetic and personal care products, particularly because individuals may be exposed to multiple products concurrently, increasing potential exposure to preservatives such as parabens. Reliable analytical methods are therefore essential for monitoring parabens in cosmetic products. In line with the principles of Green Analytical Chemistry (GAC), analytical method development and selection should also consider environmental impact, resource consumption, waste generation, and occupational safety. This study aimed to benchmark liquid chromatographic methods for paraben determination in cosmetic products using a multi-metric approach, with emphasis on greenness, practicality, and innovation to support more sustainable analytical method selection. Methods: Sixteen chromatographic methods for the determination of parabens in cosmetic products were selected after screening the retrieved records and evaluated using complementary assessment tools, including the Modified Green Analytical Procedure Index (MoGAPI), Blue Applicability Grade Index (BAGI), and Innovative Method Orange Gradient Index (IMOGI), covering greenness, practical applicability, and analytical innovation, respectively. Results: The selected methods showed MoGAPI scores of 62–74, BAGI scores of 55–80, and IMOGI scores of 10–50, indicating substantial differences across the evaluated dimensions. The methods showed different profiles across greenness, practical applicability, and innovation, with favorable results in one dimension not necessarily corresponding to favorable results in the others. The multi-metric assessment revealed differences and potential trade-offs among these three dimensions that may remain unrecognized when methods are evaluated using a single assessment tool. Conclusions: The benchmarking highlighted the key driving factors influencing the greenness, practical applicability, and innovation profiles of the evaluated methods. The multi-metric assessment provides a broader basis for context-dependent analytical method selection and advancing more sustainable analytical practices, promoting responsible resource use and alignment with the United Nations Sustainable Development Goals (SDGs).

## 1. Introduction

Cosmetic and personal care products are widely used in daily life and contain a diverse range of chemical substances, some of which may raise concerns regarding human health. These include preservatives such as parabens, plasticizers such as phthalates, and potentially toxic elements such as heavy metals [1]. Parabens constitute an important group of antimicrobial preservatives that are extensively employed in cosmetic and personal care formulations. Their effectiveness against microorganisms, chemical stability, and compatibility with different formulation components have supported their widespread use in cosmetics and personal care products [2]. The extensive use of parabens has attracted increasing attention because of concerns regarding their possible effects on human health [3]. These compounds have been investigated as endocrine-disrupting chemicals because of their potential interactions with endocrine-related biological pathways [4]. Various biological effects have been reported in the literature, including potential influences on reproductive processes, adipogenesis, skin responses, and other physiological functions [5]. Continuous or repeated exposure associated with the use of personal care products has consequently become an important research concern [1,6]. Reliable analytical determination of parabens in cosmetic products is therefore important not only for quality control purposes but also for supporting the evaluation of potential consumer exposure [7].

Accurate determination of parabens in cosmetic matrices is not always straightforward because these products encompass diverse and chemically complex formulations. The presence of multiple ingredients and the occurrence of parabens at different concentration levels necessitate appropriate separation and sample-preparation procedures [8]. Among the available analytical techniques, high-performance liquid chromatography (HPLC) has been extensively employed because it provides effective separation and quantitative determination of several paraben compounds [9].

The development of paraben analysis has been accompanied by substantial progress in both sample preparation and chromatographic methodologies. Traditional liquid solvent extraction has progressively been supplemented or replaced by approaches such as solid-phase extraction (SPE), magnetic solid-phase extraction, molecularly imprinted polymers (MIPs), microextraction by packed sorbent (MEPS), dispersive liquid–liquid microextraction (DLLME), and other miniaturized extraction procedures [10]. More recent developments have introduced deep eutectic solvents (DES), natural deep eutectic solvents (NADES), ionic liquids, magnetic materials, and other engineered sorbents as alternative extraction media [11]. These developments have expanded the technological options available for paraben analysis, but they have also introduced considerable variation in solvent requirements, material use, waste generation, sample-preparation complexity, and resource consumption. Nevertheless, methodological advancement does not necessarily imply a reduced environmental burden. Some analytical procedures continue to require considerable amounts of organic solvents, generate significant chemical waste, or involve relatively complex sample-preparation steps [11]. These issues have increased the importance of incorporating environmental considerations into analytical method development [12]. Strategies such as replacing conventional solvents with more environmentally preferable alternatives, reducing solvent volumes, minimizing waste, and shortening analytical procedures can contribute to improving the environmental profile of analytical workflows [13]. Recent applications of DES/NADES-based extraction for paraben determination exemplify efforts to reduce the environmental impact associated with conventional chromatographic analysis [14]. However, the introduction of an alternative solvent, sorbent, or miniaturized extraction strategy alone does not necessarily guarantee an environmentally sustainable analytical procedure. A comprehensive evaluation of the entire workflow is therefore required to identify the factors that contribute to, or compromise, its environmental performance.

Within this context, Green Analytical Chemistry (GAC) has emerged as an important approach for encouraging the development of analytical procedures with reduced environmental impacts [15]. GAC principles emphasize, among other aspects, the reduction in hazardous substances, waste generation, energy consumption, and resource use throughout the analytical process [16]. Several assessment approaches have consequently been introduced to characterize the environmental performance of analytical procedures, including Analytical GREEnness (AGREE) [17] and Modified Green Analytical Procedure Index (MoGAPI) [18]. These tools provide complementary means of examining the environmental characteristics of analytical workflows and can help identify aspects requiring improvement. Such assessment is particularly relevant when comparing published analytical methods, as differences in extraction media, solvent consumption, waste generation, and other workflow characteristics may not be apparent from conventional analytical performance parameters alone.

The ease with which a procedure can be implemented in a routine laboratory is equally important, particularly for cosmetic quality-control applications. Practical considerations may include the amount of sample required, duration and complexity of sample preparation, total analysis time, equipment and material requirements, cost, accessibility of reagents, and the level of technical expertise needed [18]. A method that provides satisfactory analytical results but requires specialized materials, extensive preparation, or substantial resources may have limited applicability in routine settings. The Blue Applicability Grade Index (BAGI) was introduced to provide a structured means of evaluating these practical aspects of analytical procedures [19]. From a sustainability perspective, consideration of practical applicability can help distinguish approaches that are environmentally favorable in principle from those that can realistically be implemented and maintained in routine analytical laboratories.

The rapid evolution of analytical technologies has made innovation and modernization an increasingly relevant dimension in method evaluation. Advances may arise from different parts of the analytical workflow, including novel sample-preparation strategies, engineered sorbents, miniaturized extraction systems, modern chromatographic configurations, advanced detection technologies, automation, experimental design, and digital approaches [20]. The Innovative Method Orange Gradient Index (IMOGI) offers a dedicated framework for characterizing the extent to which such innovative and modern features are incorporated into analytical methods [20]. Importantly, technological innovation can support sustainability when it enables miniaturization, solvent reduction, waste minimization, rapid analysis, automation, or more efficient use of analytical resources. Thus, innovation and modernization may provide mechanisms through which sustainability objectives can be incorporated into analytical method design.

Consequently, assessment based solely on conventional analytical characteristics such as sensitivity, accuracy, precision, or linearity may not provide a complete picture of sustainability and broader methodological characteristics of a chromatographic method. Two procedures with comparable analytical performance may differ substantially in their environmental impact, operational requirements, or technological advancement. Similarly, the introduction of an innovative material or analytical configuration does not necessarily ensure favorable environmental or practical characteristics [21]. A multidimensional benchmarking approach can therefore provide a more informative basis for examining existing methods, identifying the sources of environmental burden, and recognizing practical or technological features that may facilitate the transition toward more sustainable analytical workflows.

In this study, a multidimensional benchmarking evaluation was therefore undertaken for liquid chromatographic methods reported for the determination of parabens in cosmetic products using MoGAPI, BAGI, and IMOGI, respectively. The assessment aimed to characterize the methodological diversity of existing approaches, identify their principal environmental, practical, and technological strengths and limitations, and determine opportunities for advancing paraben analysis toward more sustainable liquid chromatographic workflows through greener extraction strategies, resource-efficient sample preparation, practical implementation, and appropriate technological innovation.

## 2. Results and Discussion

### 2.1. Overview of the Selected Chromatographic Methods

Sixteen chromatographic methods for the determination of parabens in cosmetic products were selected for comparative assessment. The methods represent a range of chromatographic platforms and sample-preparation strategies, from conventional solvent extraction to more recent approaches incorporating microextraction, deep eutectic solvents (DESs), magnetic and molecularly imprinted sorbents. Several studies also introduced advances in chromatographic separation. The selected methods differed considerably in their analytical characteristics, including cosmetic matrix, target analytes, sample amount, sample-preparation procedure, and chromatographic conditions as summarized in Table 1. Additional analytical performance parameters, including the limits of quantification (LOQs), recoveries, precision, and linear ranges, are provided in Supplementary Table S1. This methodological diversity provides a suitable basis for examining paraben analysis from complementary sustainability-oriented perspectives. Accordingly, the selected methods were evaluated in terms of environmental sustainability, practical applicability, and analytical innovation using MoGAPI, BAGI, and IMOGI, respectively. The results of these three assessments are discussed in the following sections.

### 2.2. Environmental Sustainability Assessment Using MoGAPI

**Figure 1:**
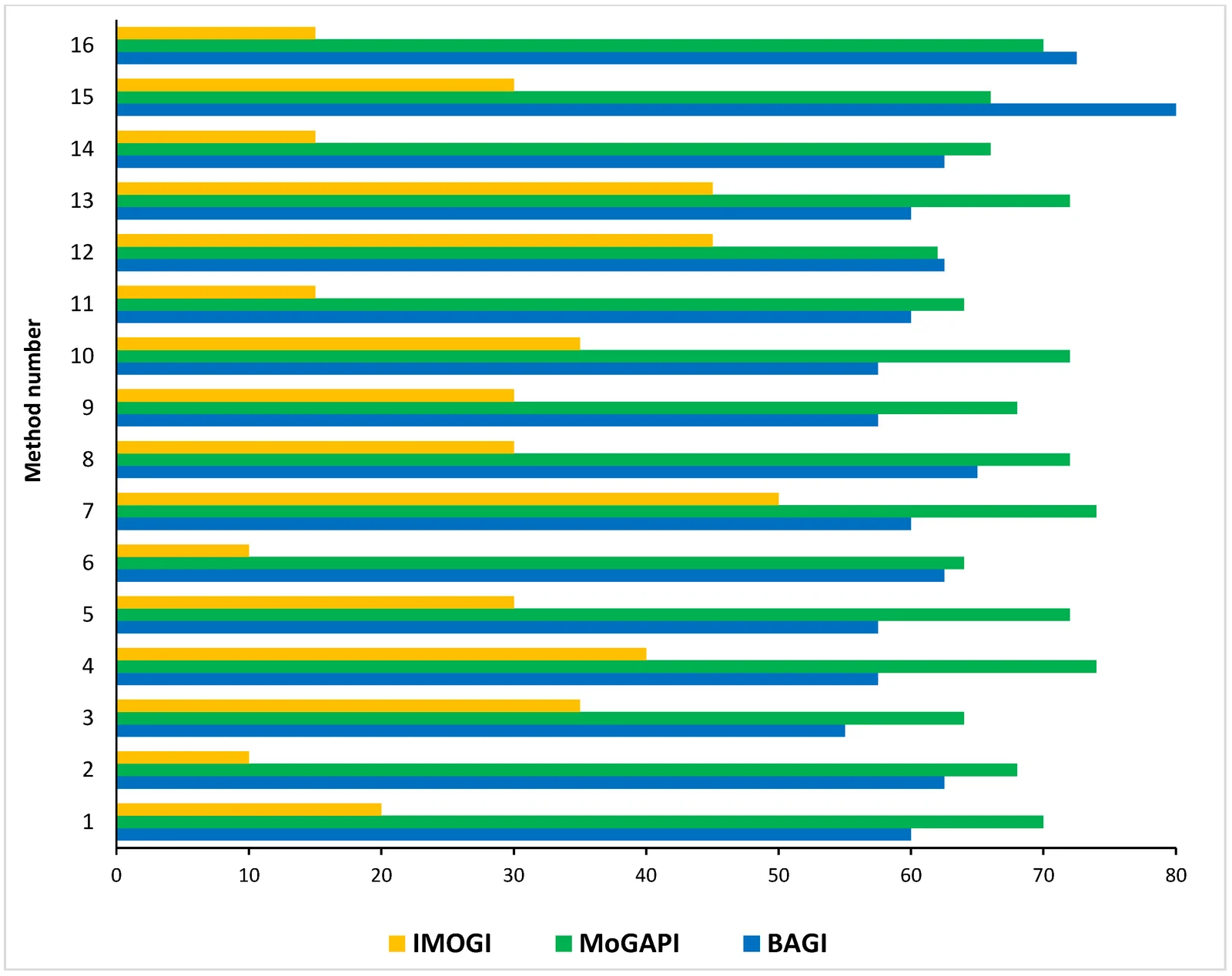
Distribution of MoGAPI, BAGI, and IMOGI scores across the selected chromatographic methods for paraben determination in cosmetic products.

The MoGAPI assessment showed that most of the selected methods exhibited relatively favorable environmental profiles, with scores ranging from 62 to 74 as indicated in Table 2 and Figure 1.

Methods 4 and 7 achieved the highest scores (74), followed by Methods 5, 8, 10, and 13 (72). The favorable MoGAPI performance was mainly associated with reduced solvent consumption, the use of alternative extraction media, and reduced waste generation. Methods 4 and 7, employing DES- and NADES-based extraction, respectively, achieved the highest scores, reflecting the use of alternative extraction media with a lower environmental burden than conventional organic solvents. Methods 5, 10, and 13 also achieved high scores (72), with relatively low extraction-solvent volumes (<10 mL) contributing to lower solvent consumption and waste generation within the analytical workflow. Method 5 further incorporated ionic-liquid-assisted LLME combined with magnetic solid-phase extraction, while Method 10 used DES-based microextraction and Method 13 employed MEPS. Method 8 also achieved a high score (72), owing to the use of ethanol as the extraction solvent, a relatively simple sample-preparation procedure, and a low solvent volume (<10 mL). The criterion-by-criterion assessment for the MoGAPI tool is provided in Table S2.

It is also noteworthy that the highly sustainable methods M4 and M7 (MoGAPI score: 74) demonstrated that favorable environmental performance can be achieved alongside satisfactory analytical performance in paraben determination in cosmetic products. Their reported LODs, LOQs, coefficients of determination (R^2^), and recoveries indicated satisfactory analytical performance. These findings suggest that environmental sustainability can potentially be integrated into routine cosmetic analysis without necessarily compromising analytical performance. Overall, these findings indicate that solvent selection, solvent consumption, alternative extraction media, and waste generation were important factors contributing to the environmental profiles of the investigated methods. The results further demonstrate that environmentally preferable solvent systems and solvent minimization can contribute to reducing the environmental burden of chromatographic methods for paraben determination.

### 2.3. Practical Applicability Assessment Using BAGI

The BAGI assessment revealed greater variability in the practical applicability of the selected chromatographic methods compared with their environmental sustainability. BAGI scores ranged from 55 to 80, indicating notable differences in the practical requirements and operational feasibility of the investigated methods (Figure 1).

Method 15 achieved the highest BAGI score (80), followed by Method 16 (72.5). The high applicability of Method 15 can be attributed to its very small sample requirement (40 µL), very short analysis time (90 s), relatively simple sample preparation, and the use of commercially available solvents and reagents. These characteristics facilitate straightforward implementation and are particularly advantageous for routine and high-throughput analysis. Method 16 also showed high practical applicability (72.5), supported by its small sample amount, rapid chromatographic separation using a core-shell column, and the use of commercially available solvents and reagents, which facilitate implementation without requiring specialized chemical materials.

In contrast, Method 3 exhibited the lowest BAGI score (55). Its lower practical applicability can be associated with the relatively demanding sample-preparation procedure based on a molecularly imprinted polymer (MIP) and the use of several reagents, together with a comparatively larger sample amount (1 g). Although MIPs can provide enhanced selectivity, their use may reduce practical accessibility because such specialized materials are not routinely available or readily accessible in all analytical laboratories, and their preparation can introduce additional operational requirements. Methods 4, 5, 9, 10, and 11 showed intermediate BAGI scores (57.5–60), reflecting differences in sample preparation and other operational requirements. The individual BAGI scoring criteria and their application to the investigated methods are presented in Table S3. Overall, the BAGI assessment indicates that sample amount, analysis time, simplicity of sample preparation, accessibility of materials, availability of solvents and reagents, and operational requirements are important factors influencing the practical applicability of chromatographic methods for paraben determination. Methods requiring readily accessible materials, smaller sample quantities, and simpler and faster procedures are generally more amenable to routine laboratory implementation.

### 2.4. Innovation and Modernization Assessment Using IMOGI

The IMOGI assessment showed scores ranging from 10 to 50 (Figure 1), indicating considerable differences in the degree of innovation and modernization incorporated into the investigated chromatographic methods.

Method 7 achieved the highest IMOGI score (50), followed by methods 12 and 13 (45). The high score of Method 7 reflects the combination of NADES-based dispersive liquid–liquid microextraction (NADES-DLLME), miniaturized sample preparation, and UHPLC-DAD, representing advances in sample preparation and chromatographic analysis. Method 12 also demonstrated a high degree of innovation through the use of an advanced nanostructured sorbent and dispersive micro-solid-phase extraction, illustrating the incorporation of emerging materials and miniaturized sample preparation into paraben analysis. Method 13 combined microextraction by packed sorbent (MEPS) with systematic experimental design for method optimization, while Method 4 introduced a deep eutectic solvent-based microextraction strategy, contributing to its relatively high innovation score.

Innovation was also evident in methods that incorporated modern chromatographic separation or detection strategies. For example, Method 15 employed programmable low-pressure chromatography with a monolithic C18 column and post-column chemiluminescence detection, enabling an exceptionally rapid analysis of less than 2 min. Method 16 used a core-shell column to achieve rapid chromatographic separation, demonstrating modernization through improved chromatographic efficiency. These examples illustrate that analytical innovation may arise from different components of the workflow, including sample preparation, sorbent design, chromatographic separation, detection, miniaturization, and method optimization.

In addition to technological innovations, several methods demonstrated a more explicit incorporation of GAC principles, particularly through the adoption of alternative solvents, solvent minimization, waste reduction, and miniaturization. The favorable environmental performance of these approaches, as indicated by recognized assessment tools, provides evidence that sustainability considerations can be integrated into the development of modern analytical methods. In this context, innovation should not be restricted to the introduction of new materials or instrumentation but may also involve redesigning established analytical workflows according to contemporary GAC principles. Such integration is particularly relevant to sustainable analytical development, where technological advances can be directed toward reducing resource consumption, solvent use, waste generation, and analytical time.

At the lower end, methods 2 and 6 showed the lowest IMOGI scores (10), while methods 11, 14, and 16 received relatively low scores of 15. These scores reflect the extent of innovation and modernization captured by the IMOGI criteria rather than the overall usefulness or analytical quality of the methods. These methods relied predominantly on established chromatographic and sample-preparation configurations, resulting in lower scores according to the IMOGI criteria. Importantly, a lower IMOGI score does not indicate poor analytical performance; rather, it reflects the extent of methodological innovation and modernization captured by the assessment criteria. The criterion-by-criterion assessment for the IMOGI tool is provided in Table S4 of the Supporting Information.

Overall, the IMOGI assessment demonstrates that innovation in paraben analysis is multidimensional and can be achieved through novel extraction approaches, advanced sorbent materials, miniaturization, modern chromatographic technologies, rapid separation, alternative detection strategies, and integration of GAC principles. From the perspective of sustainable analytical development, these innovations are particularly valuable when they simultaneously reduce resource requirements or environmental burden while improving workflow efficiency. The results also highlight the value of assessing innovation independently, since methodological novelty and modernization cannot be fully represented by environmental sustainability or practical applicability alone.

### 2.5. Multidimensional Assessment and Future Perspectives

The three-dimensional assessment demonstrated that environmental sustainability, practical applicability, and analytical innovation are complementary characteristics of chromatographic methods for paraben determination. The investigated methods exhibited distinct profiles, indicating that improvement in one dimension does not necessarily result in comparable performance in the others. Therefore, method selection should extend beyond a single assessment criterion and consider the overall balance among sustainability, practicality, and innovation in relation to the intended analytical application. This multidimensional perspective provides a more comprehensive understanding of the strengths and limitations of existing methods and can help identify approaches with greater potential for further development.

Several methods showed particularly promising and relatively balanced profiles. Method 7, for example, combined a high environmental sustainability score (MoGAPI = 74) with the highest innovation score (IMOGI = 50) and a moderate level of practical applicability (BAGI = 60). Its integration of NADES-based microextraction, miniaturized sample preparation, and UHPLC-DAD illustrates how environmental considerations and technological advancement can be incorporated within the same analytical workflow. Method 13 similarly demonstrated a favorable combination of environmental sustainability (MoGAPI = 72), practical applicability (BAGI = 60), and innovation (IMOGI = 45). Method 4 also exhibited a strong sustainability–innovation profile (MoGAPI = 74; IMOGI = 40), although its practical applicability was comparatively lower. These methods therefore provide useful examples of analytical approaches that move toward a more balanced design rather than optimizing a single characteristic. Other methods highlight specific directions for improvement. Method 15 achieved the highest practical applicability (BAGI = 80) through its very low sample requirement, short analysis time, and accessible analytical workflow, while Method 12 demonstrated substantial technological innovation (IMOGI = 45) through the use of advanced nanostructured sorbents and dispersive micro-solid-phase extraction. However, their profiles also indicate that further development could focus on improving the dimensions in which they are comparatively less favorable. Such observations demonstrate the value of a multidimensional assessment not only for comparing existing methods but also for identifying specific opportunities for method refinement.

To further investigate the relationships among environmental greenness, practical applicability, and methodological innovation, exploratory pairwise Spearman rank correlation analysis was performed across the 16 chromatographic methods. No statistically significant associations were found among MoGAPI, BAGI, and IMOGI (Table 3). These findings suggest that the three assessment tools capture distinct, potentially complementary dimensions of analytical methodology that do not necessarily vary in parallel. However, the observed correlations should be interpreted as exploratory patterns given the limited sample size (n = 16). Accordingly, considering these dimensions separately provides a more comprehensive characterization of analytical methods than relying on any single assessment tool. Further studies involving larger and more diverse sets of analytical methods are warranted to determine whether these patterns persist across different analytical applications.

The selection of an appropriate analytical method depends on its intended application, as the relative importance of environmental sustainability, practicality, and innovation may vary across analytical contexts. For high-throughput screening, analytical speed, sample-processing capacity, and operational simplicity may be particularly important. In regulatory quality control, validated analytical performance, reliability, reproducibility, and compliance with applicable requirements are essential, alongside sustainability considerations. In research-oriented method development, innovative extraction strategies, methodological advances, and opportunities to improve analytical performance may receive greater emphasis. Therefore, no single method can be considered universally preferable based solely on its performance in one assessment dimension. Instead, MoGAPI, BAGI, and IMOGI provide complementary perspectives that support context-dependent method selection according to the intended analytical purpose and relevant performance requirements.

From a future-development perspective, the findings suggest that the next generation of chromatographic methods for paraben determination should move beyond isolated improvements in extraction, separation, or detection and instead pursue integrated and sustainability-oriented workflow redesign. Advanced and miniaturized sample-preparation techniques, including microextraction, novel sorbents, and alternative solvent systems, can be further developed to reduce sample and solvent requirements while maintaining analytical performance. At the same time, digitalization and artificial intelligence (AI) could be incorporated into appropriate stages of the analytical workflow, including experimental design and optimization, chromatographic data processing, method selection, and prediction of analytical performance. These approaches may improve efficiency and reduce experimental burden and resource consumption, depending on their implementation and computational requirements.

**Figure 2:**
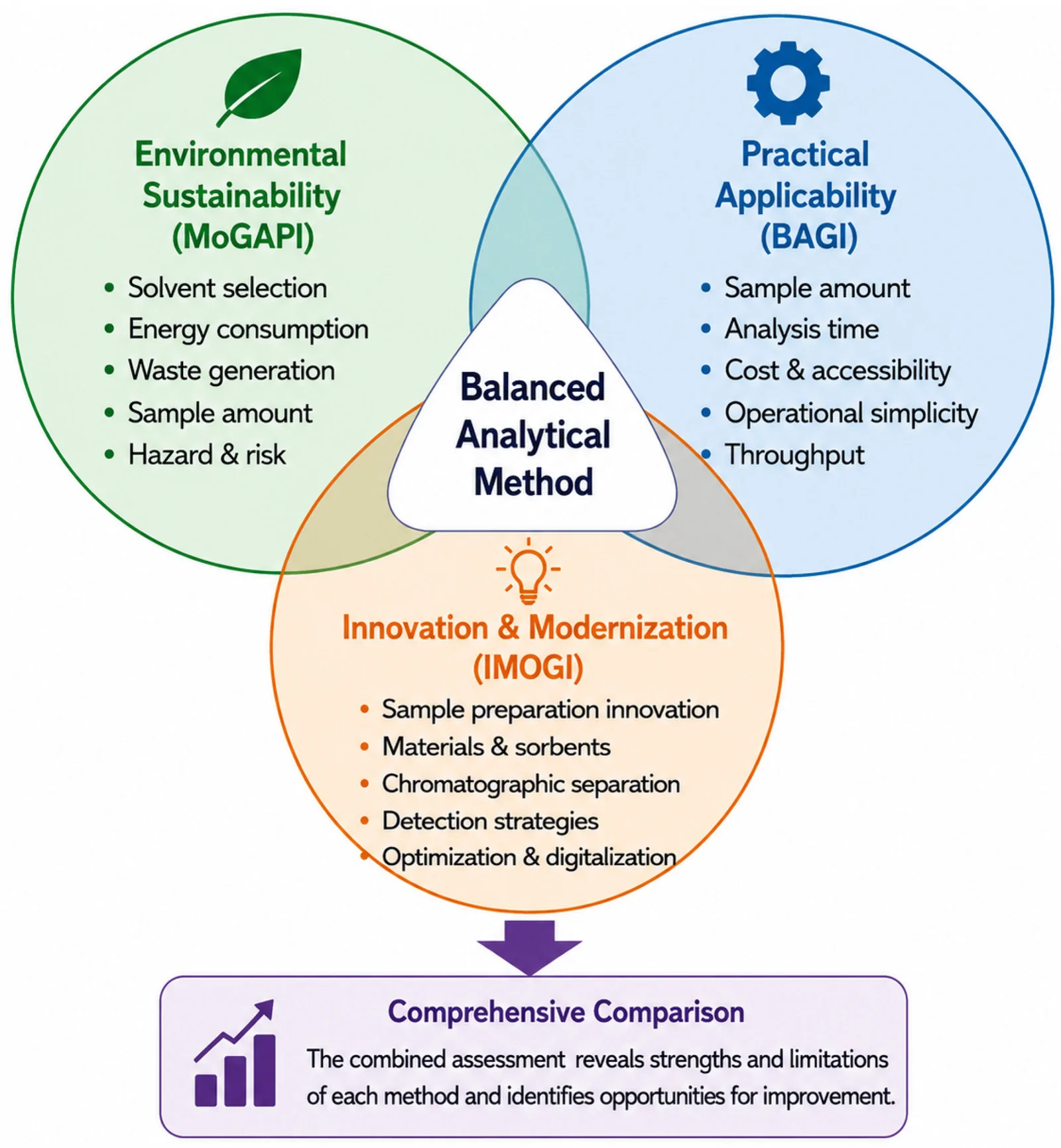
Schematic overview of the multidimensional approach for developing next-generation chromatographic methods for paraben determination in cosmetic products, integrating environmental sustainability, practical applicability, and innovation.

Importantly, future innovation should be guided by GAC principles from the initial method-design stage, rather than evaluating greenness only after the method has been developed. This requires reconsidering the complete analytical workflow, including sample collection and preparation, solvent and reagent selection, sample and solvent volumes, waste generation, chromatographic conditions, energy consumption, and opportunities for miniaturization and automation. The integration of these considerations with advanced technologies could enable the development of methods that are simultaneously more sustainable, practical, and innovative. Figure 2 summarizes the proposed direction toward next-generation paraben analysis, in which environmental sustainability, practical applicability, and innovation are considered simultaneously.

Future research should therefore prioritize the integration of environmentally preferable extraction media, resource-efficient sample preparation, practical workflow design, rapid chromatographic separation, and appropriate technological innovation. Such an integrated approach could facilitate the development of next-generation methods for paraben determination that combine reliable analytical performance with improved environmental sustainability, practical applicability, and methodological innovation. Although this study provides valuable insights into the characteristics of chromatographic methods for paraben determination in cosmetic products and supports method selection based on environmental sustainability, practical applicability, and innovation, the exclusion of nine articles whose full texts were inaccessible represents a potential limitation. Their eligibility could not be confirmed against the predefined criteria, and their exclusion may have introduced selection bias. Therefore, the findings should be interpreted in light of this limitation.

## 3. Materials and Methods

OpenAI ChatGPT (GPT-6) was used to assist in creating Figure 2. The authors reviewed the generated image and take responsibility for the final graphical content. The prompt used was: “Create a scientific infographic with three overlapping circles representing MoGAPI, BAGI, and IMOGI, converging on a balanced analytical method and comprehensive comparison. Use relevant icons, key criteria, coordinated colors, and a clean, publication-ready design.”

### 3.1. Method Selection and Literature Search

**Figure 3:**
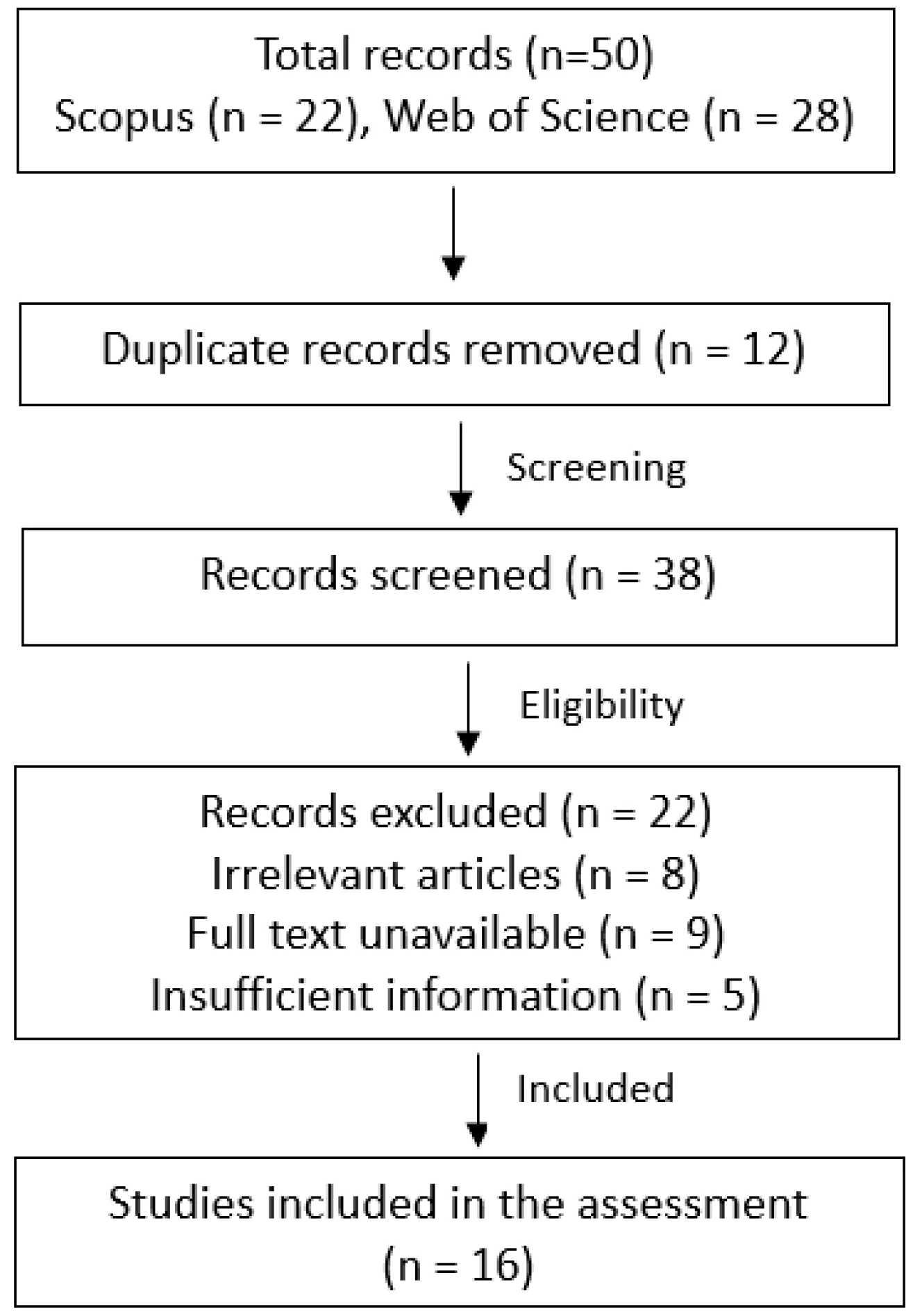
PRISMA flow diagram of the literature search for liquid chromatographic methods for paraben determination in cosmetic products.

A systematic literature search was conducted to identify published chromatographic methods for the determination of parabens in cosmetic and personal care products. The search covered the period from 2020 to 2026 to capture recent developments in paraben analysis, particularly advances in sustainable sample preparation, practical laboratory implementation, and innovative and modern analytical technologies. The search was performed using Scopus and Web of Science on 5 May 2026 using the search terms paraben, cosmetic, and liquid chromatography, adapted to the syntax of each database. The search strings are provided in Supplementary Table S5. Only English-language publications were considered. Studies were eligible for inclusion if they (i) reported an experimental chromatographic method for determining one or more parabens, (ii) applied the method to cosmetic or personal care products, and (iii) provided sufficient methodological information to permit assessment using the selected evaluation tools. Records were excluded if they (i) did not address paraben determination, (ii) focused on products outside the specified product categories, (iii) did not report a relevant chromatographic method, (iv) lacked sufficient information for the planned assessment, or (v) had inaccessible full texts. The search yielded 50 records for screening, comprising 22 articles from Scopus and 28 from Web of Science. After removal of 12 duplicate records, 38 unique records remained. Titles and abstracts were examined for relevance, and 8 articles were excluded because they were not relevant to the determination of parabens in cosmetic products. The remaining 30 articles were assessed for eligibility. Nine articles were excluded because their full texts could not be accessed despite attempts to obtain them. Consequently, their eligibility could not be confirmed against the predefined inclusion and exclusion criteria. An additional five articles were excluded because they did not contain sufficient information required for the application of the selected assessment tools. Consequently, 16 articles reporting chromatographic methods for paraben determination in cosmetic and personal care products were included in the final multidimensional assessment. The study selection process is summarized in the PRISMA-style flow diagram (Figure 3).

### 3.2. Multidimensional Assessment of the Selected Methods

The environmental sustainability of the selected methods was evaluated using the Modified Green Analytical Procedure Index (MoGAPI) [18]. MoGAPI was selected because it provides a structured evaluation of the environmental characteristics of an analytical procedure by considering multiple aspects of the analytical workflow, including sample preparation, reagents and solvents, occupational hazards, and waste generation. Each method was evaluated according to the criteria defined by the MoGAPI framework, and the corresponding scores were calculated according to the established scoring procedure. The resulting scores were expressed on a scale of 0–100, with higher scores indicating a more favorable environmental profile [18].

The practical applicability of the selected methods was evaluated using the Blue Applicability Grade Index (BAGI) [19]. BAGI provides a standardized assessment of the practical aspects of analytical procedures by considering factors associated with their implementation under laboratory conditions. The evaluation considered the operational characteristics of each method, including sample amount, analysis time, sample-preparation requirements, equipment and material requirements, availability of reagents and solvents, procedural simplicity, and other factors influencing routine implementation. The criteria were scored according to the BAGI methodology, and the resulting scores were expressed on a 0–100 scale, with higher scores representing greater practical applicability.

The degree of innovation and modernization of the selected analytical methods was evaluated using the Innovative Method Orange Gradient Index (IMOGI) [20]. IMOGI was developed to characterize the extent to which analytical procedures incorporate innovative and modern features across different stages of the analytical workflow. The assessment considered the methodological features specified by the IMOGI framework, including developments in sample preparation, extraction strategies, automation, miniaturization, digitalization, and consideration of GAC principles. Each method was scored according to the established IMOGI criteria, with the final score expressed on a 0–100 scale. Scores ≥ 50 indicate a high degree of innovation and modernization.

The three assessment scores were considered as complementary dimensions because environmental sustainability, practical applicability, and innovation represent conceptually distinct characteristics of analytical methods. The results were presented graphically to facilitate comparison of the multidimensional profiles of the selected methods and to identify distinctive methodological characteristics and opportunities for further improvement.

## 4. Conclusions

This study provided a multidimensional assessment of 16 chromatographic methods for paraben determination in cosmetic products using MoGAPI, BAGI, and IMOGI to evaluate environmental sustainability, practical applicability, and innovation and modernization, respectively. Most methods showed relatively favorable environmental sustainability, mainly due to reduced solvent consumption, alternative extraction media, and waste minimization. Practical applicability varied more substantially, with sample amount, analysis time, sample-preparation simplicity, and accessibility of materials being important factors. Innovation was demonstrated through miniaturized extraction, advanced sorbents, alternative solvent systems, and modern chromatographic and detection strategies.

The combined assessment highlights that environmental sustainability, practical applicability, and innovation represent complementary characteristics that should be considered together when developing analytical methods. Instead, future development should integrate sustainability, practicality, and innovation from the initial method-design stage. In particular, miniaturization, environmentally preferable solvents and sorbents, rapid separation, and GAC-oriented workflow design represent important directions for advancing next-generation methods for paraben analysis. Overall, the combined use of MoGAPI, BAGI, and IMOGI provides a structured framework for benchmarking existing methods; identifying methodological strengths and opportunities for improvement; and supporting the development of more sustainable, practical, and innovative chromatographic approaches for cosmetic analysis.

## Figures and Tables

**Table 1:** Characteristics of the selected chromatographic methods for the determination of parabens in cosmetic products.

No.	Matrix	Analytes	Separation Technique	Extraction	Solvents and Reagents	LOD	Run Time	Sample Size
1 [22]	Body cream	MP, EP, PP, BP, BzP	HPLC-UV	DLLME	Methanol, acetonitrile, acetone, trichloromethane	0.01 to 0.04% *w*/*w*	28 min	100 mg
2 [23]	Hair masks, shampoos, and hair conditioners	MP, EP, PP, BP, BzP, iPP, iBP, triclosan, dibutyl phthalate, diethyl hexyl phthalate	HPLC-UV	Solvent extraction	Ethanol, trifluoroacetic acid, hexane, acetonitrile, tetrahydrofuran	0.052 µg mL^−1^ 1.744 µg mL^−1^	25 min	25 mL
3 [24]	body lotion, skin toner, facial cream, facial cleanser, liquid shampoo, body wash, cleansing water, makeup base, sunscreen, lipstick, essence, perfume, eye cream, hair conditioner, hand cream, mouthwash, and eyewash	MP, EP, PP, BP, iPP, iBP	HPLC-UV	MIPs	Methanol, acetonitrile, ethanol, acetic acid, toluene, ethylene glycol dimethacrylate, azoisobutylnitrile, glycidilmethacrylate,	0.2–0.3 ng mL^−1^	20 min	1 g
4 [25]	Mouthwashes	MP, EP, PP, BP	HPLC-UV	DES-VALLME	Menthol, decanoic acid, acetonitrile	4.6–6.1 µg L^−1^	30 min	5 mL
5 [26]	Lotion and facial cleanser	MP, EP, PP, BP	HPLC-UV	IL-DLLME	Methanol, acetonitrile, ethanol, Fe_3_O_4_@GO, HCl	0.064–0.135 µg mL^−1^	8 min	5 g
6 [27]	Facial creams, body milks, body lotions, shampoos, makeup powders, hand creams	MP, PP	HPLC-UV	Solvent extraction	Methanol, acetonitrile, trifluoroacetic acid, sulfuric acid	Not reported	6.2 min	0.5 g
7 [28]	Shampoos, hair conditioners, creams, antiperspirants	MP, EP, PP, BP	UHPLC-DAD	NADES-DLLME	Menthol, formic acid	0.17–0.33 ng mL^−1^	6 min	0.5 g
8 [29]	Cosmetic (blush) and food (candy)	MP, EP, PP, BP	Superheated water HPLC-UV	Solvent extraction	Ethanol	1.5–28 µg g^−1^	8 min	0.5 g
9 [30]	Skin cream, sunblock cream, hand cream and foot cream	MP, EP, PP, BP	HPLC/PDA	MSPE	Methanol and acetic acid	0.19–0.31 µg L^−1^	8 min	5 mg
10 [31]	Toothpaste	MP, EP, PP, BP	HPLC-VWD	VA-DES-DLLME	Methanol, hexane, tetrabutylammonium chloride,	0.01–0.02 µg g^−1^	14 min	0.1 g
11 [32]	Skin lotions, creams and cleansers	MP, EP, PP	HPLC-UV	SPE	Methanol, acetonitrile, ethyl acetate, acetone, isooctane	0.0004–0.00043 µg g^−1^	14 min	0.05 g
12 [33]	Lipstick, mascara, face powder, face-hand moisturizing cream, baby shampoo, whitening cream, and hair gel	MP, EP, PP, BP, BzP, iBP	HPLC-UV	DμSPE	Methanol, acetonitrile, tetrahydrofuran	0.027–0.033 µg Kg ^−1^	10 min	0.5 g
13 [34]	Shampoos, masks, balms, lotions, hair spray and mousse	MP, EP, PP, BP, iPP	HPLC-UV	MEPS	Methanol, acetonitrile, acetic acid	2–5 µg L^−1^	15 min	100 mg
14 [35]	Shower gel, shampoo, soap, wet wipe, face powder	MP, EP, PP, BP, Isothiazolinones methylisothiazolinone and methylchloroisothiazolinone	UHPLC/DAD	SPE	Acetonitrile/methanol, trifluoroacetic acid	1–2 µg L^−1^	30 min	0.5 g
15 [36]	Tonic and micellar water	MP, EP, PP, BP, BzP, iPP	HPLC-FD	Solvent extraction	Methanol, o-phosphoric acid	290–320 µg L^−1^	90 s	40 µL
16 [37]	Lotion, oil, cream	MP, EP, PP, BP, iPP, iBP, phenoxyethanol, benzoic acid, salicylic acid, sorbic acid, dehydroacetic acid	HPLC-UV	Solvent extraction	Acetonitrile, methanol, ammonium formate, formic acid	100–1000 µg L^−1^	17 min	0.2 g

Methyl paraben (MP), ethyl paraben (EP), propyl paraben (PP), butyl paraben (BP), benzyl paraben (BzP), isopropyl paraben (iPP), and isobutyl paraben (iBP). HPLC: high-performance liquid chromatography; DLLME: dispersive liquid–liquid microextraction; MIPs: molecularly imprinted polymers; DES-VALLME: deep eutectic solvent-based vortex-assisted liquid–liquid microextraction; IL-DLLME: ionic liquid-dispersive liquid–liquid microextraction; UHPLC-DAD: ultra-high performance liquid chromatography–diode array detector; NADES: natural deep eutectic solvent; SPE: solid-phase extraction; MSPE: magnetic solid-phase extraction; VA-DES-DLLME: vortex assisted liquid-liquid microextraction; HPLC-VWD: high-performance liquid chromatography coupled with variable wavelength detector; DμSPE: dispersive μ-solid phase extraction; MEPS: microextraction by packed sorbent; FD: fluorescence detection.

**Table 2:** Assessment of the selected chromatographic methods using MoGAPI, BAGI, and IMOGI.

No.	MoGAPI	BAGI	IMOGI	Ref.
1	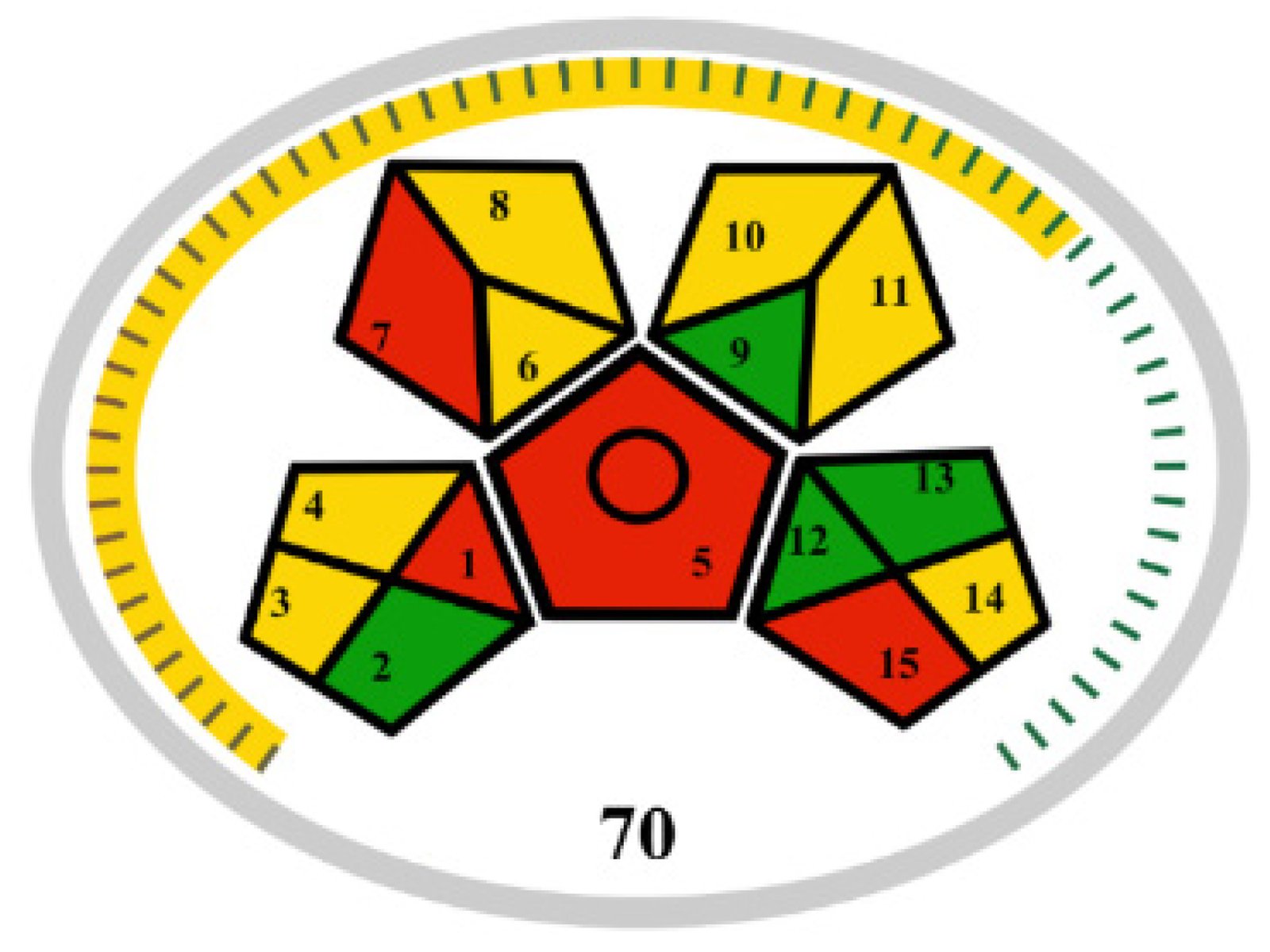	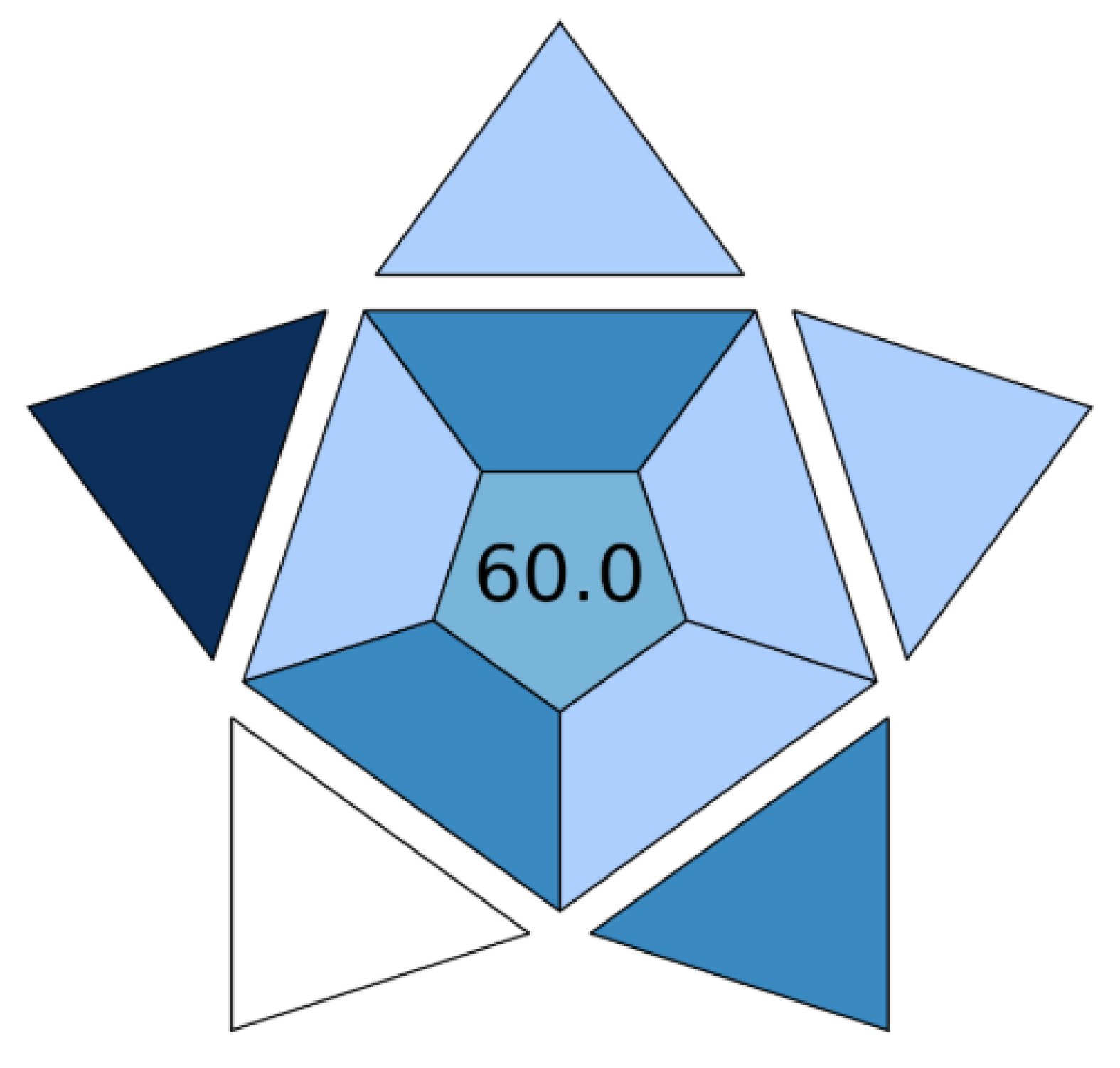	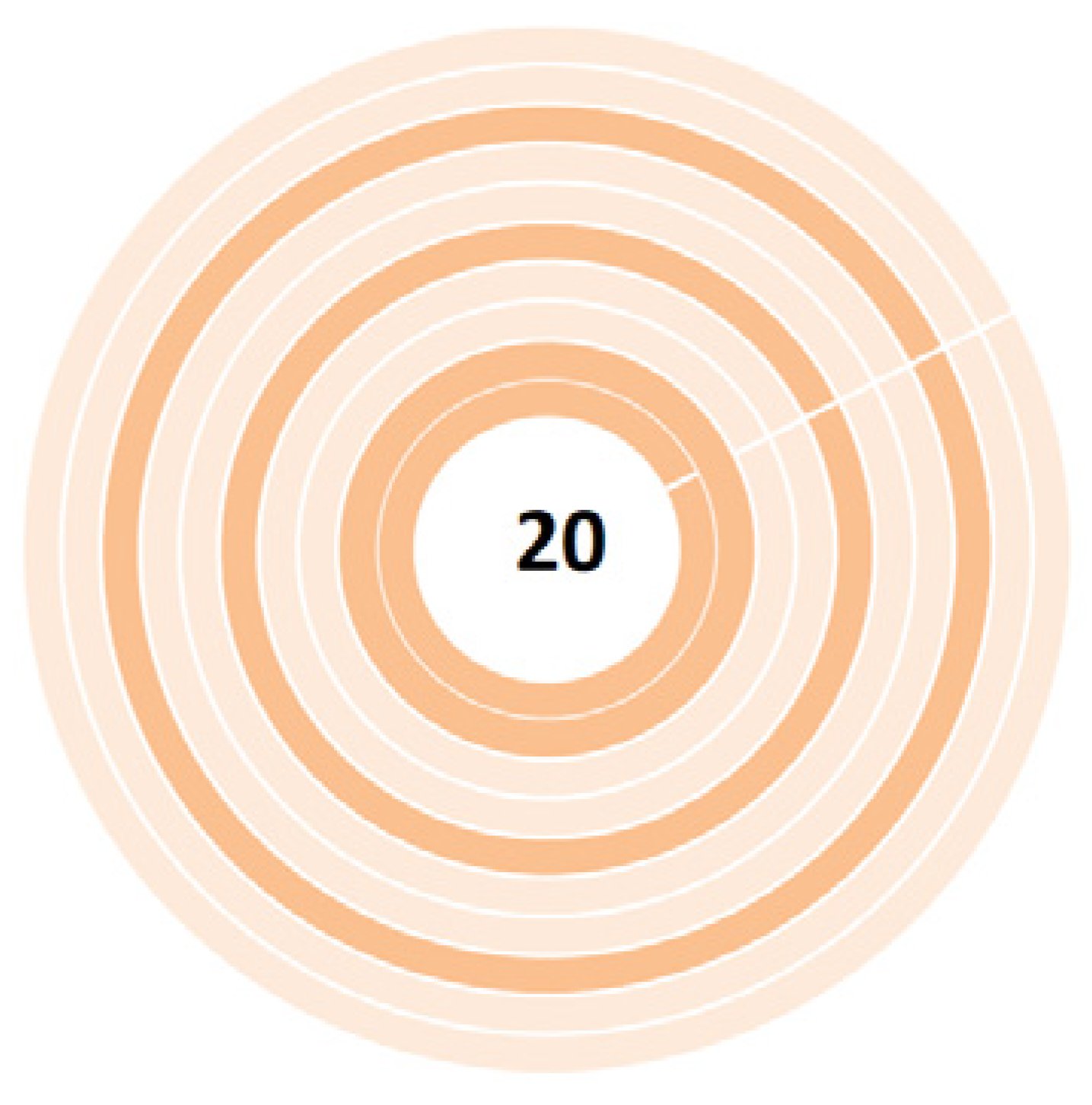	[22]
2	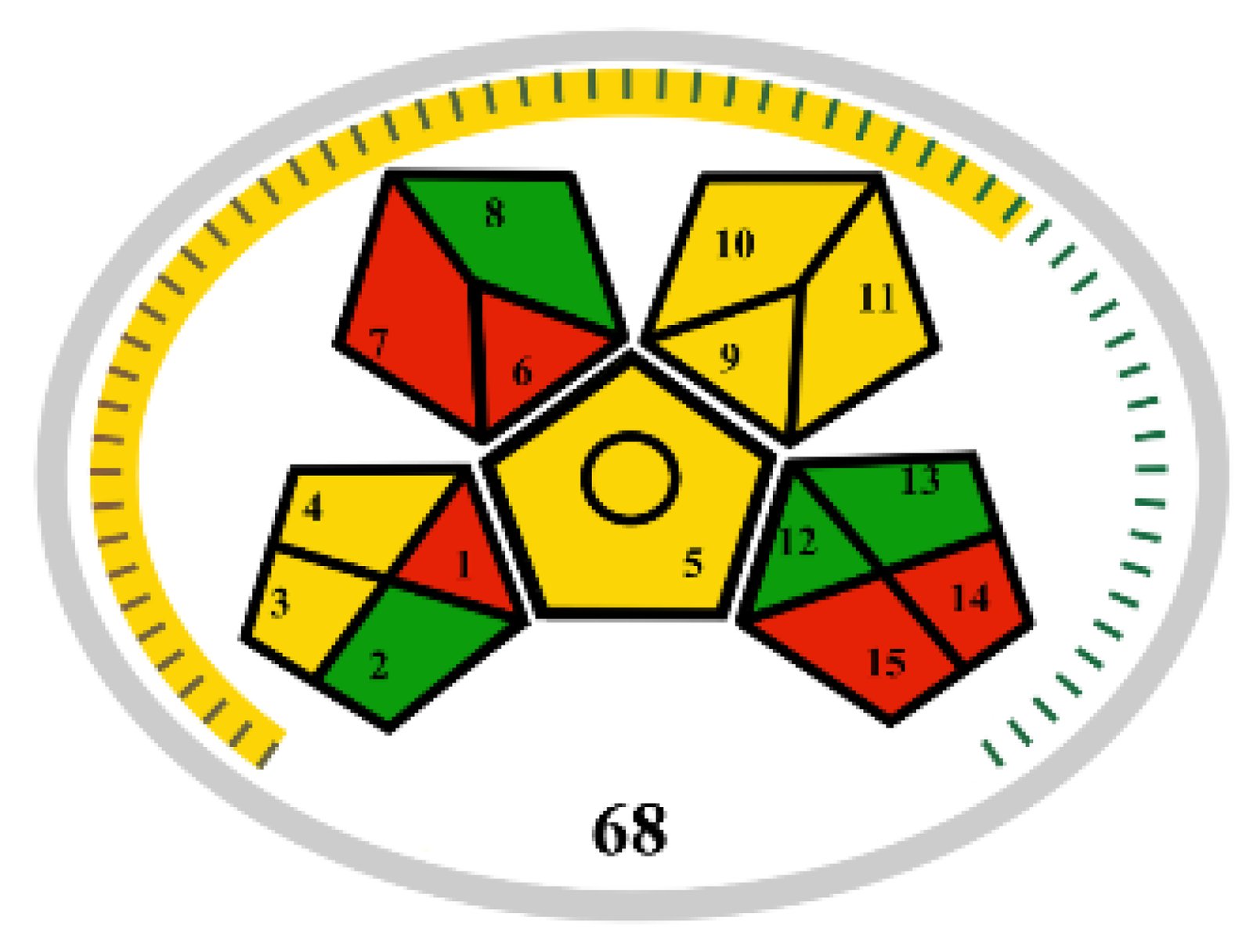	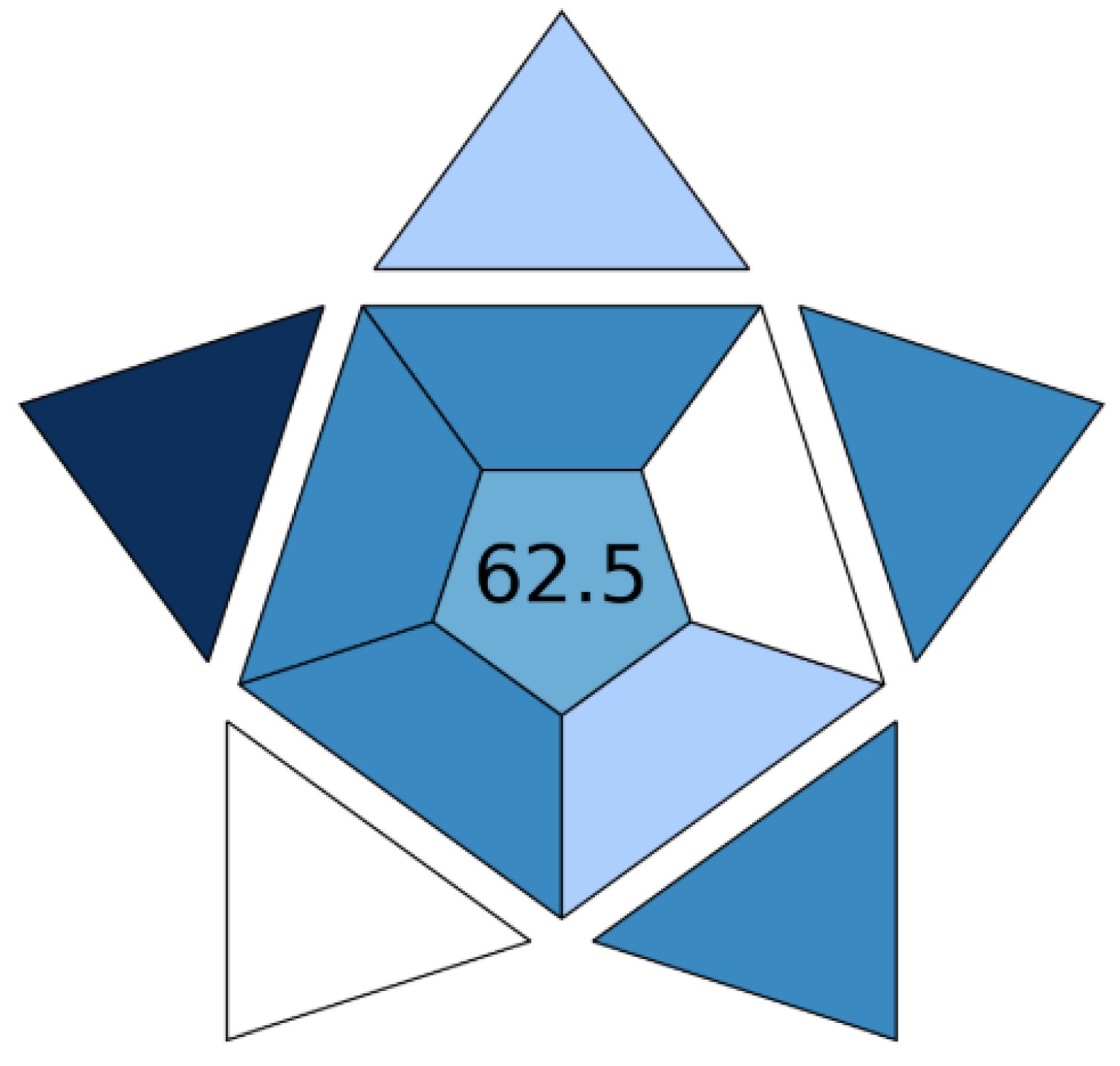	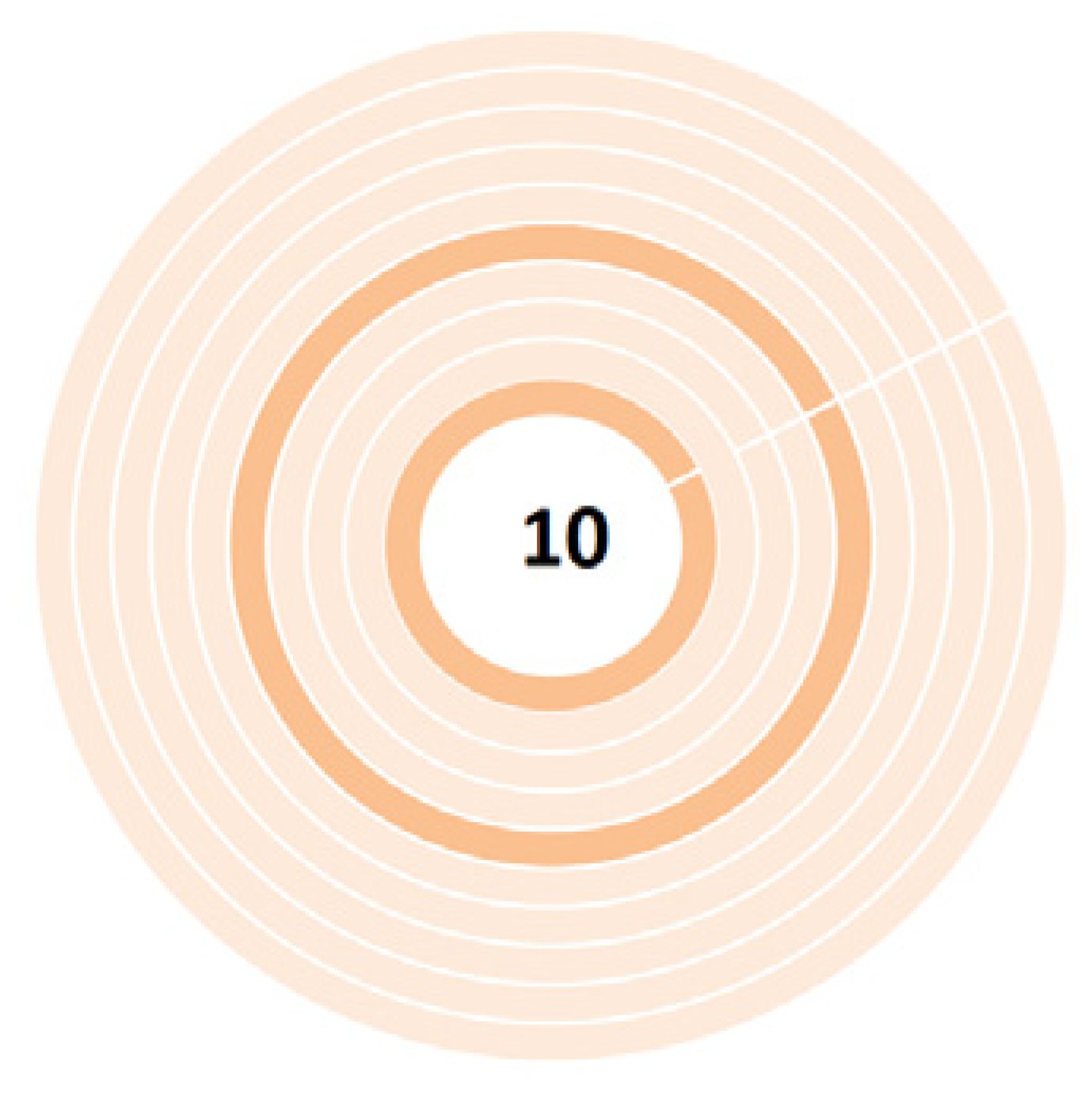	[23]
3	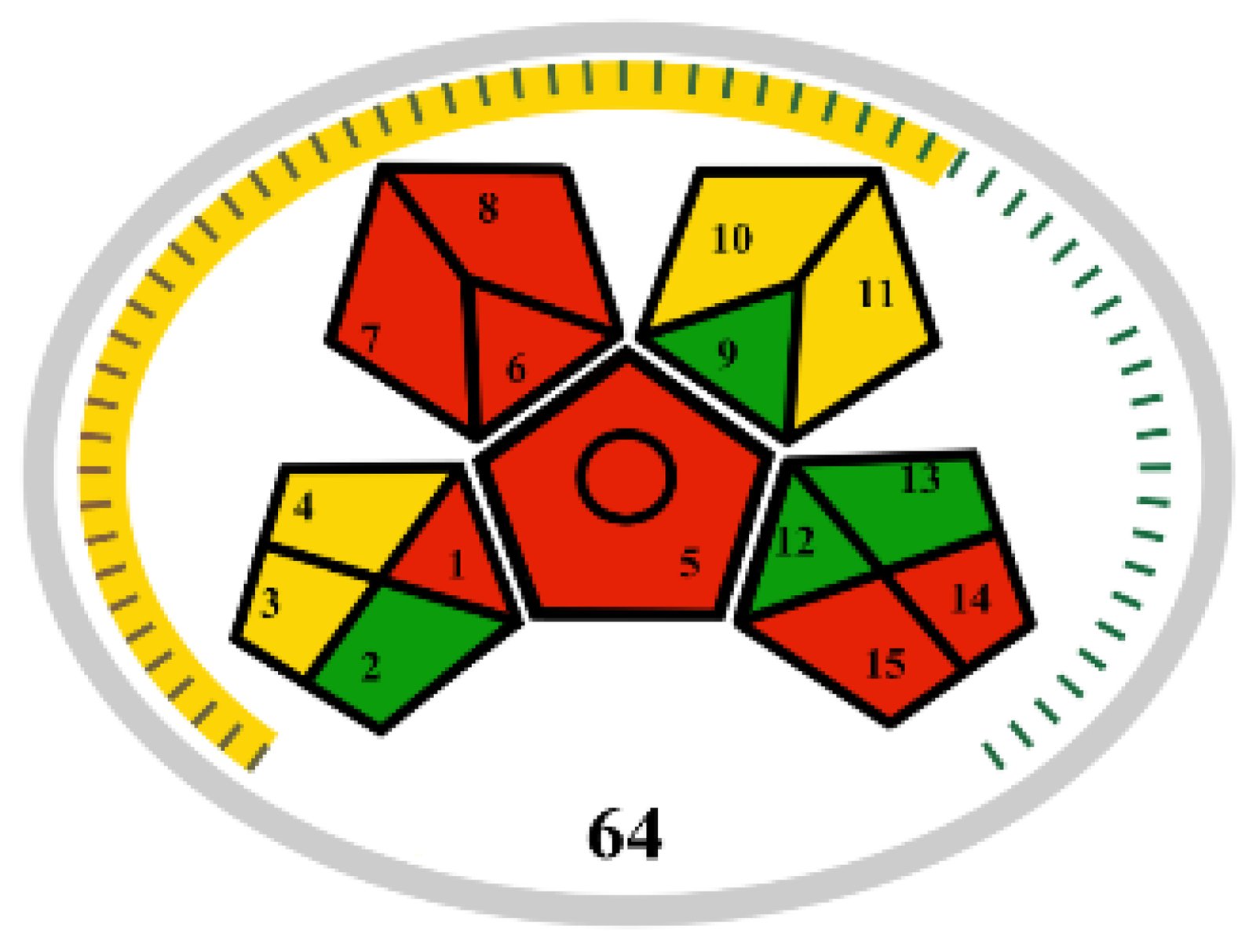	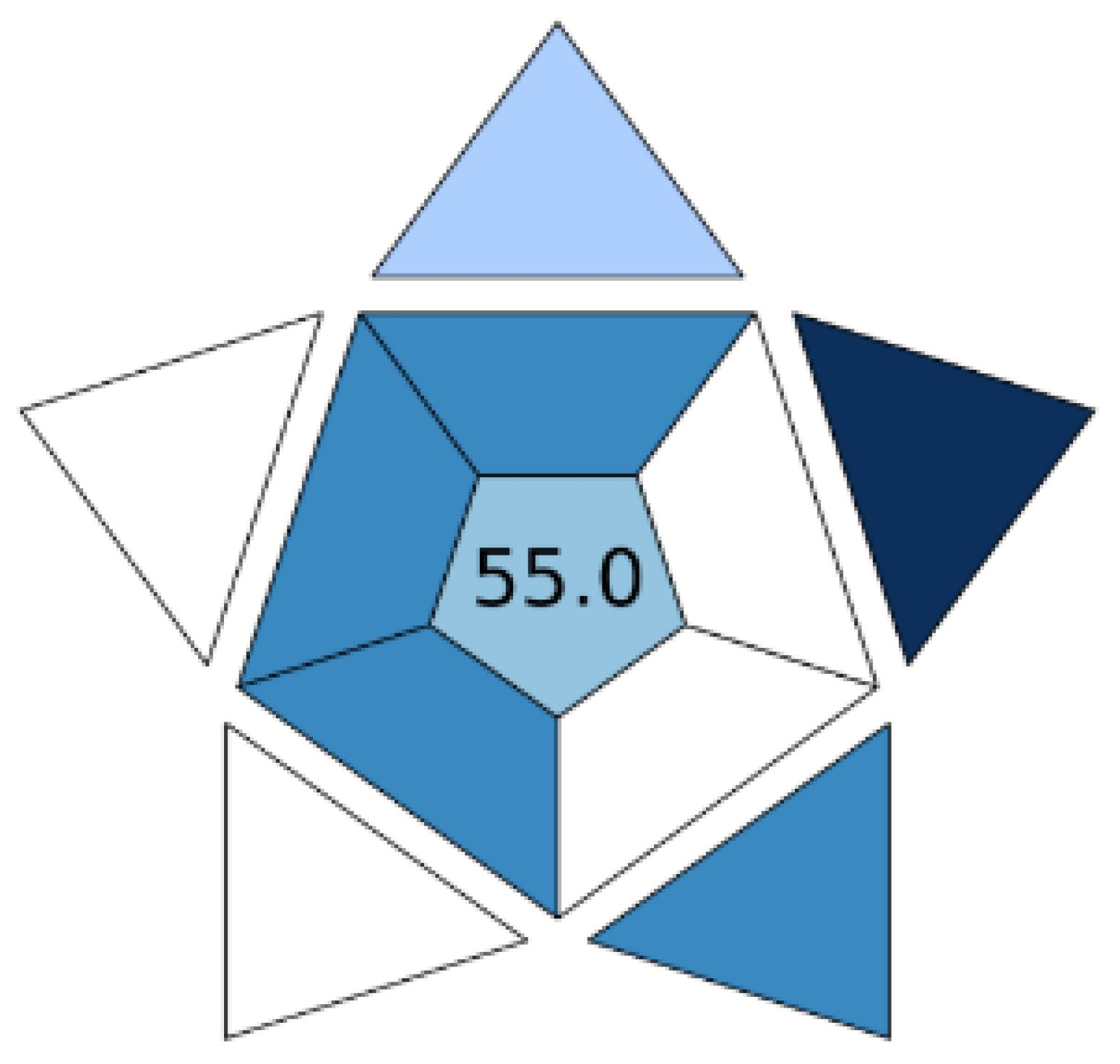	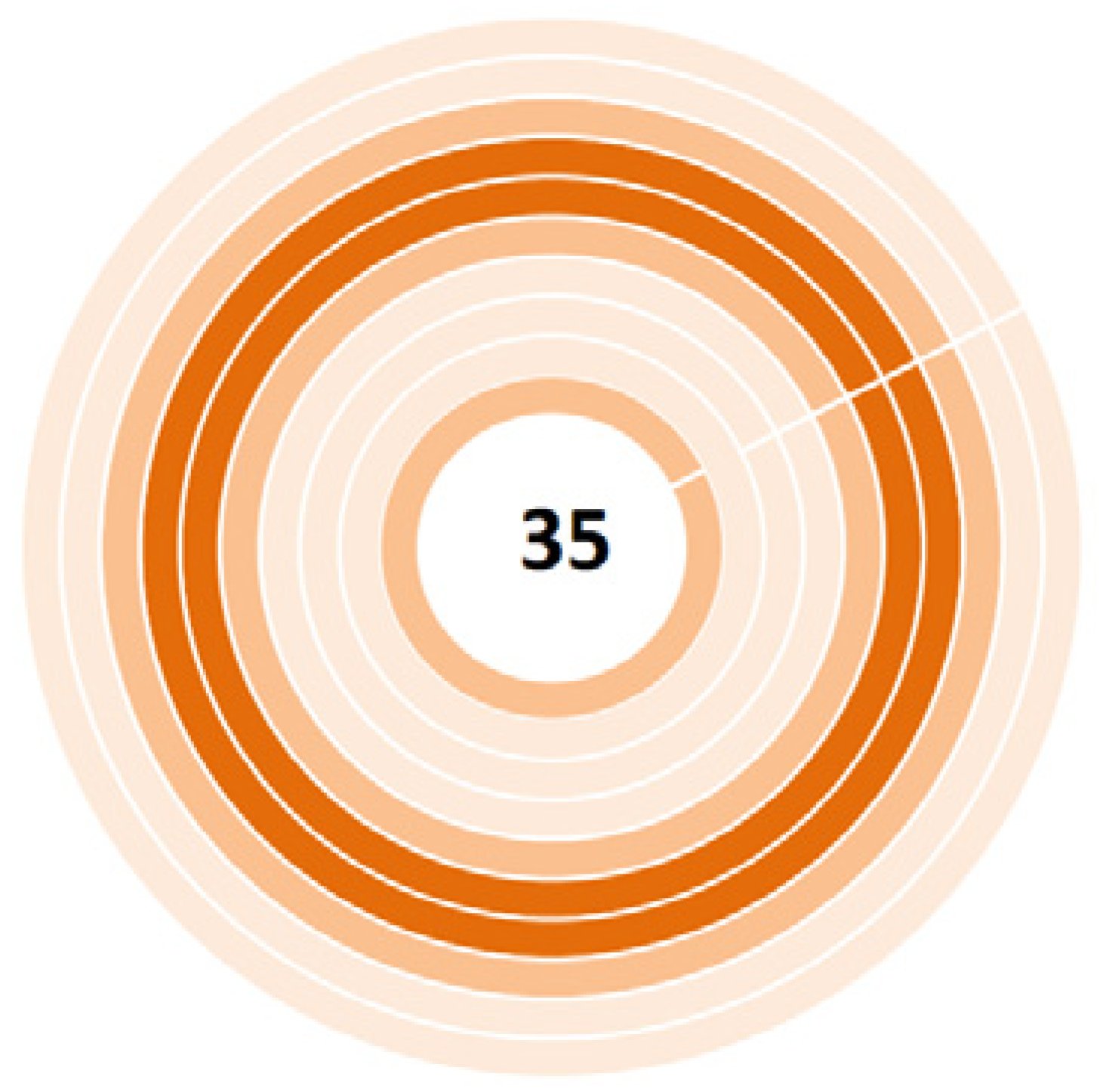	[24]
4	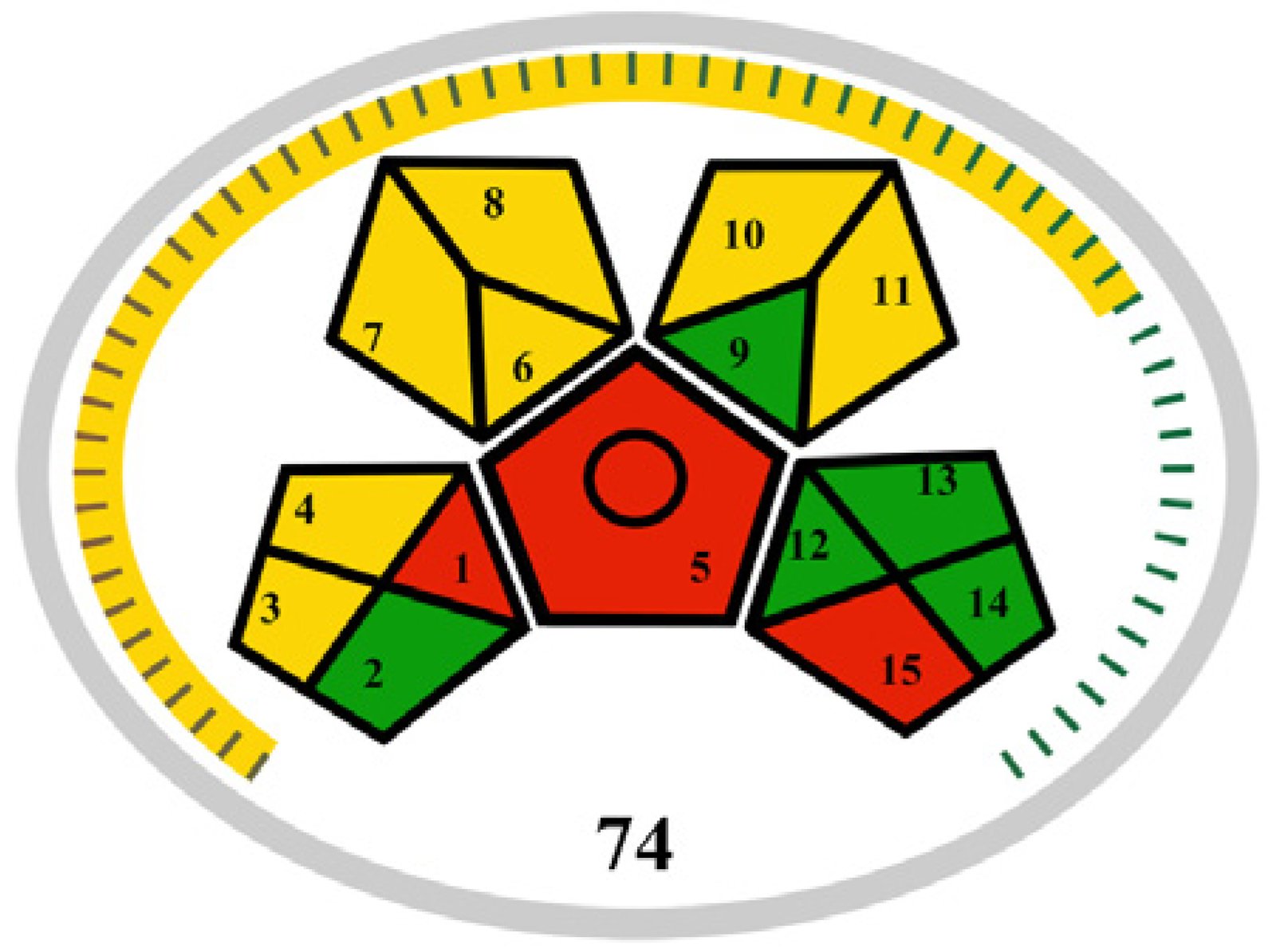	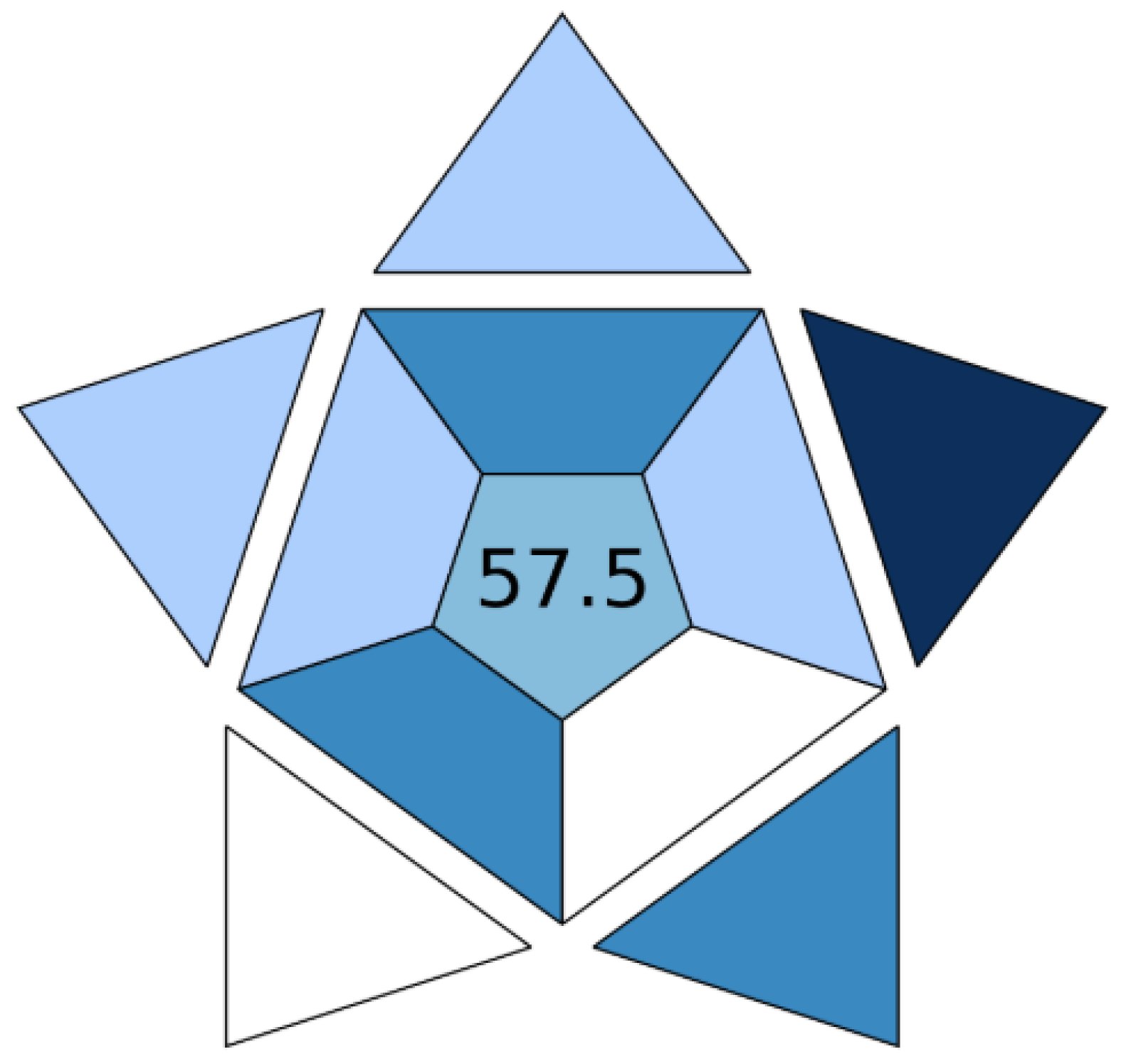	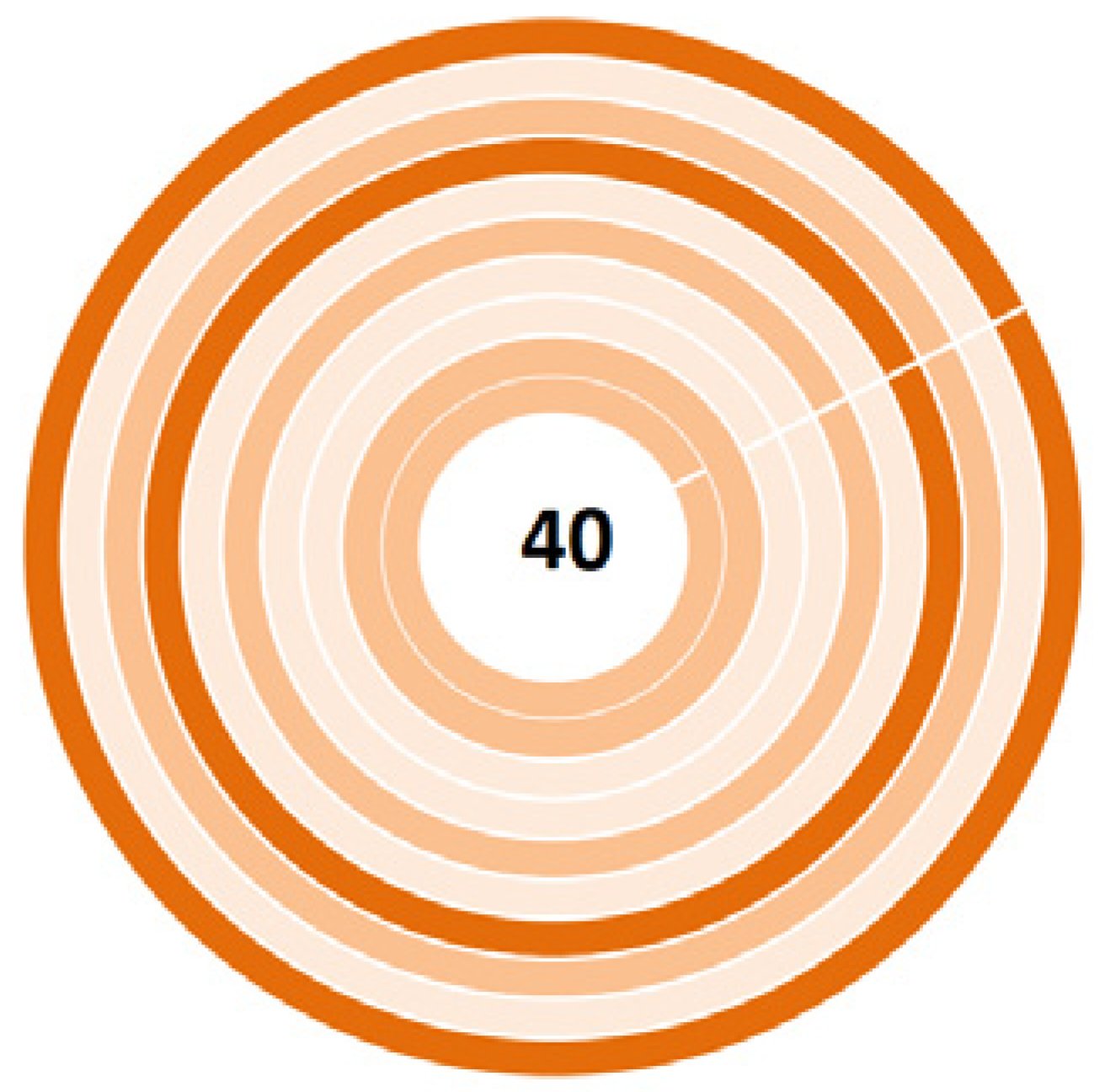	[25]
5	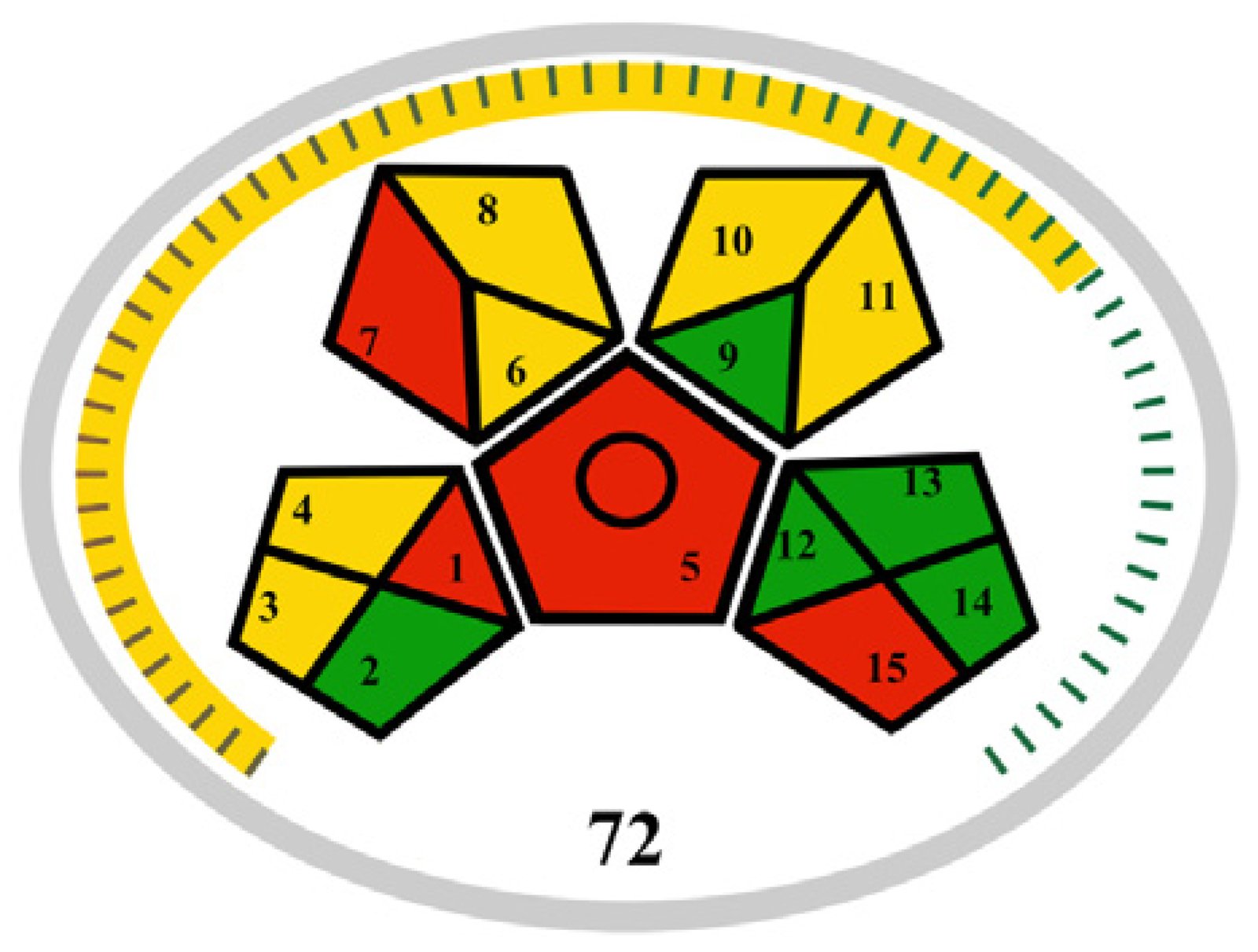	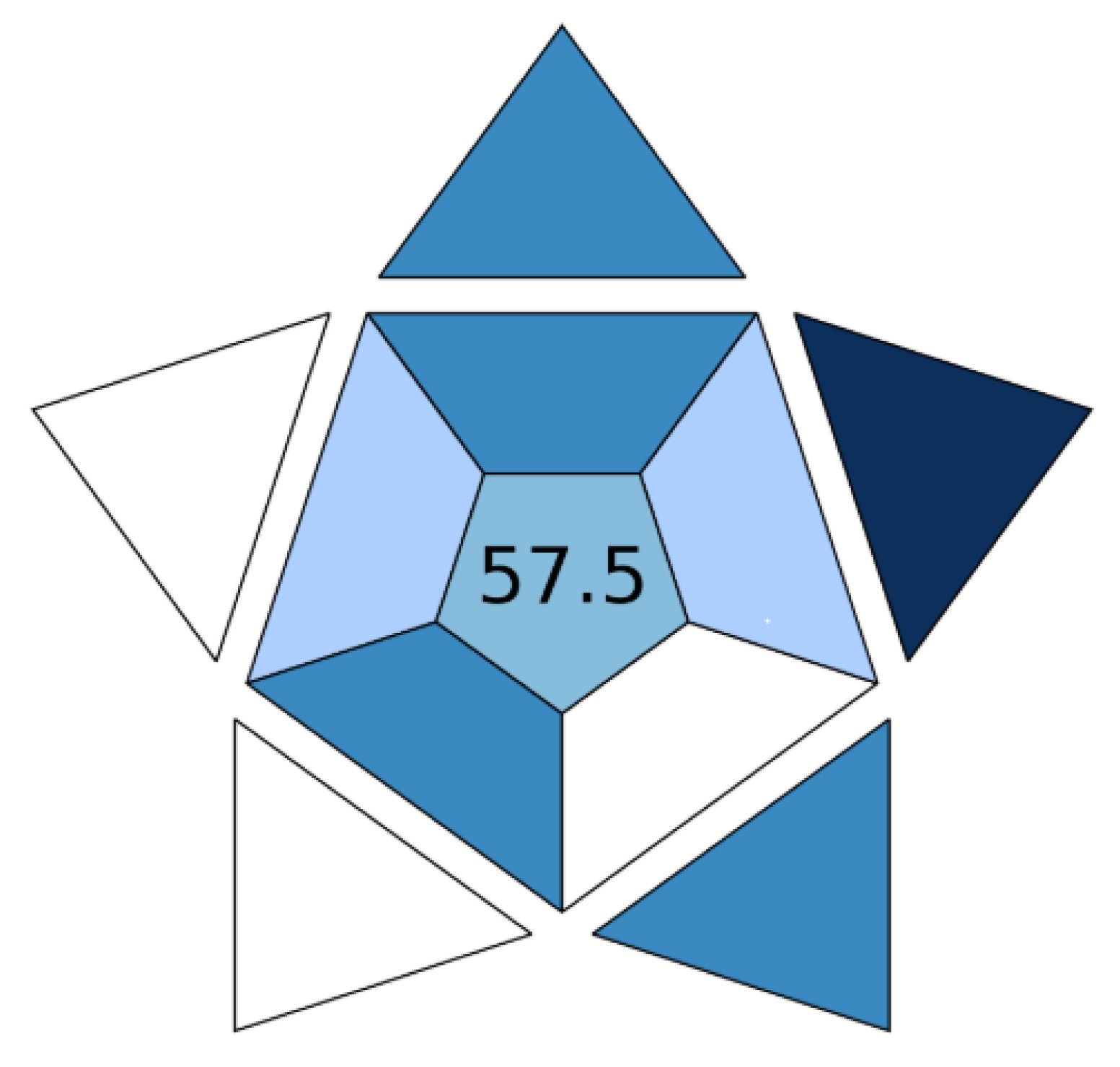	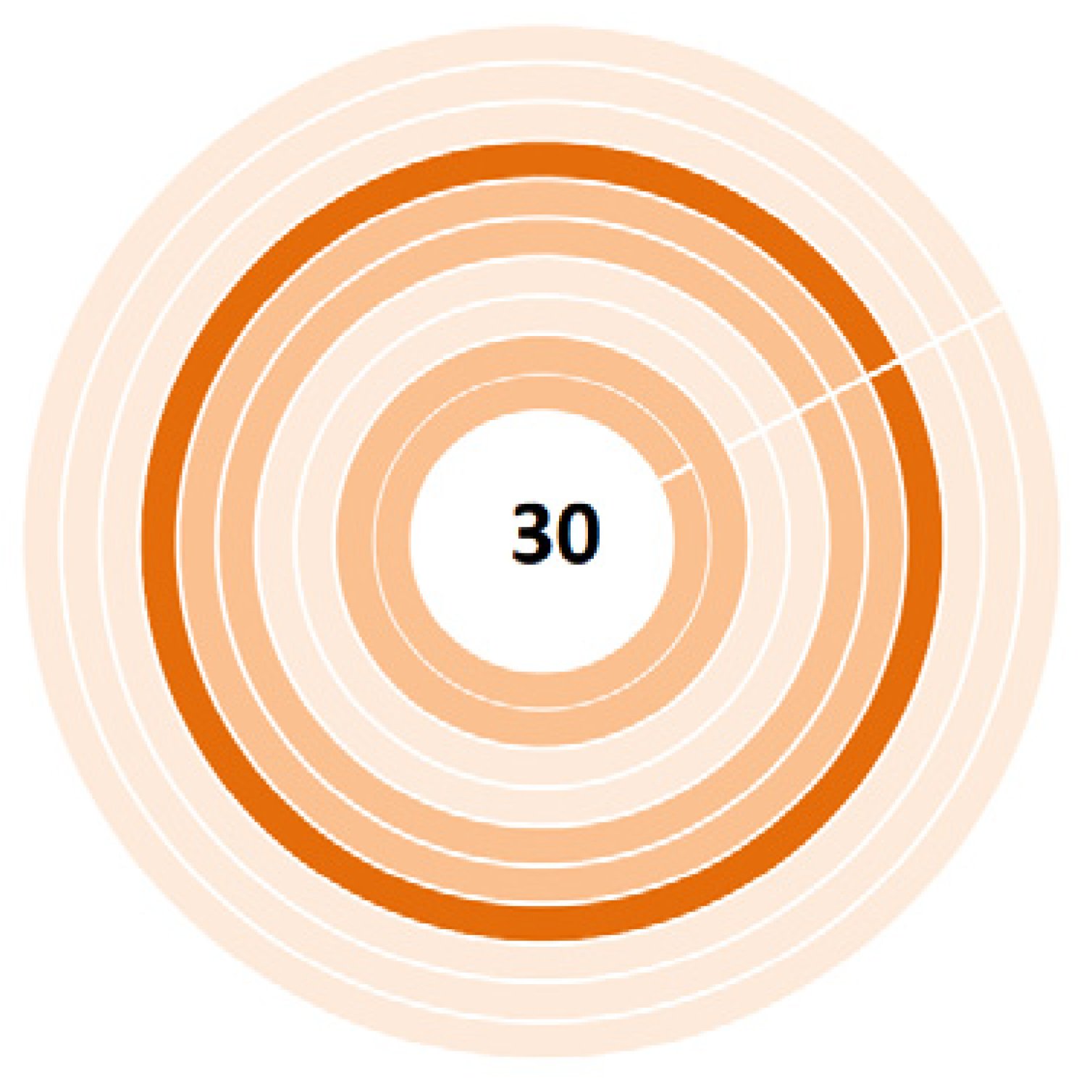	[26]
6	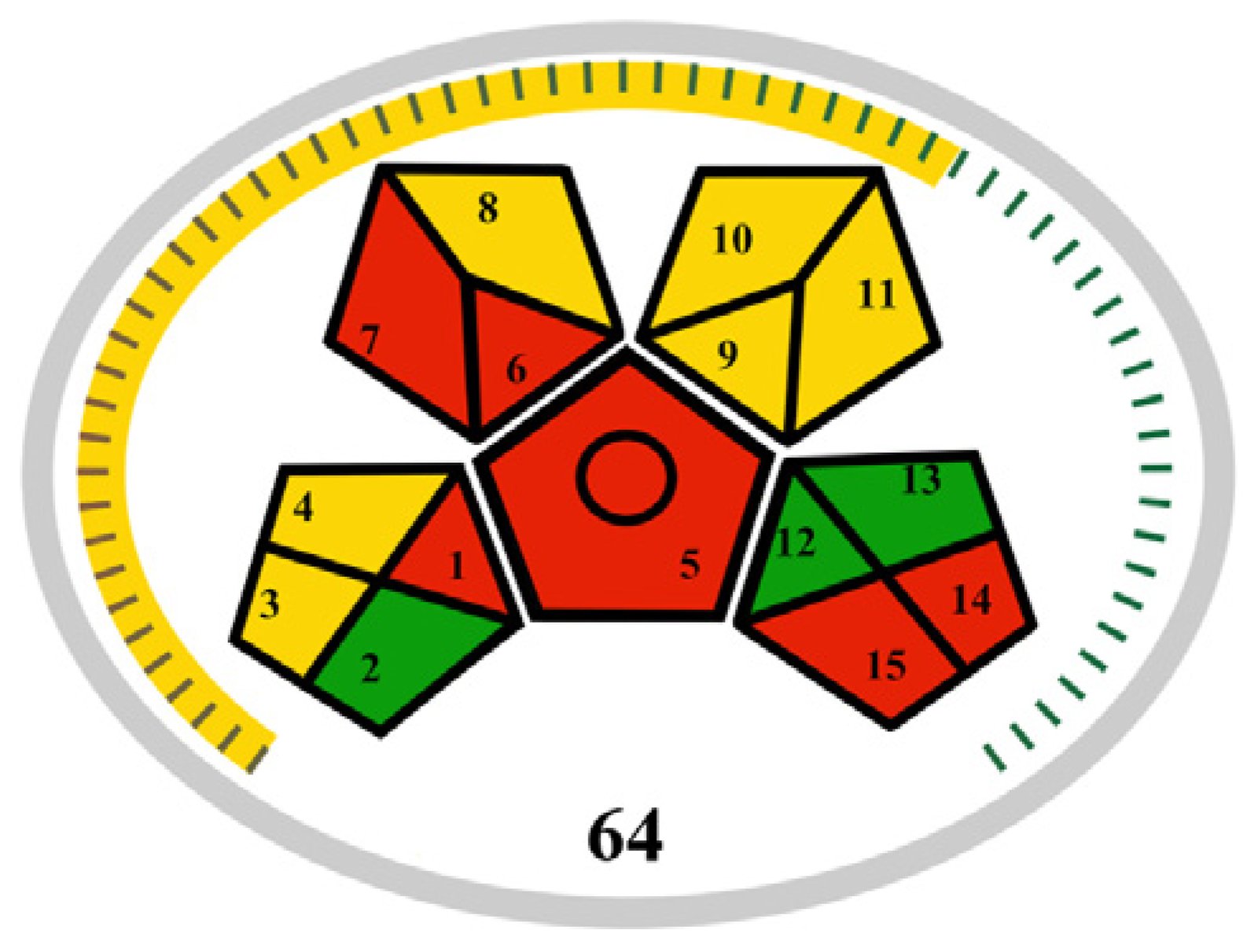	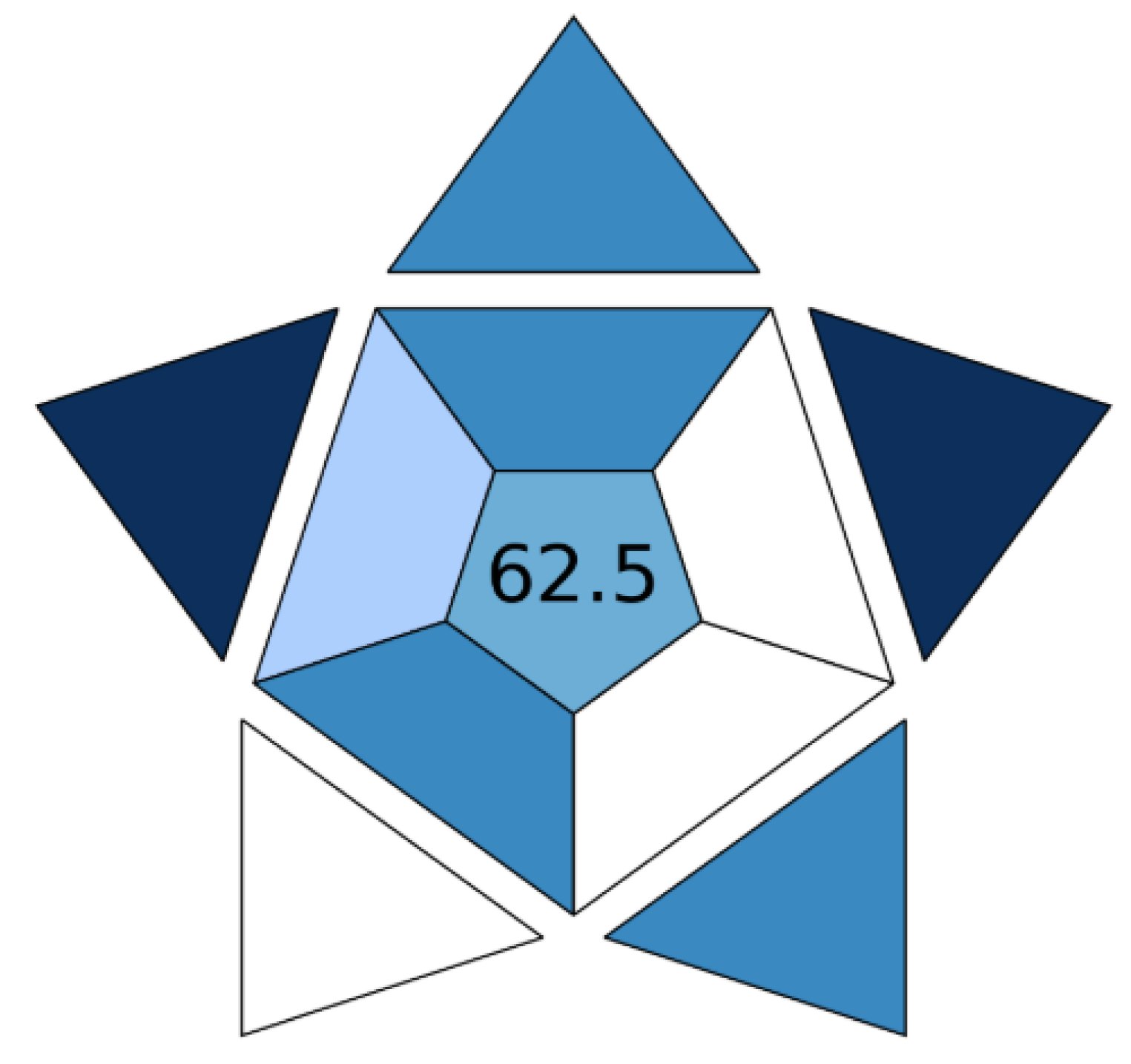	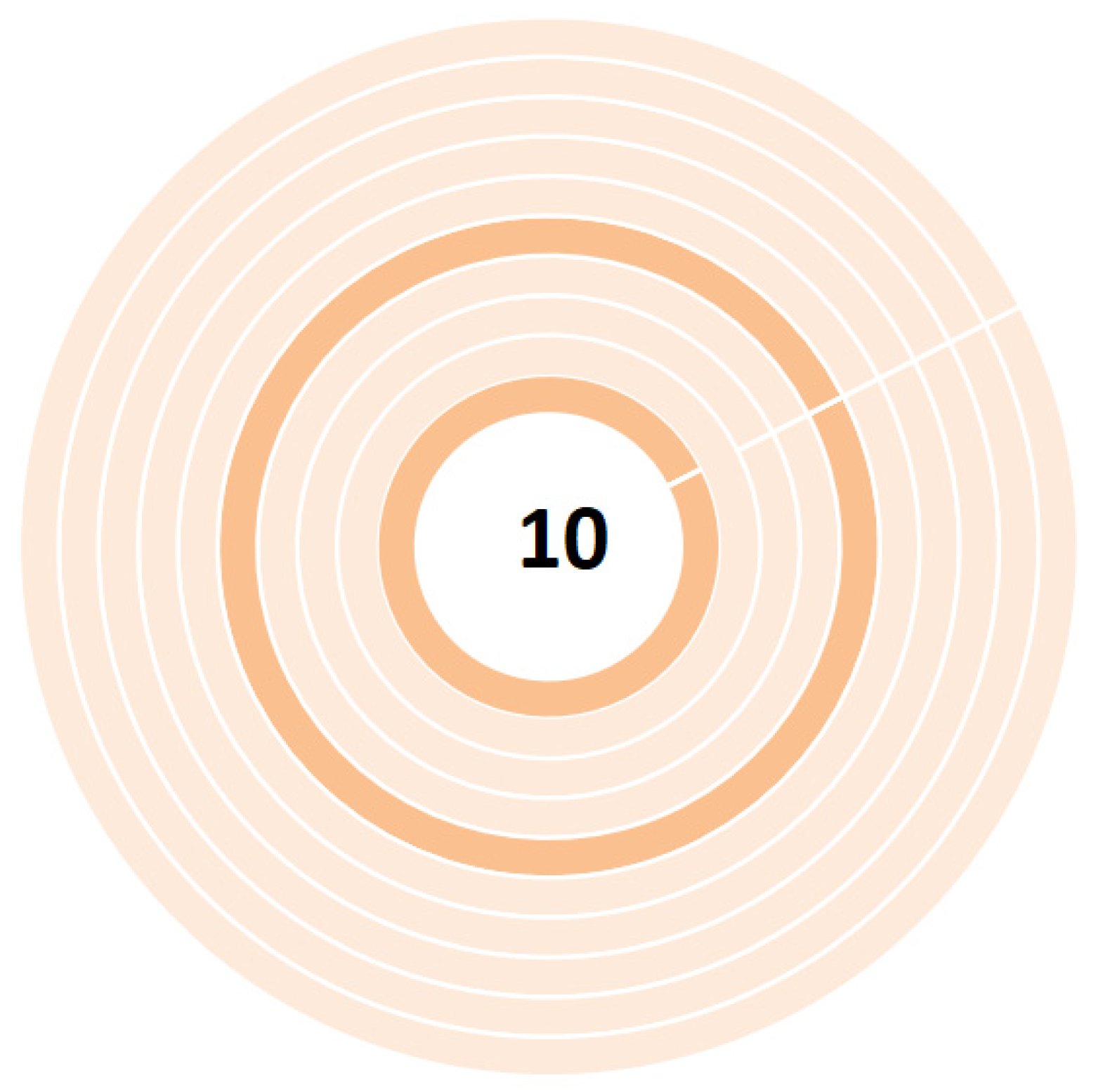	[27]
7	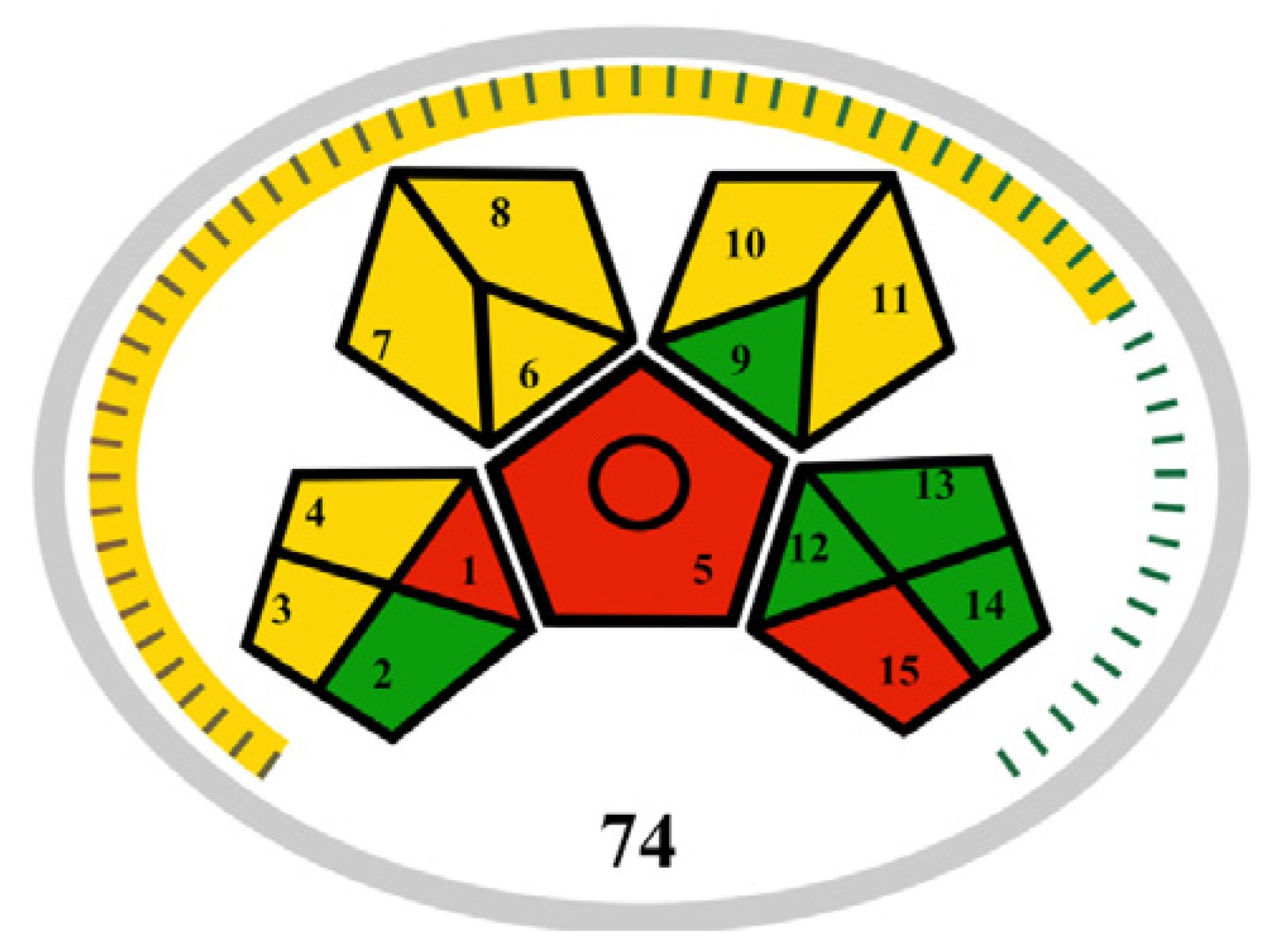	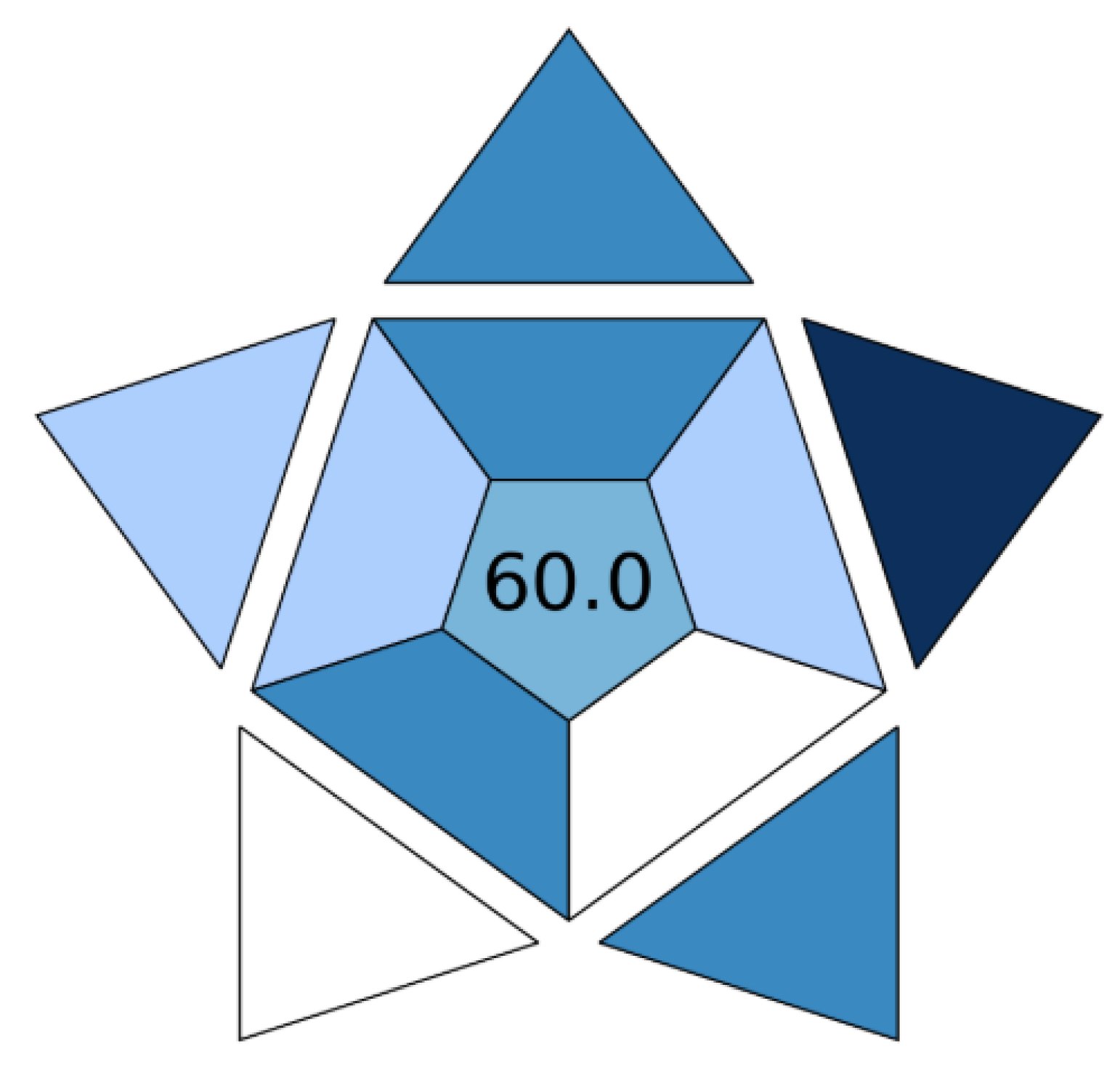	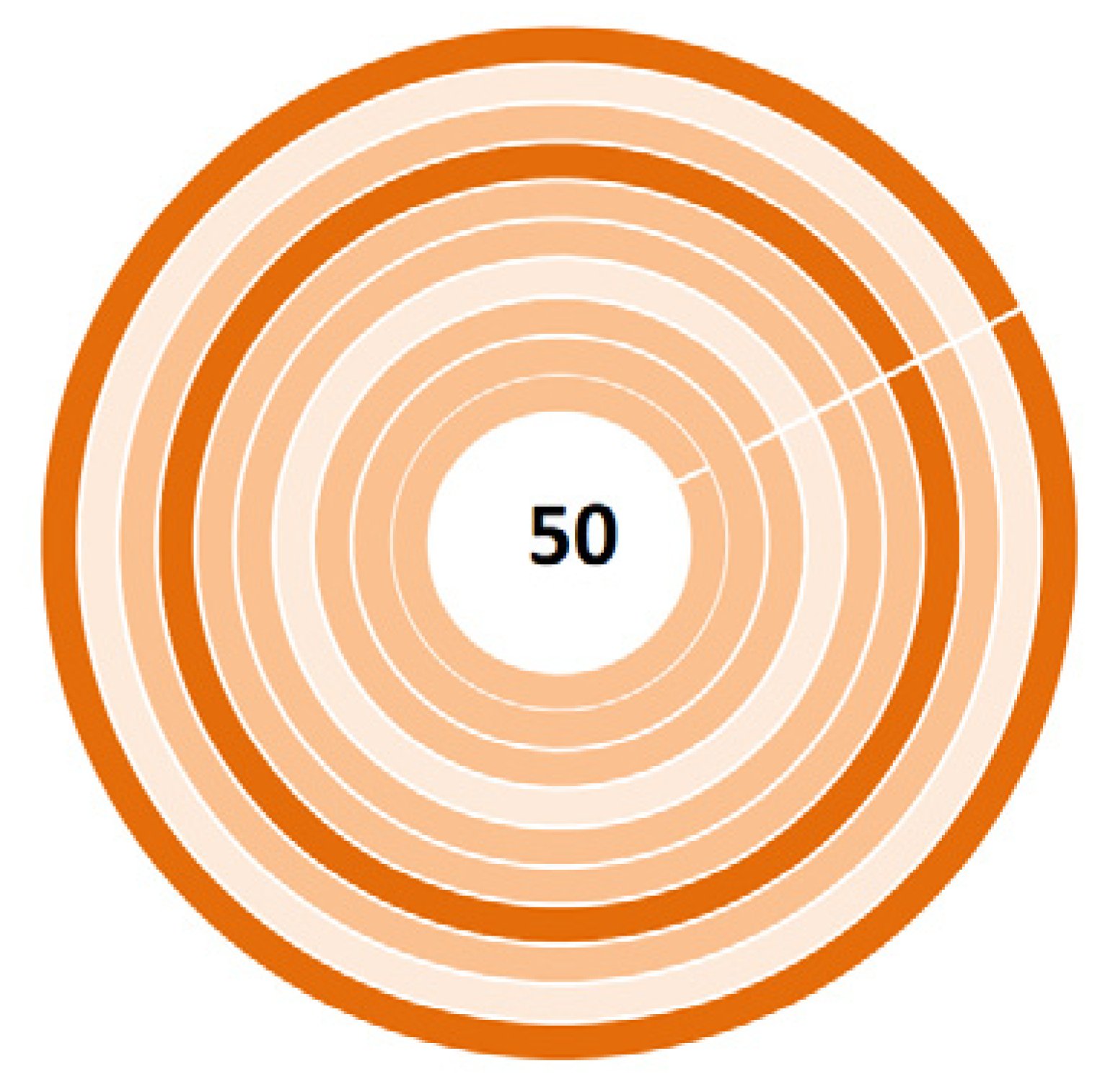	[28]
8	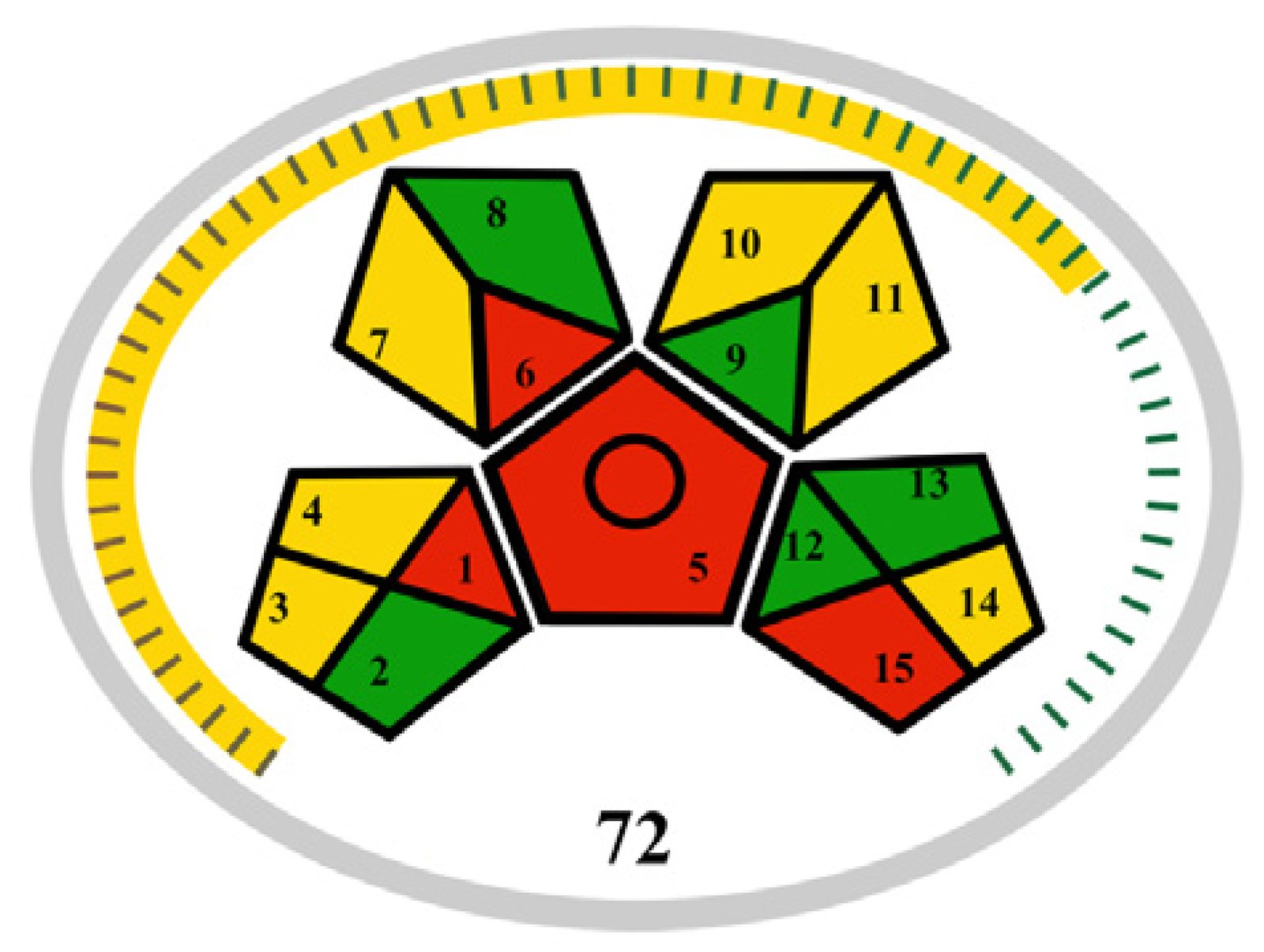	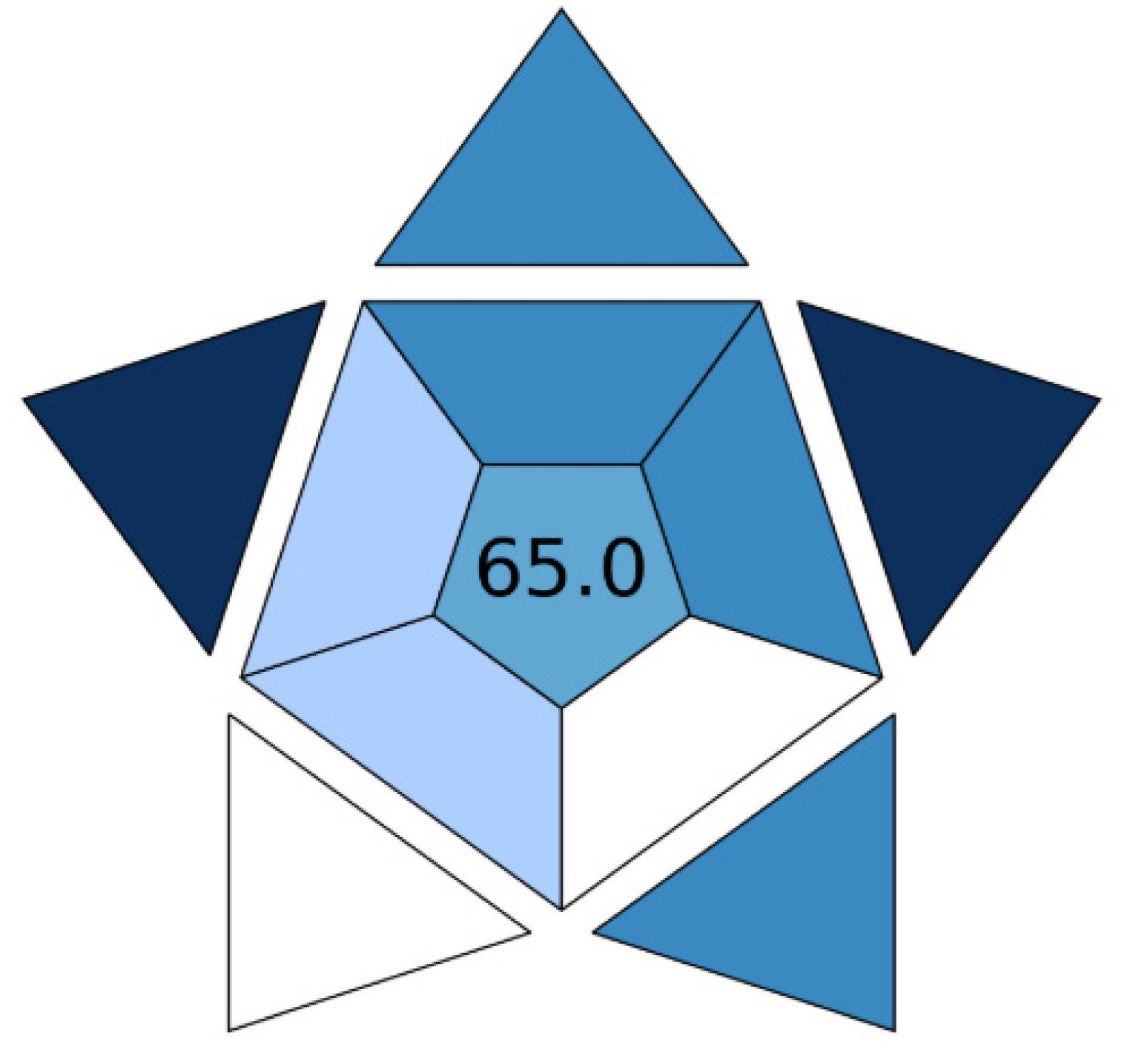	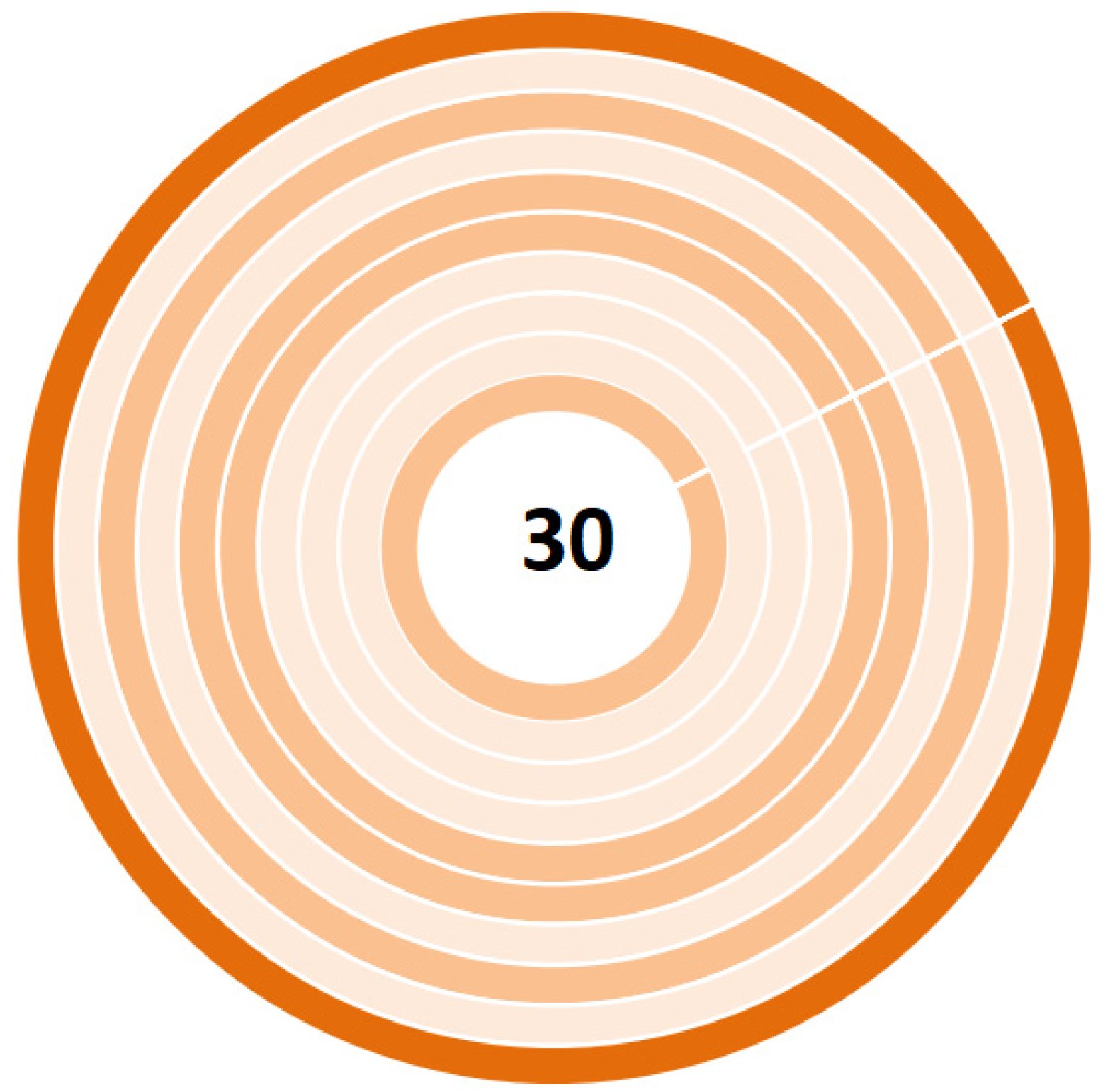	[29]
9	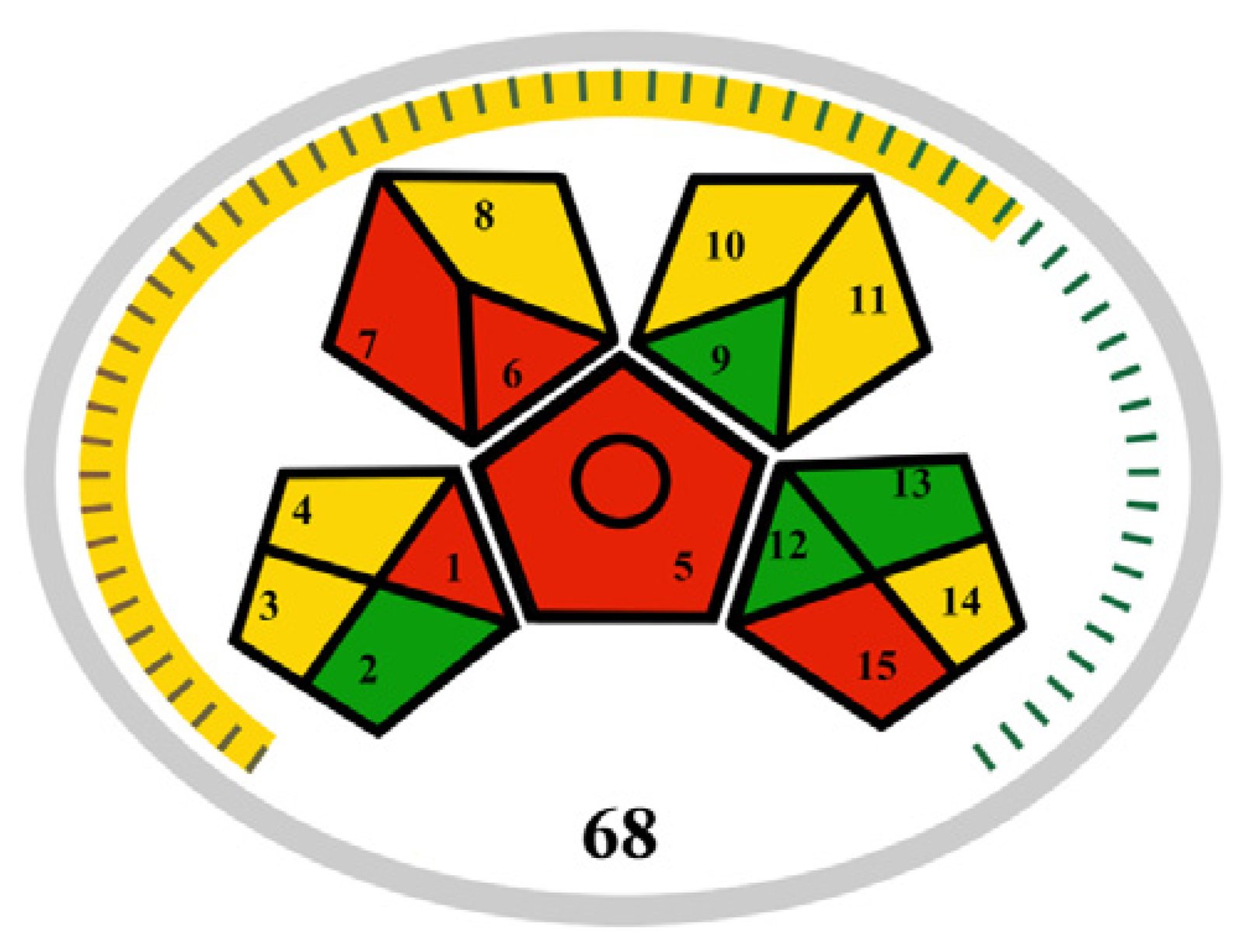	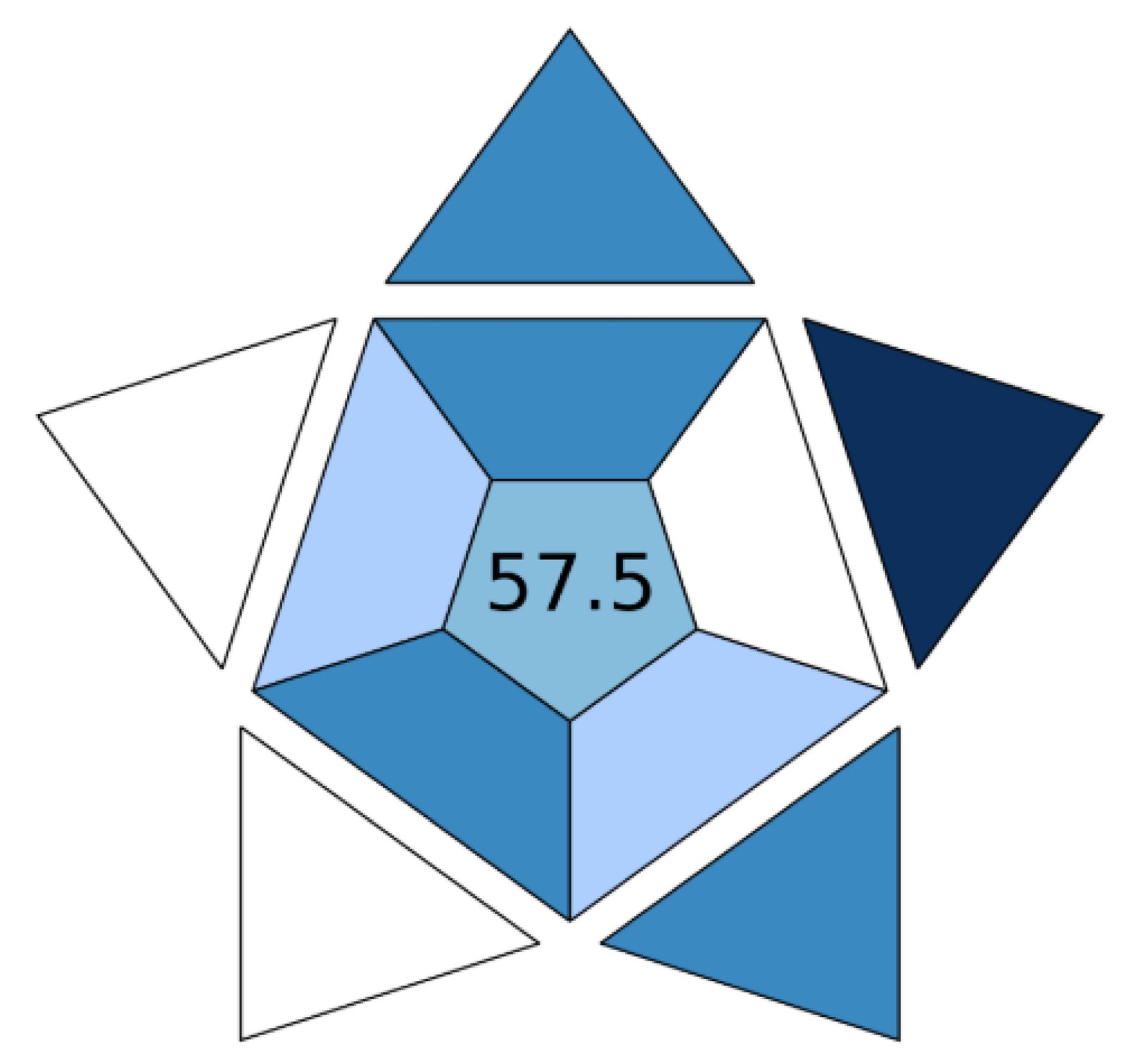	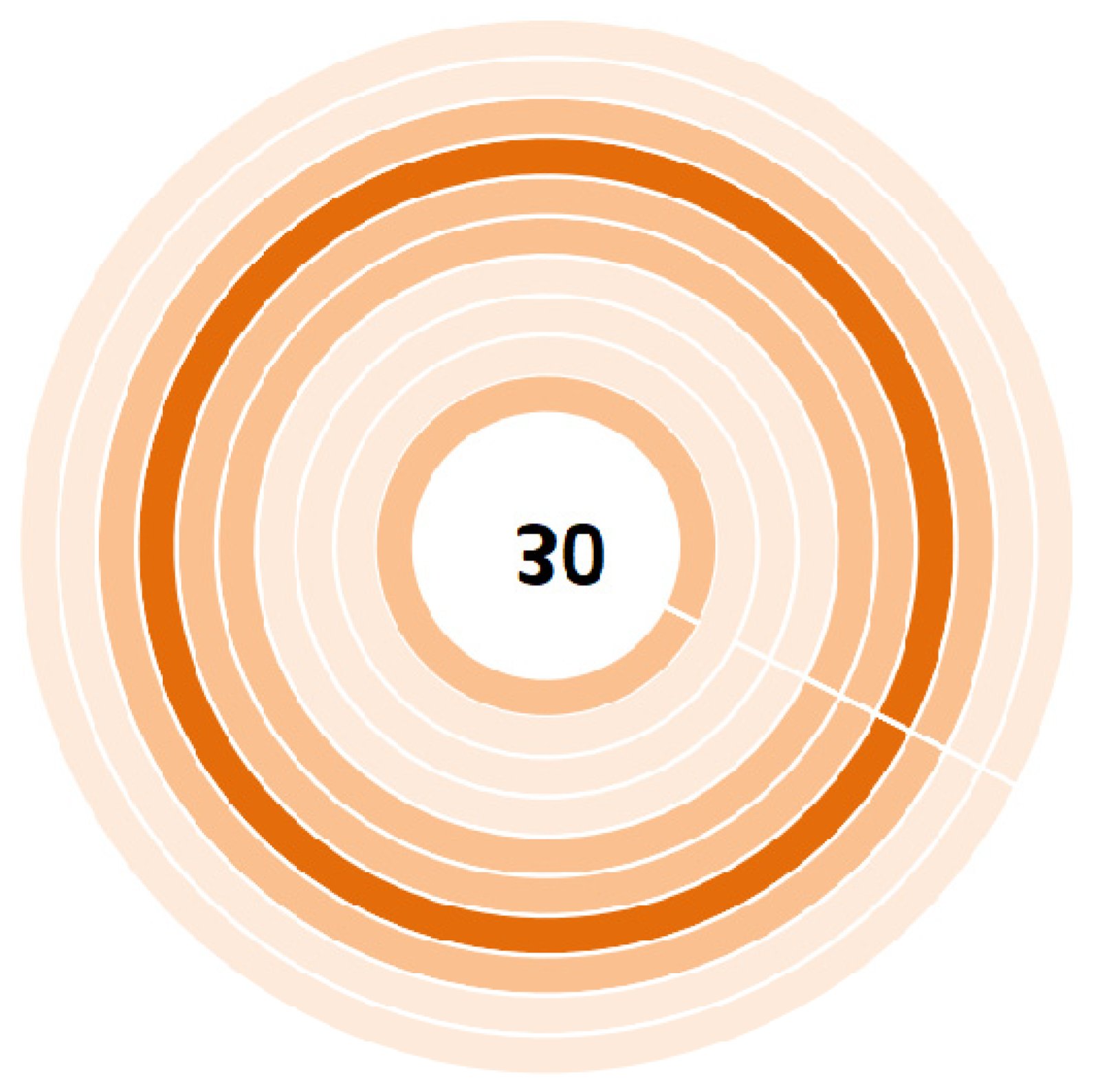	[30]
10	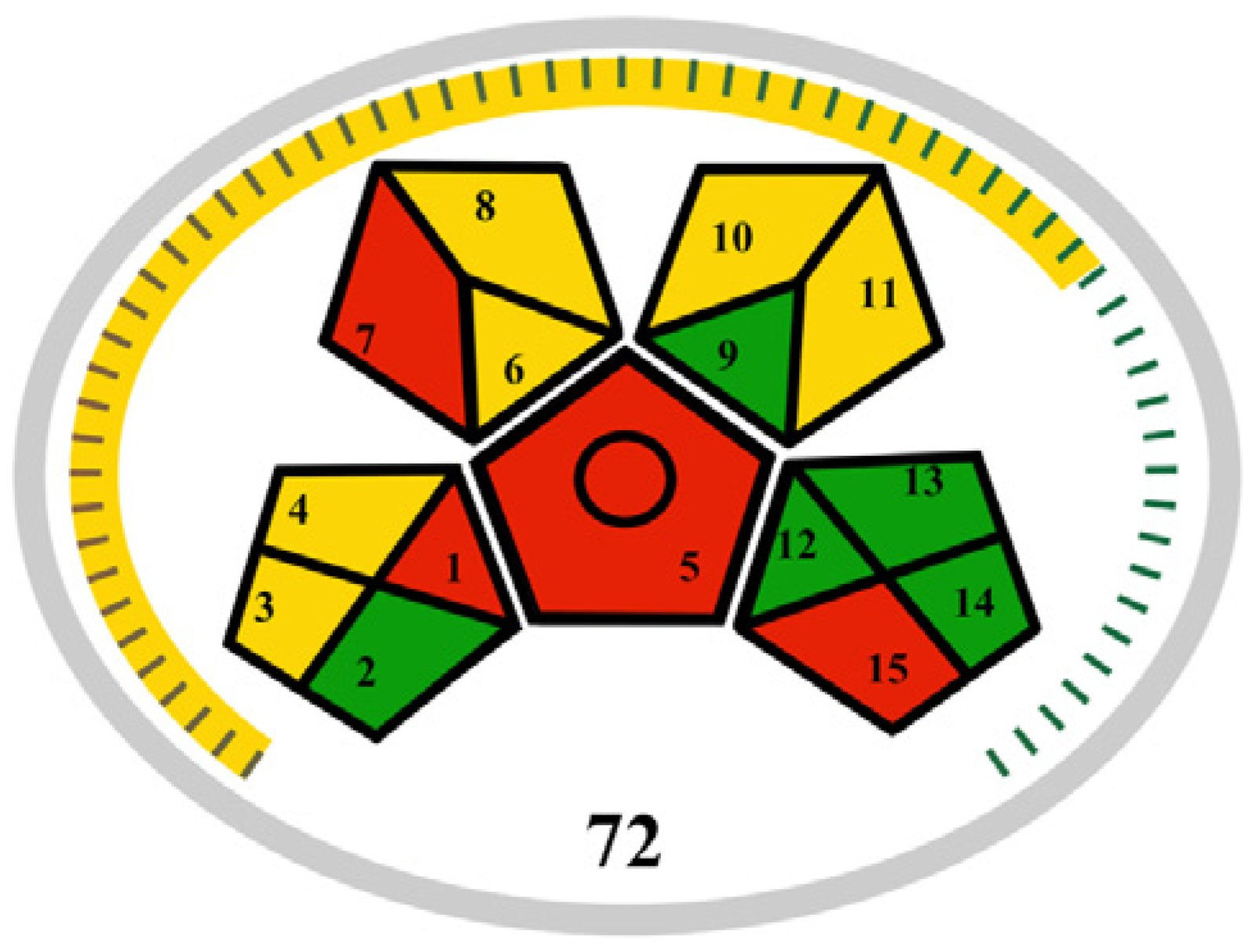	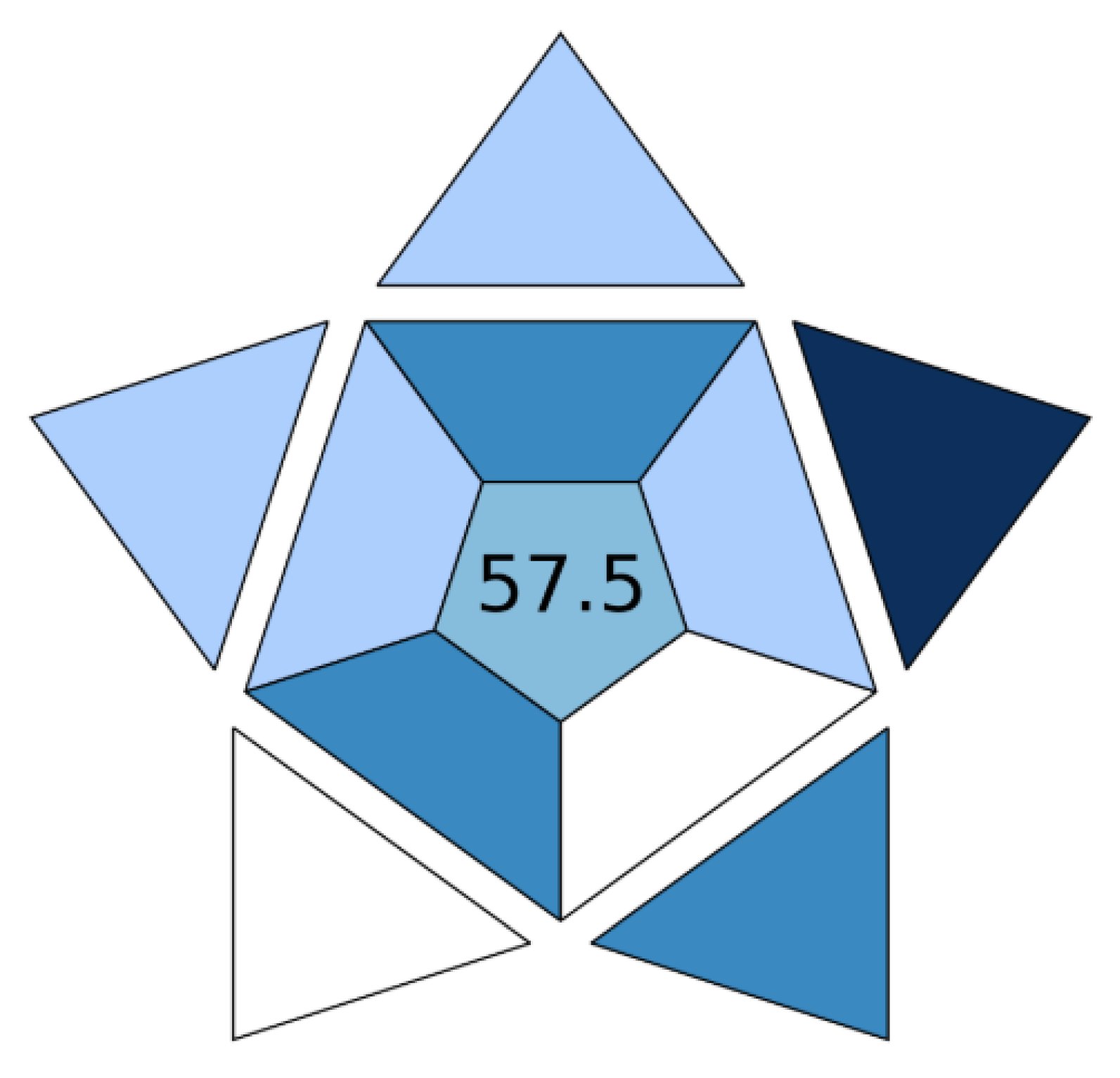	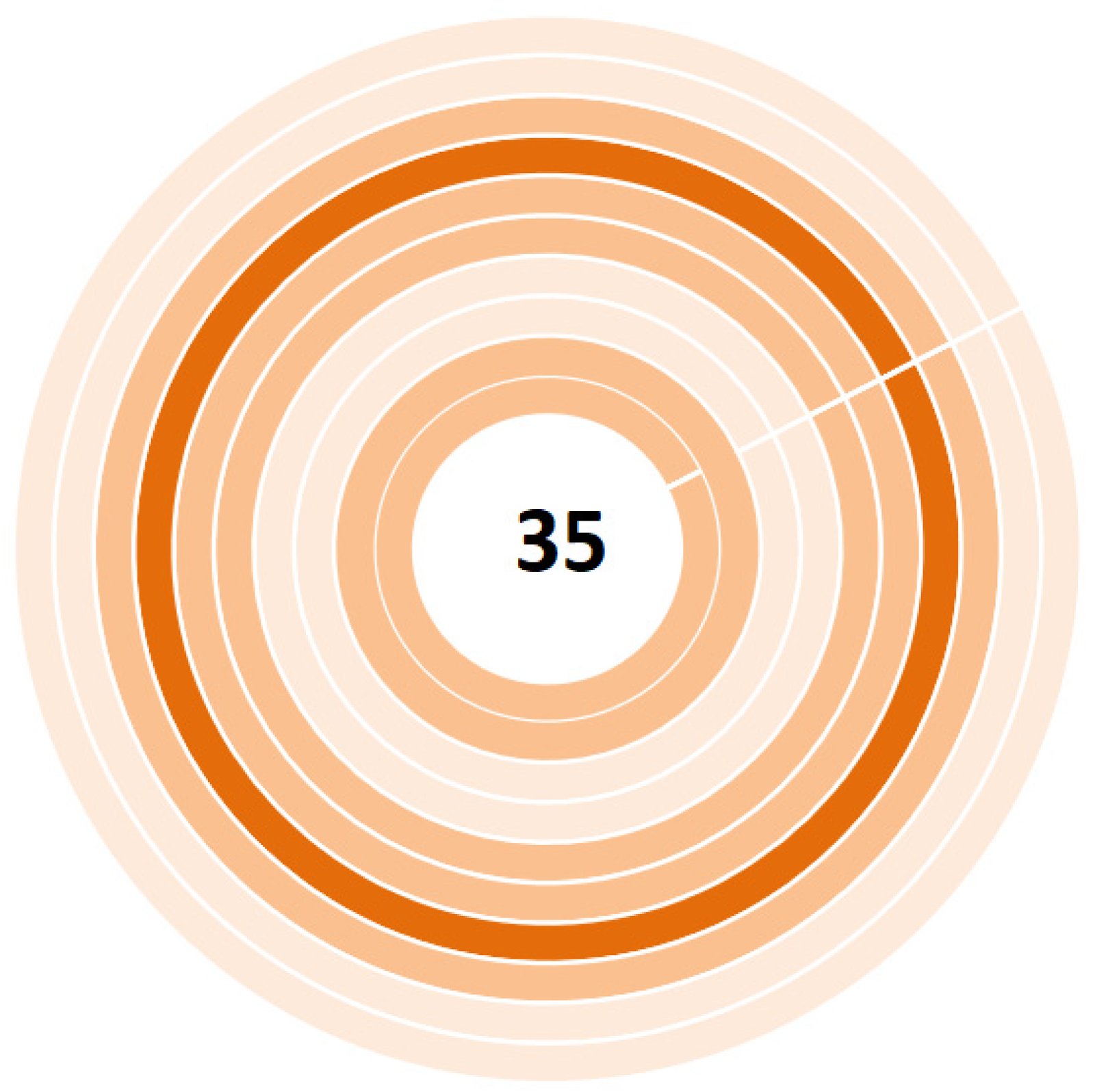	[31]
11	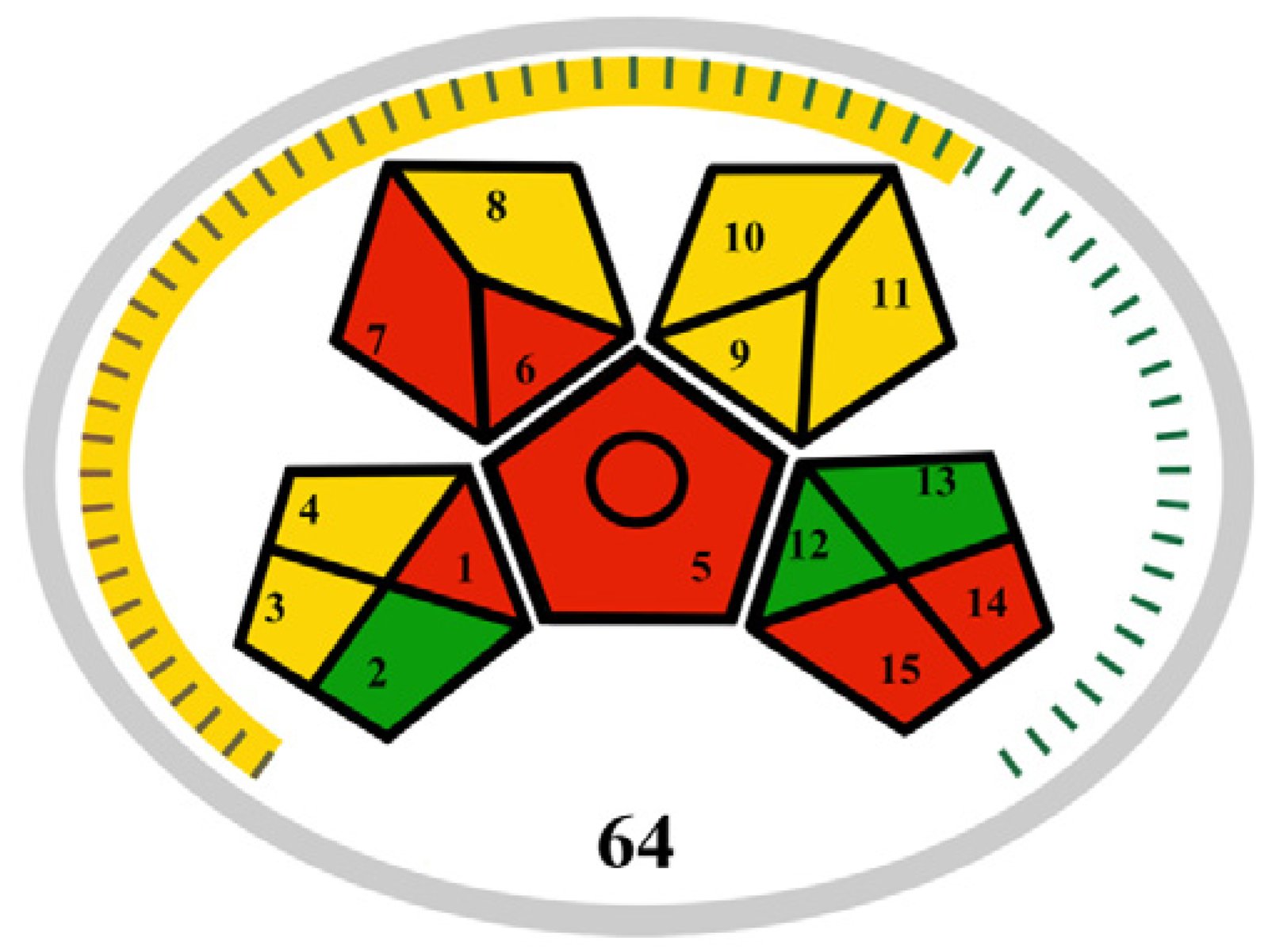	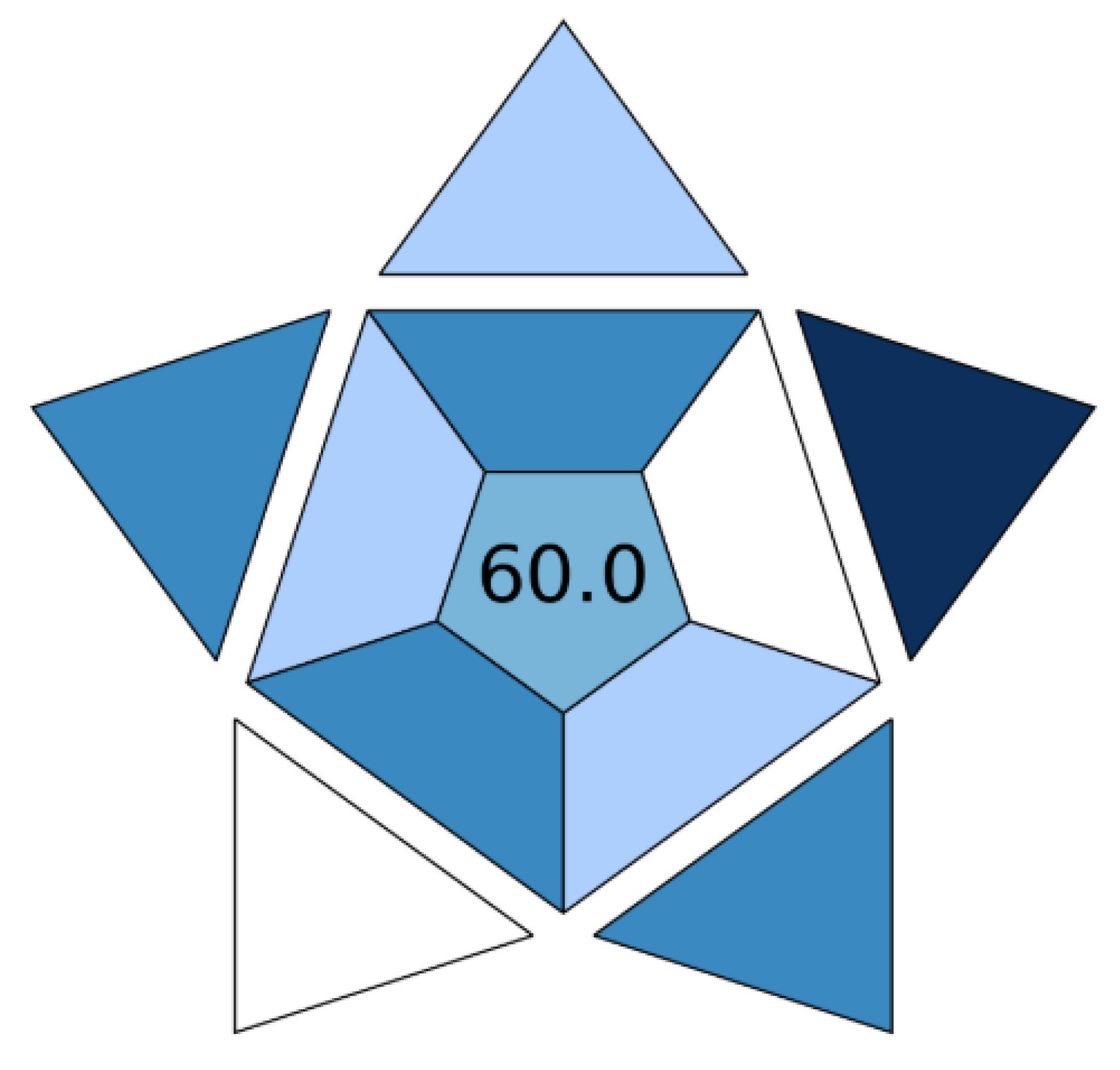	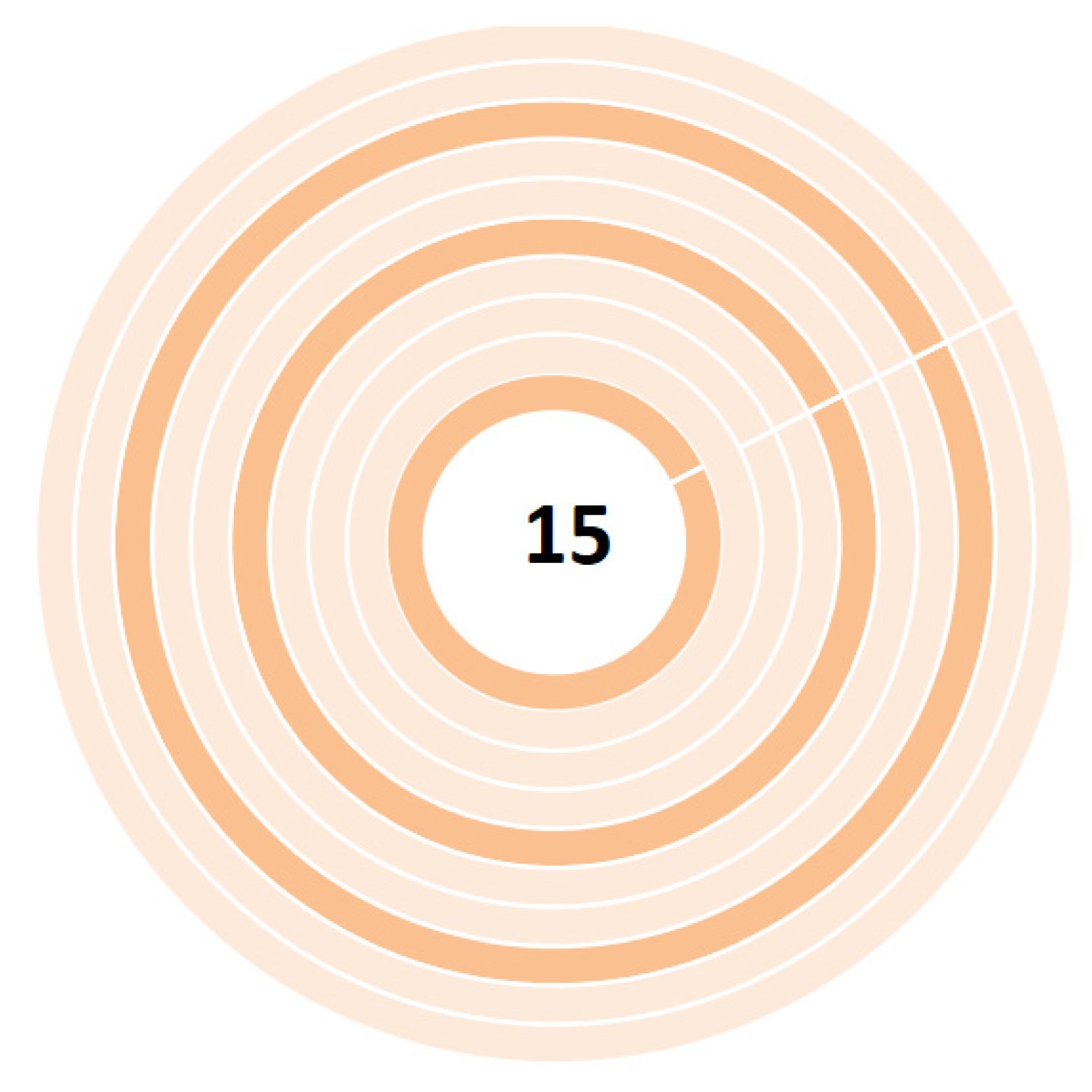	[32]
12	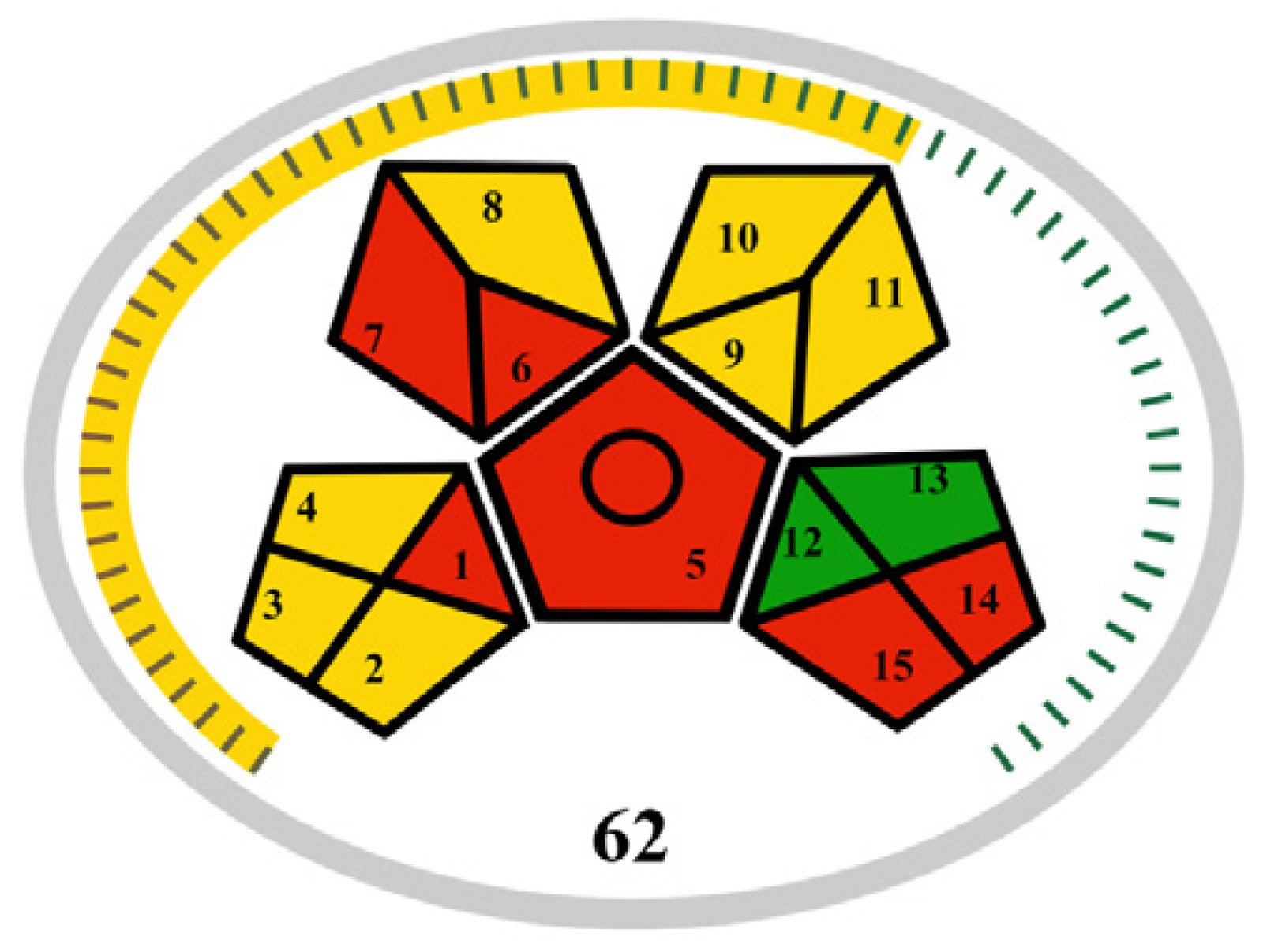	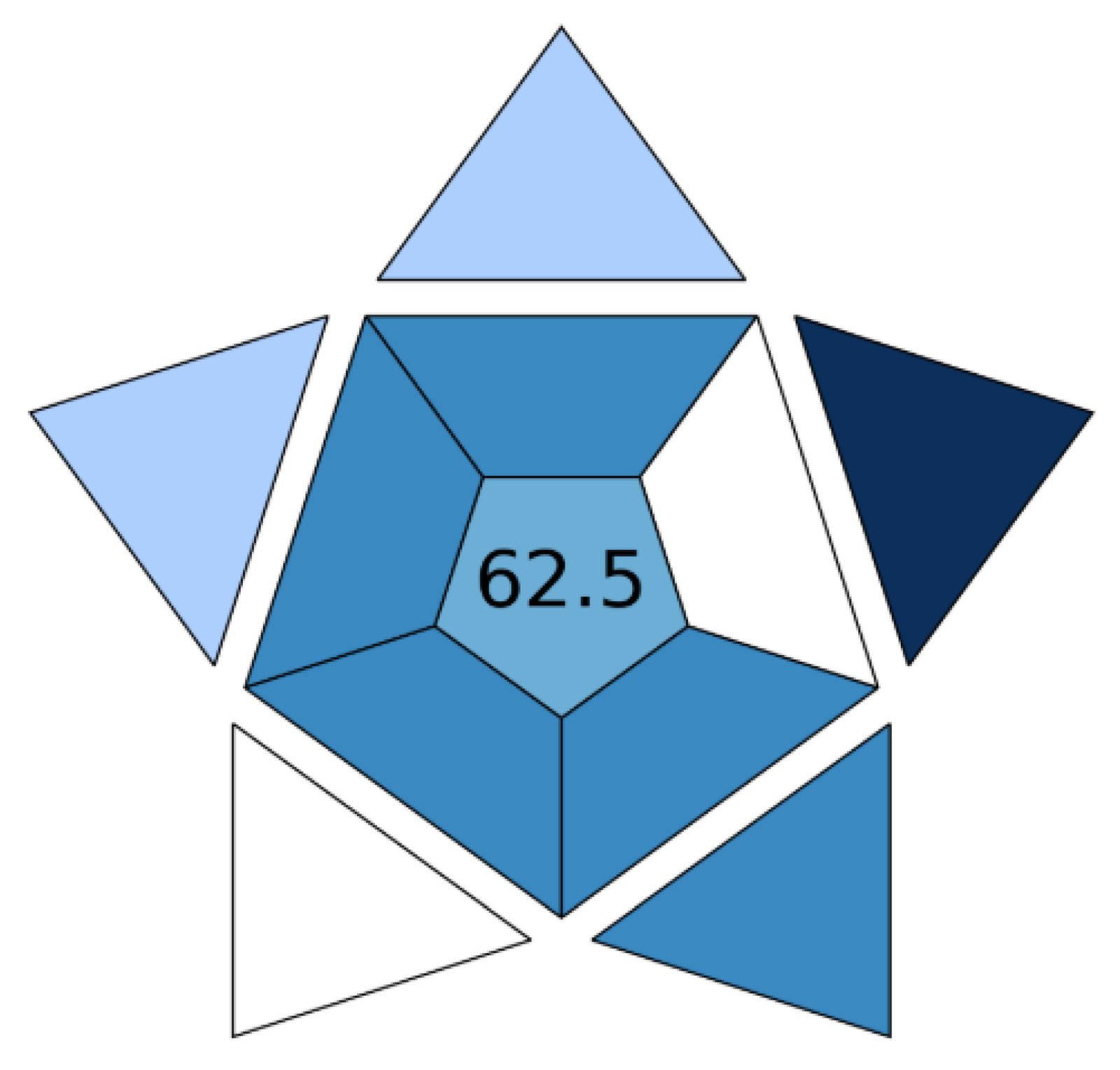	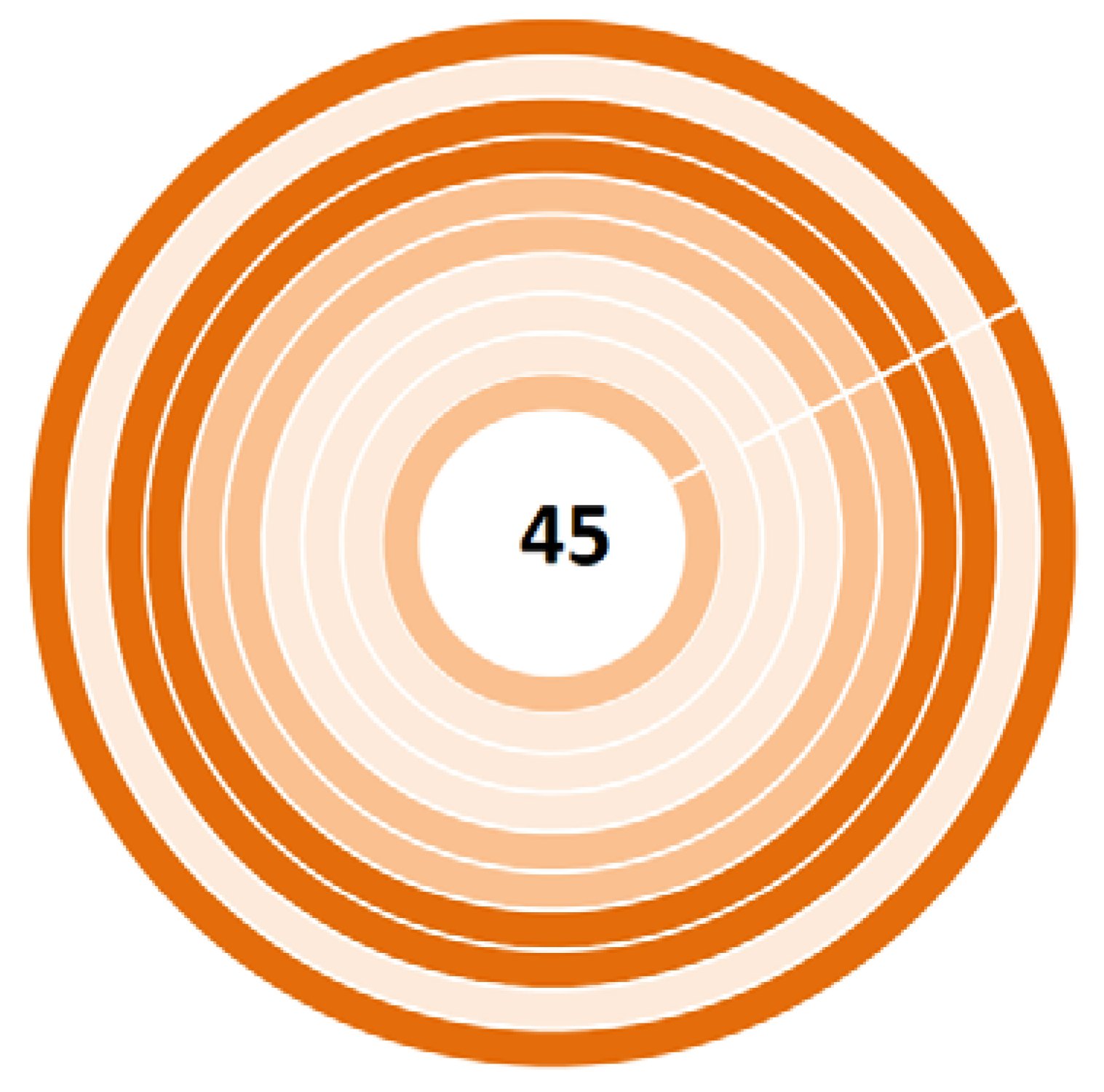	[33]
13	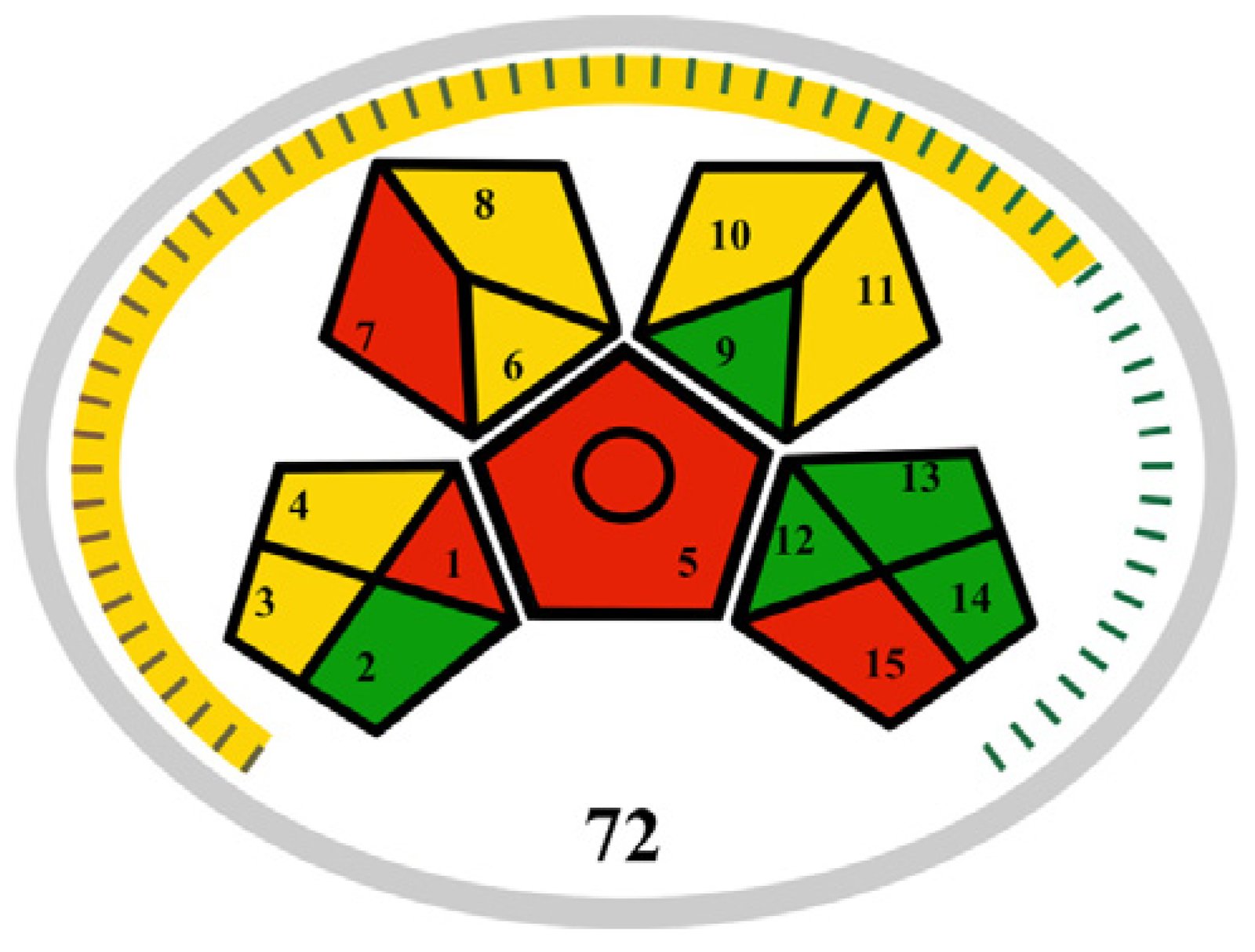	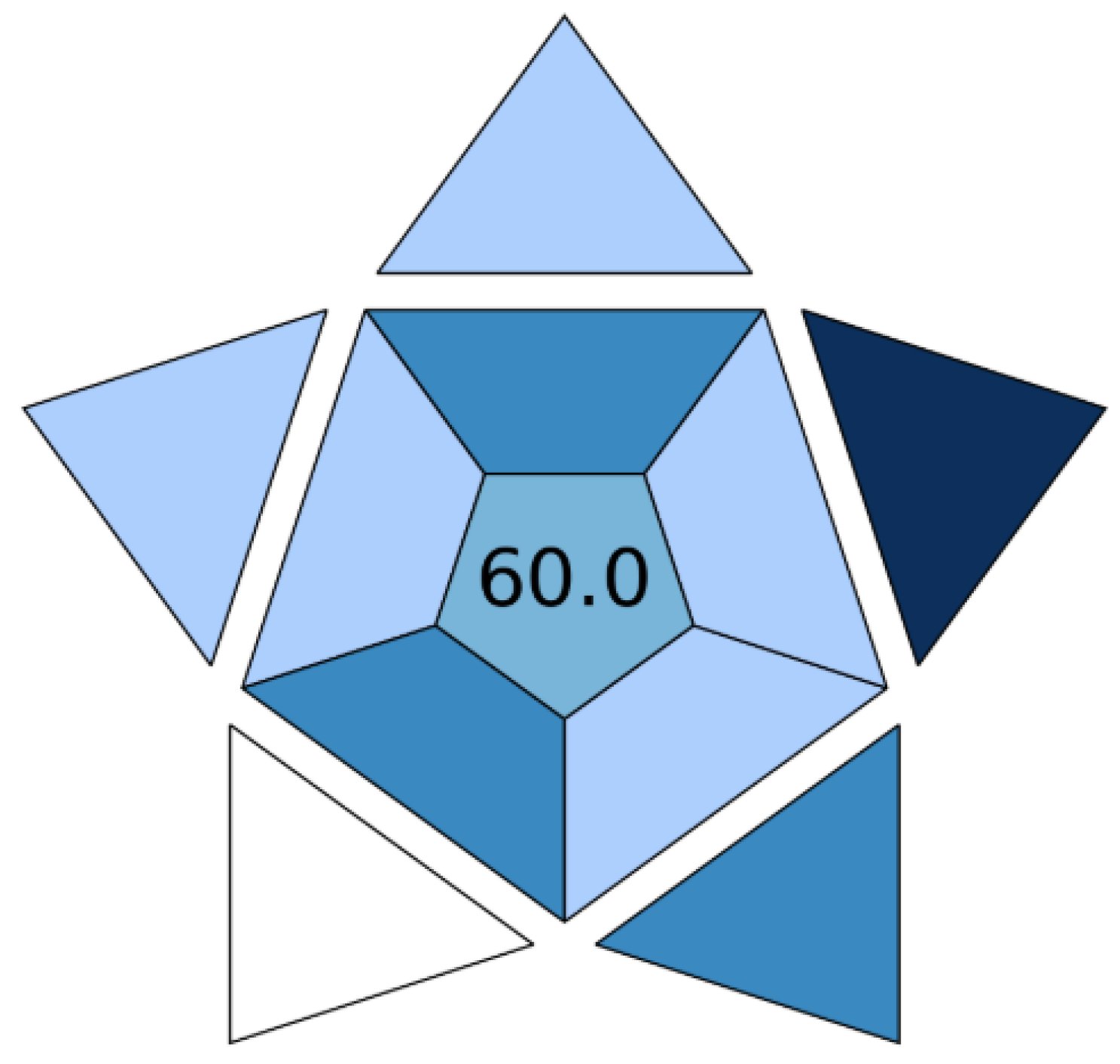	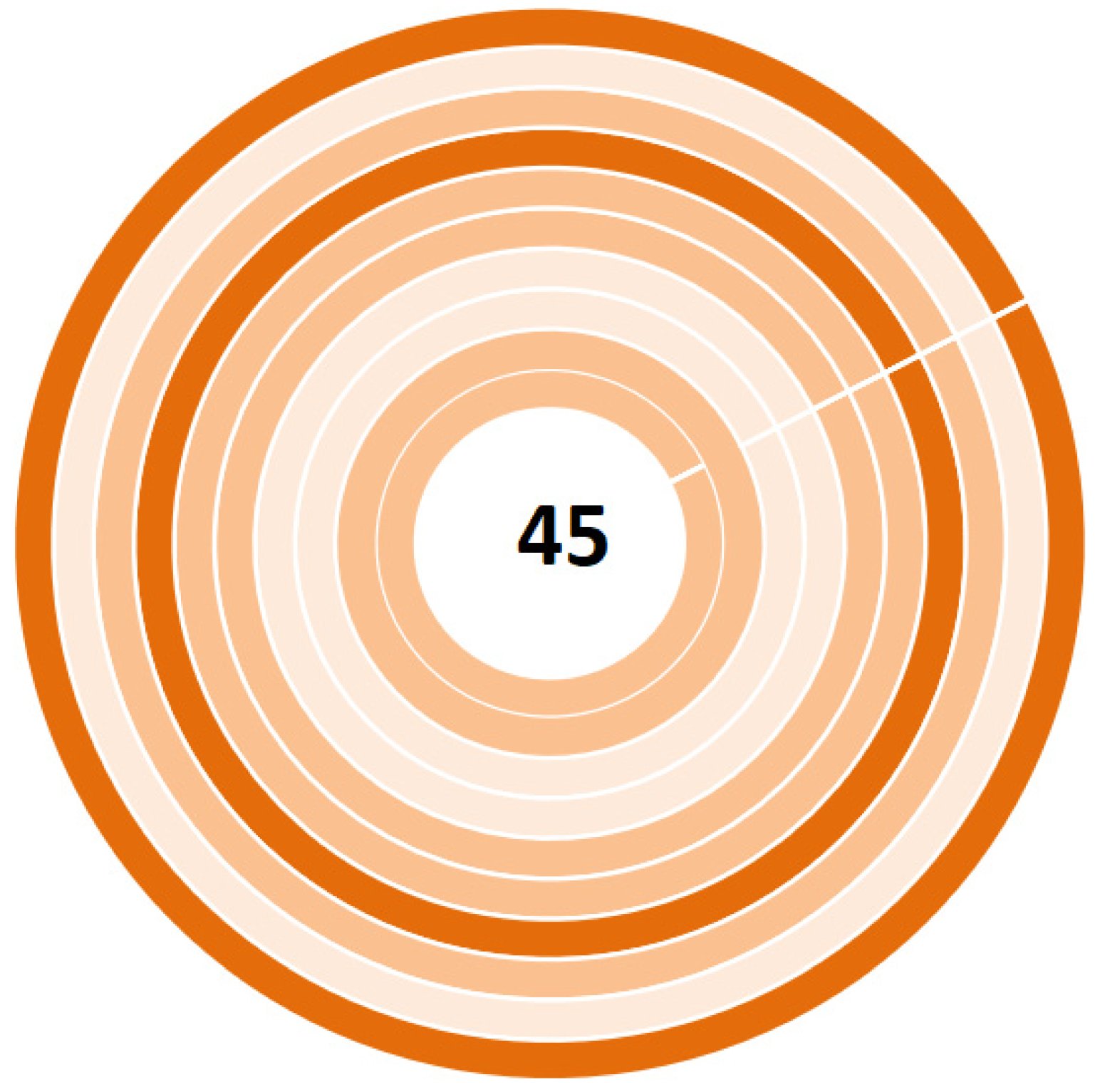	[34]
14	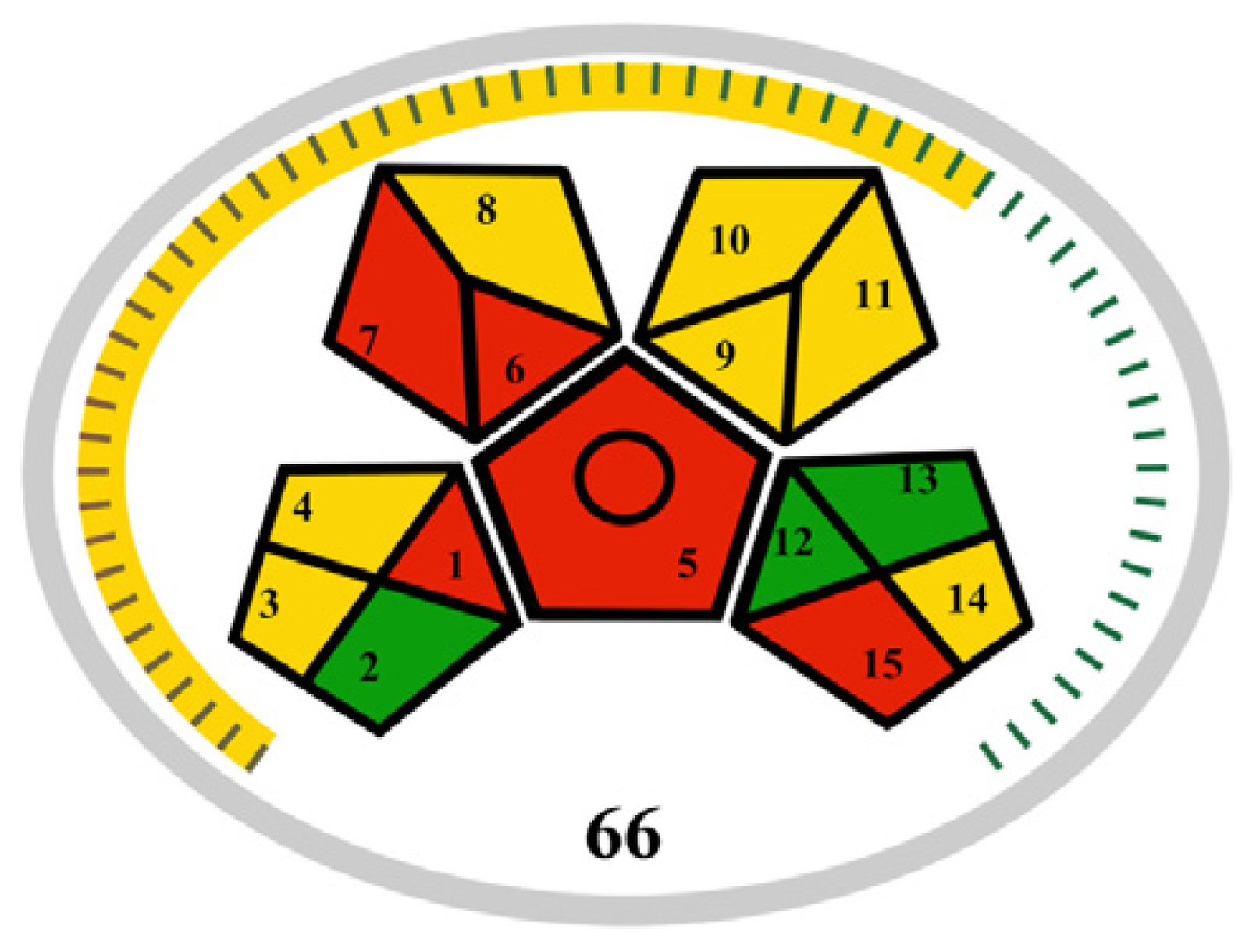	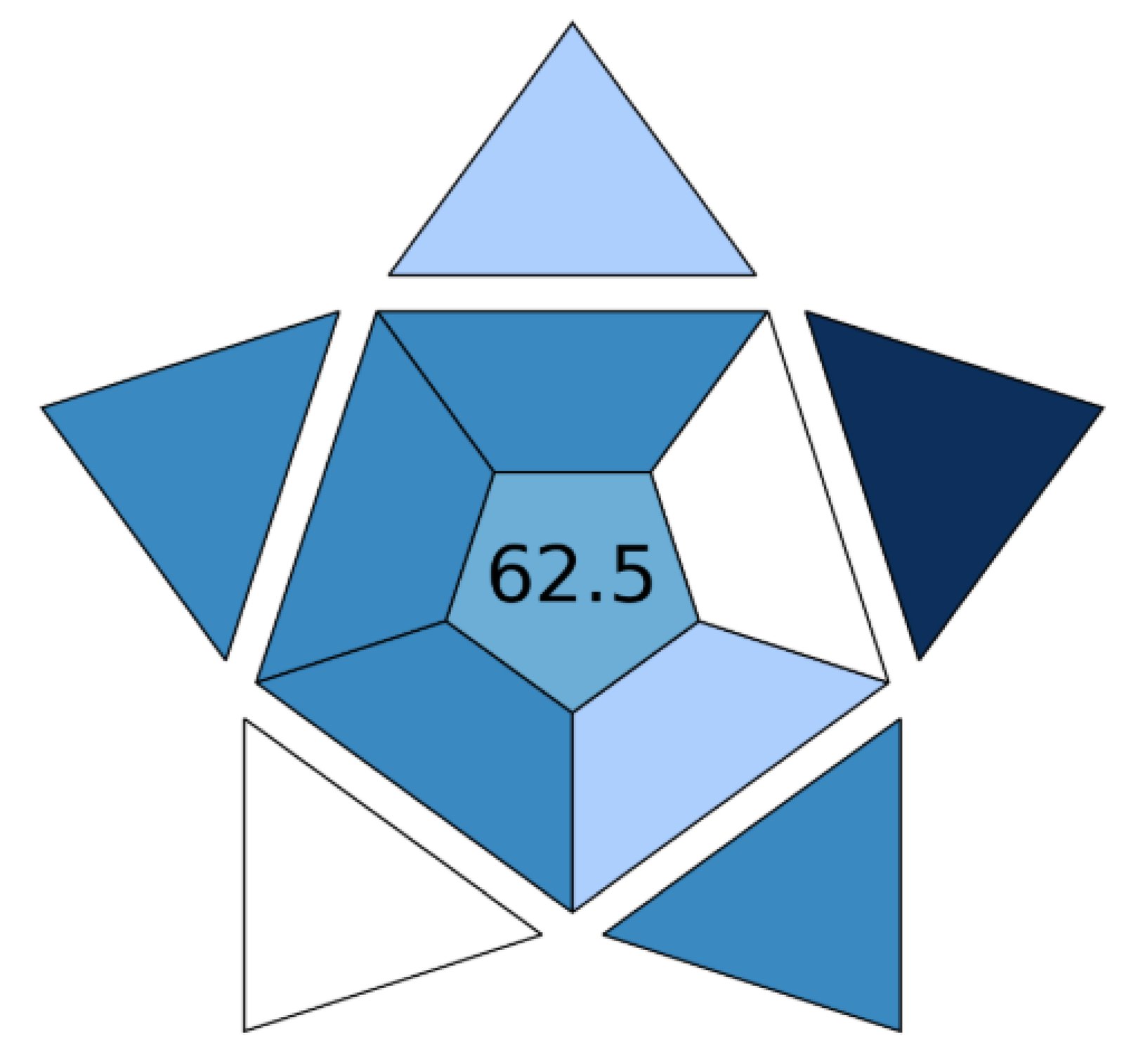	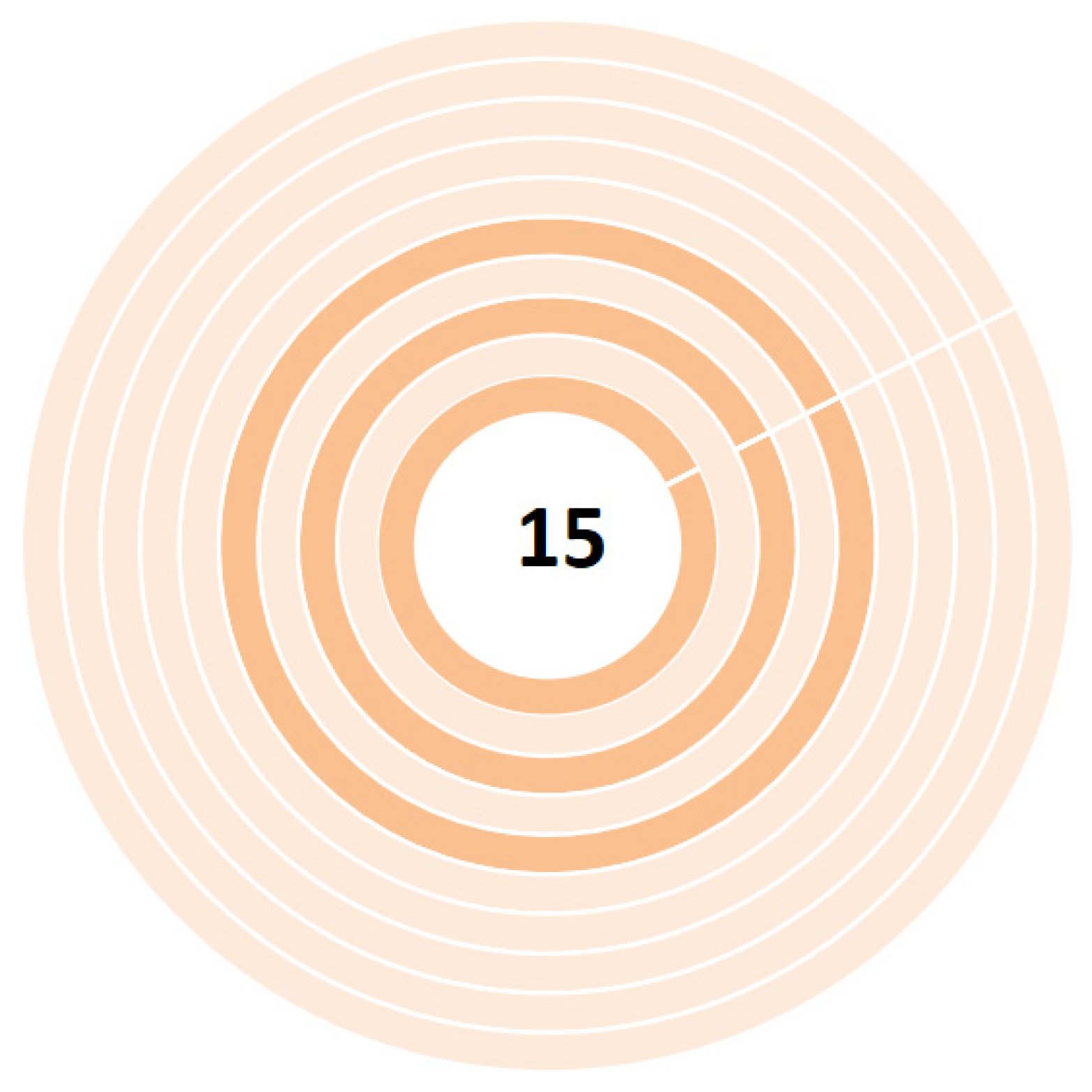	[35]
15	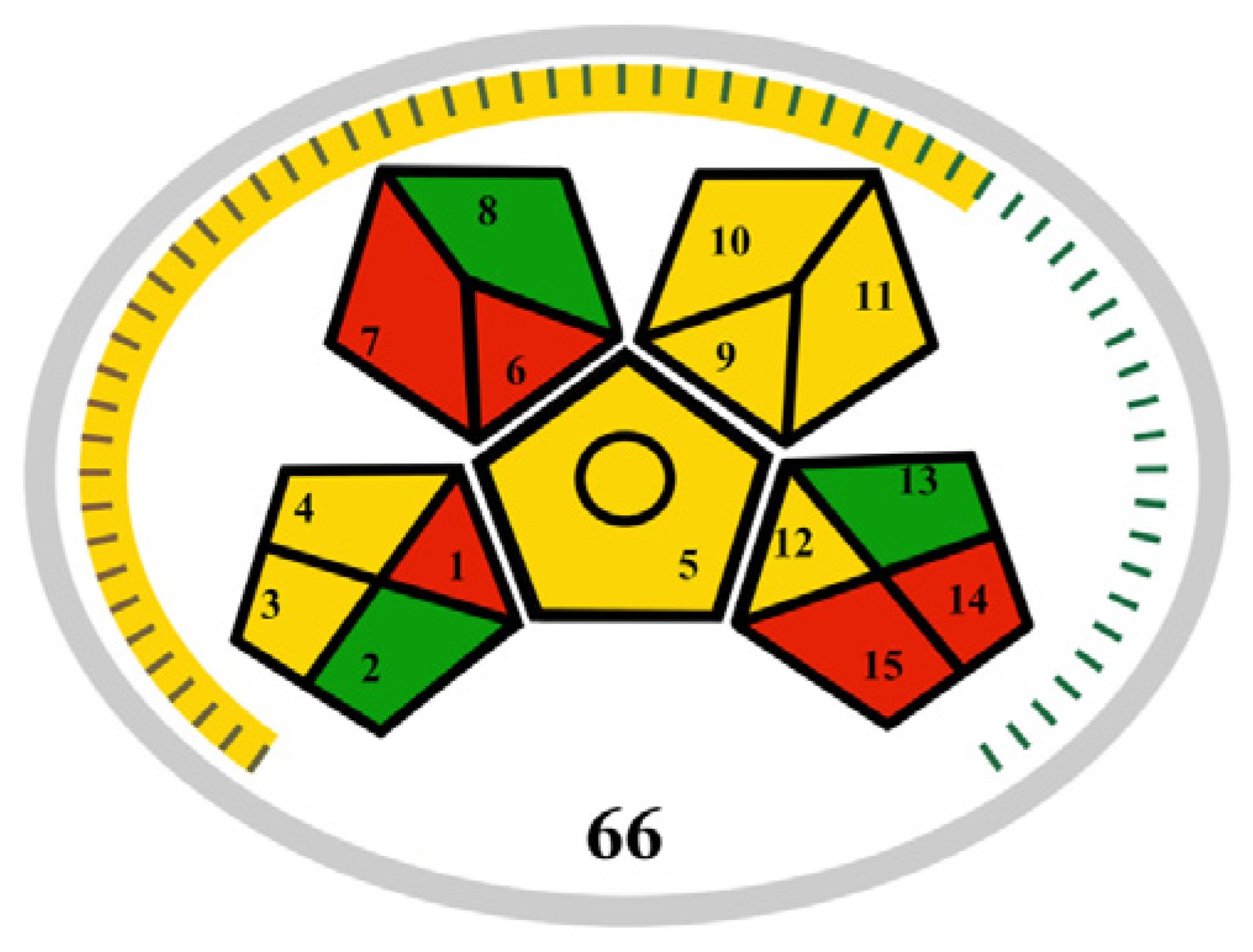	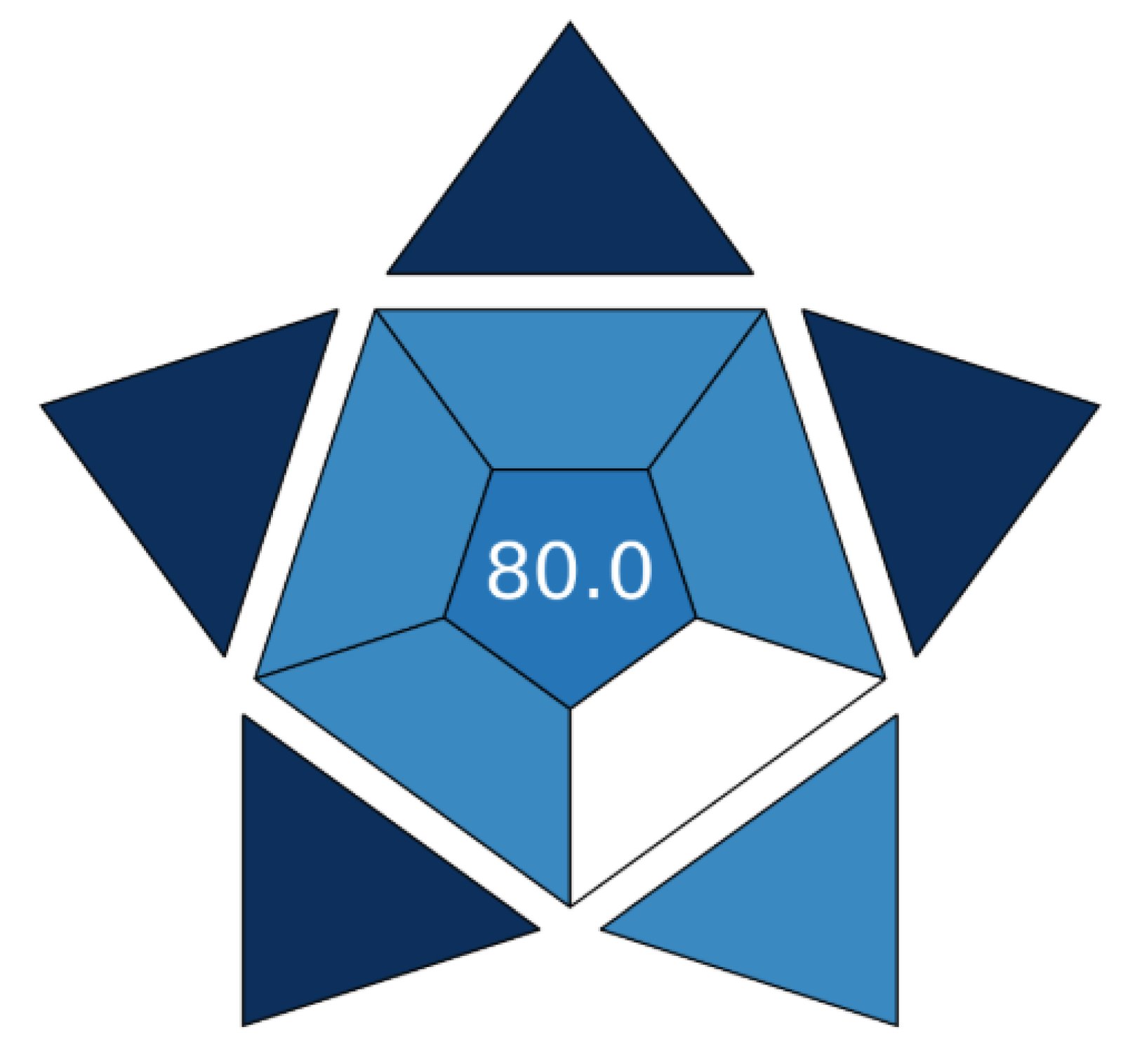	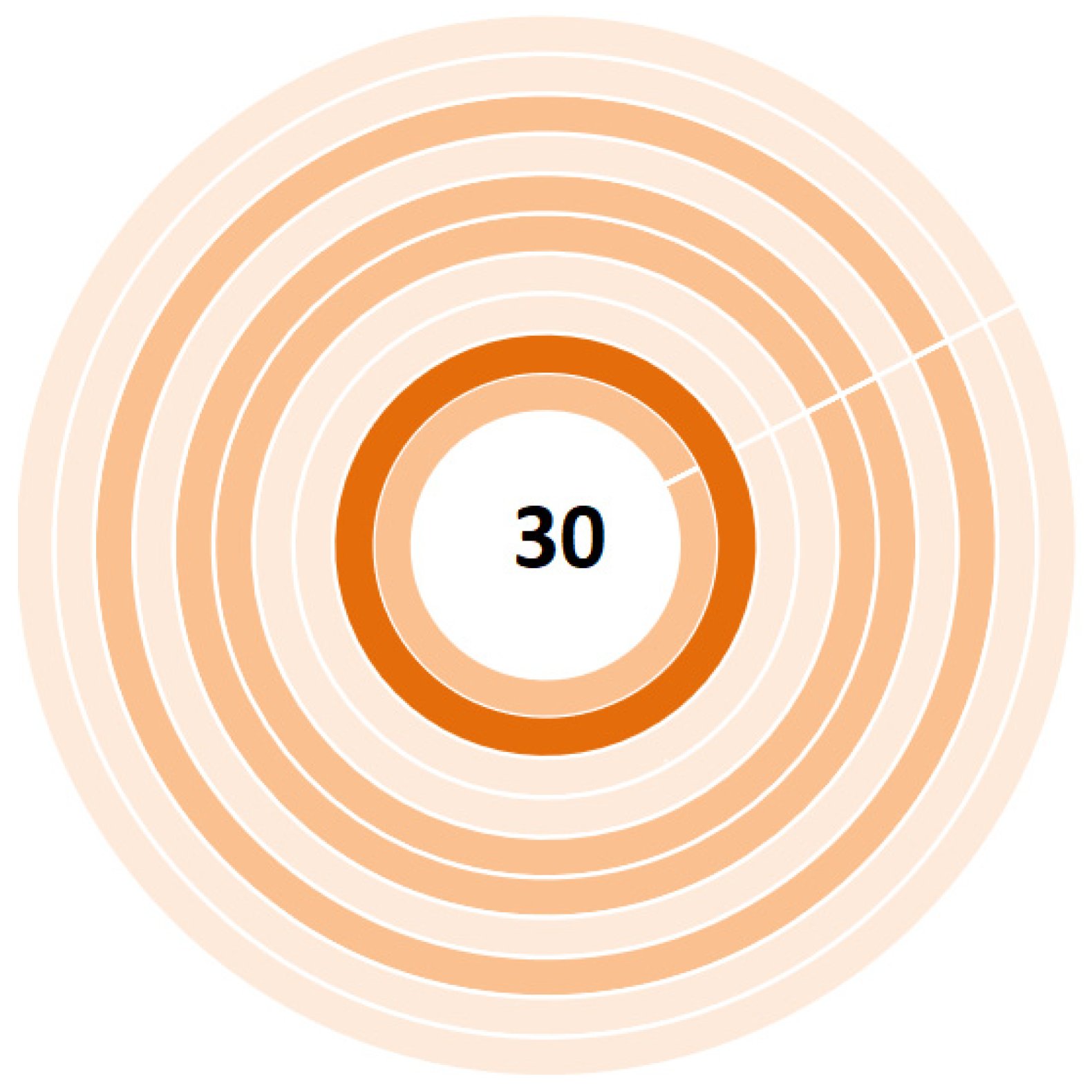	[36]
16	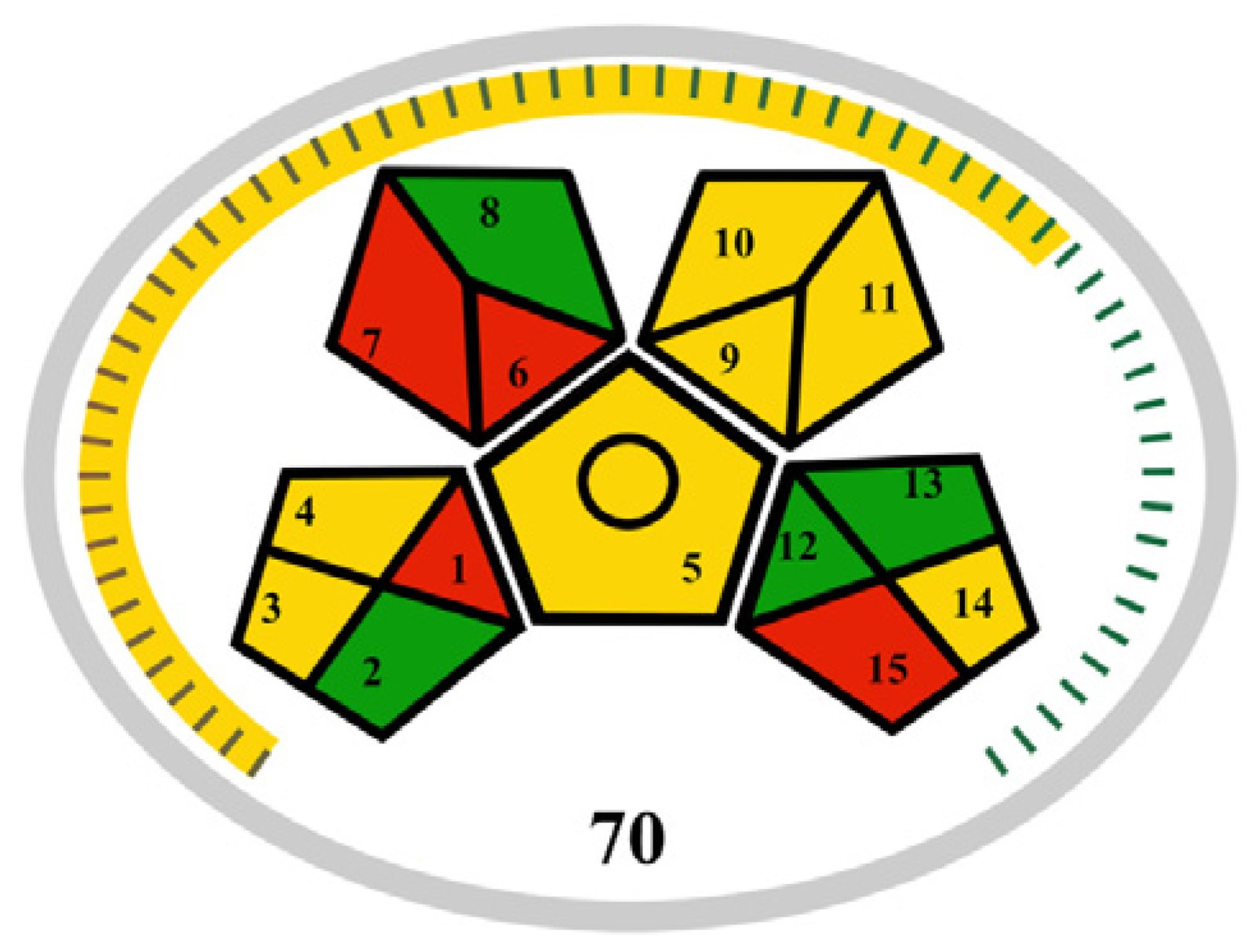	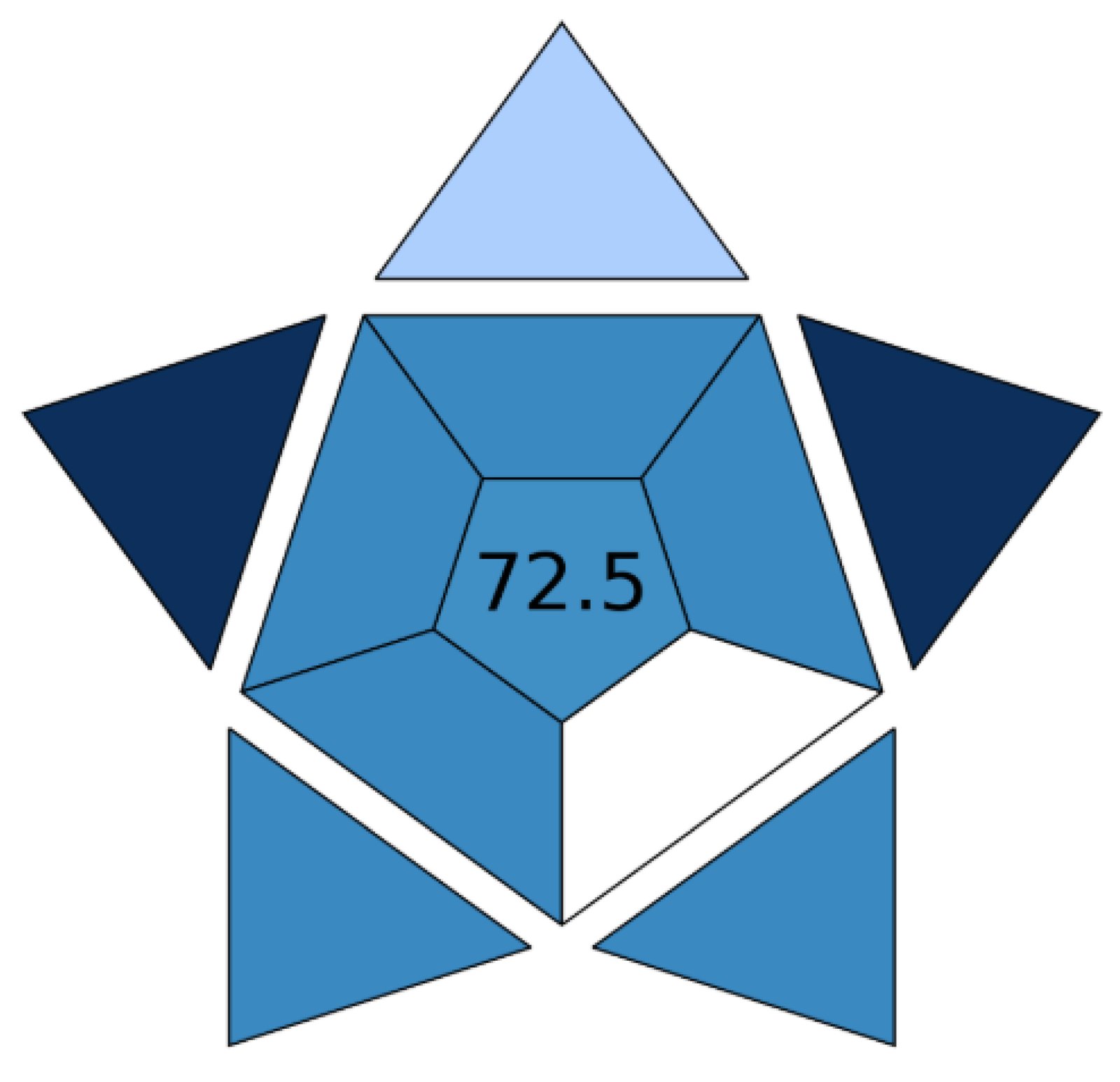	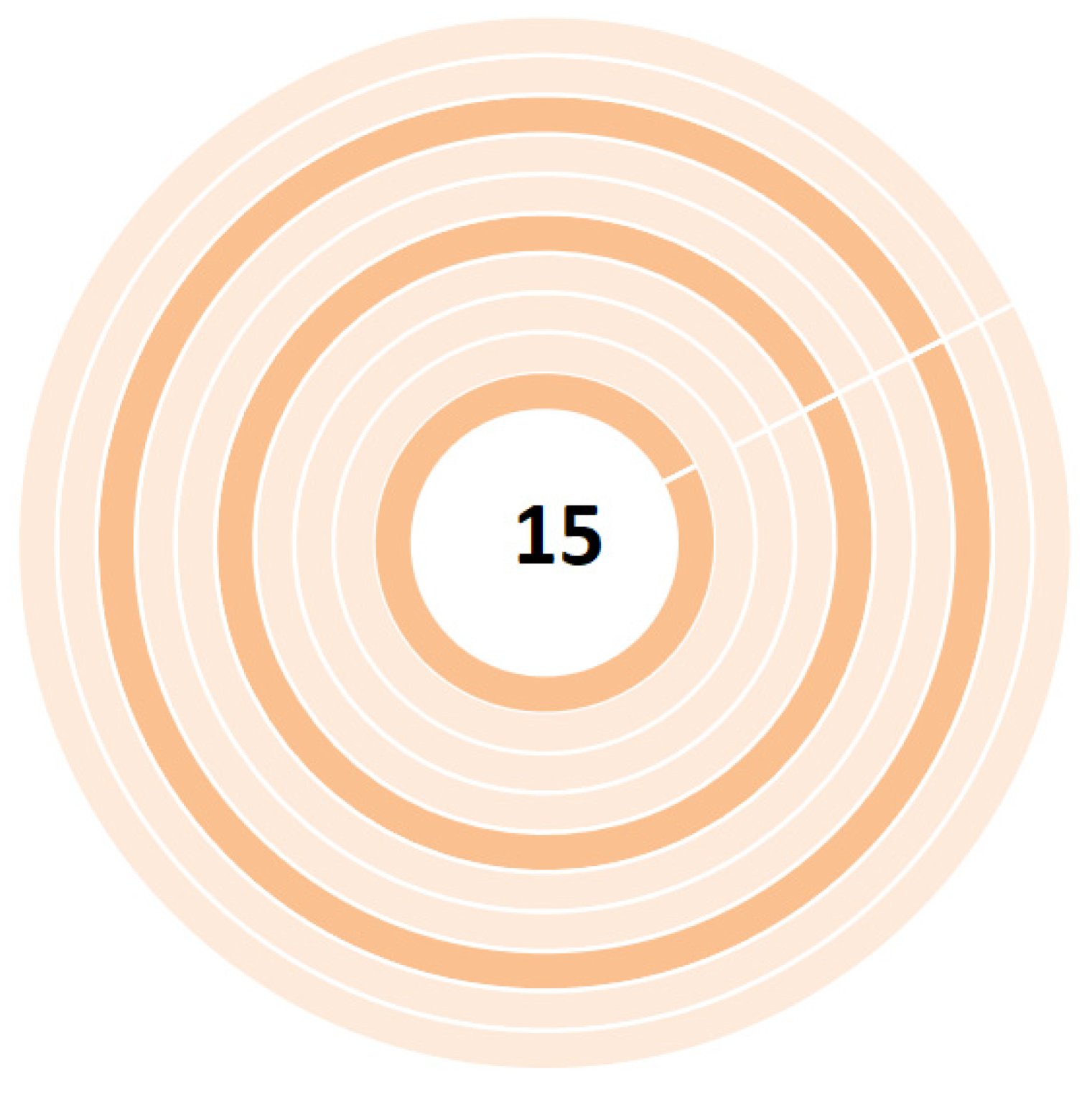	[37]

The different shapes, colors, and numbers represent the results generated by the respective software-based assessment tools. Each visual element corresponds to the specific assessment criteria and scoring outputs of the tool used.

**Table 3:** Spearman rank correlations among MoGAPI, BAGI, and IMOGI scores across 16 analytical methods.

Pair	Spearman’s $\rho_{s}$	*p*-Value
MoGAPI–BAGI	−0.245	0.361
MoGAPI–IMOGI	0.408	0.116
BAGI–IMOGI	−0.406	0.119

## Data Availability

The data presented in this study are contained within the article.

## Supplementary Materials

The following supporting information can be downloaded at: https://www.mdpi.com/article/10.3390/molecules31213828/s1, Table S1. Characteristics of the selected analytical methods for paraben determination; Tables S2. Detailed criterion-level assessment of the selected methods using MoGAPI; Table S3. Detailed criterion-level assessment of the selected methods using BAGI; Table S4. Detailed criterion-level assessment of the selected methods using IMOGI; Table S5. Database-specific search strategies.
