# Supplementary material for: Multi-Dimensional Benchmarking of Liquid Chromatographic Methods for Parabens Determination in Cosmetic Products: Toward Sustainable Analysis
Source: Molecules. 2026 Oct 23;31(21):3828. doi: 10.3390/molecules31213828 (PMC13765347; doi:10.3390/molecules31213828)
Supplement: Supplementary file 1 [file molecules-31-03828-s001.zip › molecules-4615592-supplementary.pdf]

Supplementary Materials

Multi-Dimensional Benchmarking of Liquid Chromatographic Methods for Parabens Determination in Cosmetic Products: Toward Sustainable Analysis

Table S1. Characteristics of the selected analytical methods for paraben determination.

No.	LOQ	Recovery	Precision (RSD)	Linearity range	R^2	Chromatographic conditions
M1	0.01% to 0.04 % (w/w)	14.25% -13.85%*	<10.47%	0.1–0.8 (% w/w)	0.9930-0.9951	The mobile phase was composed of water: acetonitrile (70:30, v/v), flow rate of 1.0 mL min^{-1}, temperature of 40 ◦C, wavelength of 254 nm were used
M2	0.175-5.815 µg mL^{-1}	66% - 87%	2.6- 25.6%	1–30 µg mL^{-1}	0.9794-0.9988	The mobile phase was composed of 0.01% TFA Milli Q (A): acetonitrile (B), flow rate of 1.0 mL min^{-1} . The gradient program was as follows: 0 min 30.0% B; 10 min: 35% B; 25 min 95% B; 30 min 95% B; 30.1 min 30% B; and 40 min 30% B.
M3	NR	80% - 95%	2.3% - 4.5%	0.5-600 ng mL^{-1}	> 0.9992	The mobile phase was composed of H2O (A) and methanol (B) with a flow rate of 0.8 mL min^{-1}. The gradient elution program was: 60% A for 5 min, then decreased to 40% in 10 min and held for 3 min, and finally returned to 60% A in 2 min. The detection wavelength was 260 nm.
M4	4.6- 4.6 µg L^{-1}	NR	0.7- 2.41%	0.01–25 µg mL^{-1}	0.9994-0.9999	The mobile phase was composed of deionized water: acetonitrile (60:40, v/v), flow rate of 1.0 mL min^{-1}
M5	0.216- 0.451 mg L^{-1}	88.5% -101%	<5.6%	0.5–100 mg L^{-1}	0.9980-0.9997	The analytes were separated using isocratic elution with a 70:30 (v/v) mixed solution of methanol and water at a flow rate of 0.8 mL/min. The detection wavelength was set at 254 nm.
M6	NR	97.24-100.9%	≤ 0.98	10.9-229.8 µg mL^{-1}	≥ 0.9998	The mobile phase was composed of methanol: THF:ACN:water (10:5:25:60, %, v/v). The flow rate was 1.0 mL/min. The detection wavelength was set at 280 nm.
M7	0.51–0.99 ng mL^{-1}	82.19 -102.45%	0.43-3.33	20–4000 ng mL^{-1}	0.9992-0.9997	The gradient elution was utilized with a mobile phase composed of ethanol (B) and water (A) as follows: 0–8 min (5 to 100%, B), 8–10 min (5%, B), followed by 0.5 min post time for equilibration of the column. The detection wavelength was set at 254 nm.
M8	5.20- 69.4 µg g^{-1}	86.1-118 %	≤ 14.9**	0–1500 µg g^{-1}	0.9657- 0.98	The mobile phase was composed of water (A) and EtOH (B) as mobile phase, under the following gradient program: 0–4 min, 10 % B, 4–9 min, 10–30 % B, held 1 min. Then, the column has been re-equilibrated at 10 % B for a conditioning time of 2 min. The flow rate was 1.2 mL/min. The detection wavelength was set at 254 nm.
M9	0.63–1.1 µg L^{-1}	87.9 -100.8%	< 8.7%	20-1000 µg L^{-1}	0.9949-0.9988	The mobile phase was composed of MeOH and water 60:40 (v/v), the flow rate was 1.0 mL/min. The detector was set at 254 nm.
M10	0.03–0.05 µg g^{-1}	79.4% -108.6%	≤ 5.2 %	0.05–10 µg g^{-1}	≥ 0.9995	The mobile phase was composed of water and methanol with a gradient elution program as follows: 0–3 min (50% methanol), 3–10 min (50–90% methanol), 10–12 min (90% methanol), 12–15 min (90–50% methanol), followed by a 5-min post-gradient equilibration. The flow rate was 1.0 mL/min. The detector was set at 254 nm.

M11	0.0023-0.0043 µg g^{-1}	87-108%	8.5-15%	1-32 µg g-1	0.9972- 0.9985	The mobile phase was composed of acetonitrile and water in a 40:60 v/v ratio, with a flow rate of 1 mL/min.
M12	0.09–0.12 µg Kg^{-1}	91.6–100 %	≤ 2.6	0.1–750 µg Kg^{-1}	0.9916-0.9999	The mobile phase was isocratic with the mixture of tetrahydrofuran: water: methanol: acetonitrile (5:60:10: 25 v/v). The column temperature was set at 25 ◦C. The flow rate was 1.2 mL min^{-1}.
M13	0.05 g mL^{-1}	89-105 %	≤ 12 %	0.05–4 g mL^{-1}	0.9994- 0.9996	The mobile phase A and B consisted of HPLC-grade water and acetonitrile, respectively. Gradient elution program is the following: Constant 15 % B at 0–2.0 min; 15–35 % B gradient at 2.0–2.5 min; 35–70 % B gradient at 2.5–20.0 min; 70–97 % gradient B at 20.0–20.5 min; constant 97 % B at 20.5–23.0 min. The column temperature was set at 25 ◦C. The flow rate was 0.25 mL min^{-1}.
M14	0.004-0.007 µg g^{-1}	92.33%-101.43%	<6%	0.008–20 µg mL^{-1}	0.997–0.999	The mobile phase was composed of water (0.1% trifluoroacetic acid, pH 2.1, solvent A) and acetonitrile (solvent B) at a flow rate of 0.5 mL/min. The gradient mobile phase elution was 0–2 min (B, 12.5%), 2–4 min (B 20–30%), 4–16 min (B, 30–50%), 16–22 min (B, 50–100%), return to its equilibrium conditions and 22–30 min.
M15	0.2 µmol L^{-1}	88-102%	5.5-8.7%	0.2–20 µmol L^{-1}	0.998- 0.999	The mobile phase was composed of water: methanol. The flow rate was 3.6 mL min^{-1}
M16	0.10–1 µg mL^{-1}	92.0–111.9%	<3.5%	0.10–500 µg mL^{-1}	0.9988- 0.9998	The mobile phase used a 5-mmol/L ammonium formate solution (pH 4.2 adjusted by formic acid) and acetonitrile. The gradient elution was applied as follows: 0–4 min in 25% acetonitrile, 4–12.5 min in a linear gradient from 25 to 45% acetonitrile and 12.5–17 min in 45% acetonitrile. The flow rate was 1.5 mL/min. The detector was set at 230 nm.

* RE: relative error; RSD: relative standard deviation; NR: not reported; **CV%;

Table S2. Detailed criterion-level assessment of the selected methods using MoGAPI.

No.	Collection	Preservation	Transport	Storage	Type of method	Scale of extraction	Solvents used	Additional treatment	Solvents amount	Health hazard	Safety hazard	Energy	Occupational hazard	Waste	Waste treatment	Quantification
M1	Offline	None	Required	Under normal condition	Extraction required	Micro-extraction	Non green solvents/ reagents used	Simple treatment	<10 mL	Moderately toxic, NFPA=2 or 3	Highest NFPA flammability or instability score of 2 or 3	≤1.5 kWh per sample	Hermetic sealing of analytical process	1-10 mL	No treatment	Yes
M2	Offline	None	Required	Under normal condition	Simple procedures	Micro-extraction	Non green solvents/ reagents used	None	10-100 mL	Moderately toxic, NFPA=2 or 3	Highest NFPA flammability or instability score of 2 or 3	≤0.1 kWh per sample	Hermetic sealing of analytical process	>10 mL	No treatment	Yes
M3	Offline	None	Required	Under normal condition	Extraction required	Macro-extraction	Non green solvents/ reagents used	Advanced treatment	<10 mL	Moderately toxic, NFPA=2 or 3	Highest NFPA flammability or instability score of 2 or 3	≤0.1 kWh per sample	Hermetic sealing of analytical process	>10 mL	No treatment	Yes
M4	Offline	None	Required	Under normal condition	Extraction required	Micro-extraction	Green solvents/ reagents used	Simple treatment	<10 mL	Moderately toxic, NFPA=2 or 3	Highest NFPA flammability or instability score of 2 or 3	≤0.1 kWh per sample	Hermetic sealing of analytical process	<1 mL	No treatment	Yes
M5	Offline	None	Required	Under normal condition	Extraction required	Micro-extraction	Non green solvents/ reagents used	Simple treatment	<10 mL	Moderately toxic, NFPA=2 or 3	Highest NFPA flammability or instability score of 2 or 3	≤0.1 kWh per sample	Hermetic sealing of analytical process	<1 mL	No treatment	Yes
M6	Offline	None	Required	Under normal condition	Extraction required	Macro-extraction	Non green solvents/ reagents used	Simple treatment	10-100 mL	Moderately toxic, NFPA=2 or 3	Highest NFPA flammability or instability score of 2 or 3	≤0.1 kWh per sample	Hermetic sealing of analytical process	>10 mL	No treatment	Yes
M7	Offline	None	Required	Under normal condition	Extraction required	Micro-extraction	Green solvents/ reagents used	Simple treatment	<10 mL	Moderately toxic, NFPA=2 or 3	Highest NFPA flammability or instability score of 2 or 3	≤0.1 kWh per sample	Hermetic sealing of analytical process	<1 mL	No treatment	Yes
M8	Offline	None	Required	Under normal condition	Extraction required	Macro-extraction	Green solvents/ reagents used	None	<10 mL	Moderately toxic, NFPA=2 or 3	Highest NFPA flammability or instability score of 2 or 3	≤0.1 kWh per sample	Hermetic sealing of analytical process	1-10 mL	No treatment	Yes
M9	Offline	None	Required	Under normal condition	Extraction required	Macro-extraction	Non green solvents/ reagents used	Simple treatment	<10 mL	Moderately toxic, NFPA=2 or 3	Highest NFPA flammability or instability score of 2 or 3	≤0.1 kWh per sample	Hermetic sealing of analytical process	1-10 mL	No treatment	Yes
M10	Offline	None	Required	Under normal condition	Extraction required	Micro-extraction	Non green solvents/ reagents used	Simple treatment	<10 mL	Moderately toxic, NFPA=2 or 3	Highest NFPA flammability or instability score of 2 or 3	≤0.1 kWh per sample	Hermetic sealing of analytical process	<1 mL	No treatment	Yes
M11	Offline	None	Required	Under normal condition	Extraction required	Macro-extraction	Non green solvents/ reagents used	Simple treatment	10-100 mL	Moderately toxic, NFPA=2 or 3	Highest NFPA flammability or instability score of 2 or 3	≤0.1 kWh per sample	Hermetic sealing of analytical process	>10 mL	No treatment	Yes
M12	Offline	Chemical or physical	Required	Under normal condition	Extraction required	Macro-extraction	Non green solvents/ reagents used	Simple treatment	10-100 mL	Moderately toxic, NFPA=2 or 3	Highest NFPA flammability or instability score of 2 or 3	≤0.1 kWh per sample	Hermetic sealing of analytical process	>10 mL	No treatment	Yes

M13	Offline	None	Required	Under normal condition	Extraction required	Micro-extraction	Non green solvents/ reagents used	Simple treatment	<10 mL	Moderately toxic, NFPA=2 or 3	Highest NFPA flammability or instability score of 2 or 3	≤0.1 kWh per sample	Hermetic sealing of analytical process	<1 mL	No treatment	Yes
M14	Offline	Chemical or physical	Required	Under normal condition	Extraction required	Macro-extraction	Non green solvents/ reagents used	Simple treatment	10-100 mL	Moderately toxic, NFPA=2 or 3	Highest NFPA flammability or instability score of 2 or 3	≤0.1 kWh per sample	Hermetic sealing of analytical process	1-10 mL	No treatment	Yes
M15	Offline	None	Required	Under normal condition	Simple procedures	Macro-extraction	Non green solvents/ reagents used	None	10-100 mL	Moderately toxic, NFPA=2 or 3	Highest NFPA flammability or instability score of 2 or 3	≤1.5 kWh per sample	Hermetic sealing of analytical process	>10 mL	No treatment	Yes
M16	Offline	None	Required	Under normal condition	Extraction required	Macro-extraction	Non green solvents/ reagents used	None	10-100 mL	Moderately toxic, NFPA=2 or 3	Highest NFPA flammability or instability score of 2 or 3	≤0.1 kWh per sample	Hermetic sealing of analytical process	1-10 mL	No treatment	Yes

Table S3. Detailed criterion-level assessment of the selected methods using BAGI.

No.	Criterion 1	Criterion 2	Criterion 3	Criterion 4	Criterion 5	Criterion 6	Criterion 7	Criterion 8	Criterion 9	Criterion 10
	Type of analysis	Multi- or single-element analysis	Analytical technique	Simultaneous sample preparation	Sample preparation	Samples per h	Reagents and materials	Preconcentration	Degree of automation	Amount of sample
M1	Quantitative	Multi-element analysis for 2-5 compounds of the same chemical class	Simple instrumentation available in most labs	2-12	Miniaturized extraction sample preparation	2-4	Common commercially available reagents	Preconcentration required after complicated stages	Semi-automated with common devices (e.g. HPLC autosampler)	51-100 mL food/environmental
M2	Quantitative	Multi-element analysis for 6-15 compounds of the same chemical class	Simple instrumentation available in most labs	2-12	Multi-step sample preparation required (e.g., LLE, SPE, and/or derivatization)	2-4	Common commercially available reagents	Preconcentration required after complicated stages	Semi-automated with common devices (e.g. HPLC autosampler)	10.1-50 mL food/environmental
M3	Quantitative	Multi-element analysis for 6-15 compounds of the same chemical class	Simple instrumentation available in most labs	1	Multi-step sample preparation required (e.g., LLE, SPE, and/or derivatization)	2-4	Need to be synthesized in the lab with advanced equipment or know how	Preconcentration required after complicated stages	Semi-automated with common devices (e.g. HPLC autosampler)	<10 mL food/environmental
M4	Quantitative	Multi-element analysis for 2-5 compounds of the same chemical class	Simple instrumentation available in most labs	1	Miniaturized extraction sample preparation	2-4	Need to be synthesized in the lab with common instrumentation and in simple way	Preconcentration required after complicated stages	Semi-automated with common devices (e.g. HPLC autosampler)	<10 mL food/environmental
M5	Quantitative	Multi-element analysis for 2-5 compounds of the same chemical class	Simple instrumentation available in most labs	1	Miniaturized extraction sample preparation	5-10	Need to be synthesized in the lab with advanced equipment or know how	Preconcentration required after complicated stages	Semi-automated with common devices (e.g. HPLC autosampler)	<10 mL food/environmental
M6	Quantitative	Multi-element analysis for 2-5 compounds of the same chemical class	Simple instrumentation available in most labs	1	Multi-step sample preparation required	5-10	Common commercially available reagents	Preconcentration required after complicated stages	Semi-automated with common devices (e.g. HPLC autosampler)	>10 mL food/environmental
M7	Quantitative	Multi-element analysis for 2-5 compounds of the same chemical class	Simple instrumentation available in most labs	1	Miniaturized extraction sample preparation	5-10	Need to be synthesized in the lab with common instrumentation and in simple way	Preconcentration required after complicated stages	Semi-automated with common devices (e.g. HPLC autosampler)	<10 mL food/environmental
M8	Quantitative	Multi-element analysis for 2-5 compounds of the same chemical class	Sophisticated instrumentation (LC–MS, GC–MS, ICP–MS, homemade interfaces, and homemade automatic systems	1	Simple, low cost sample preparation required	5-10	Common commercially available reagents	Preconcentration required after complicated stages	Semi-automated with common devices (e.g. HPLC autosampler)	<10 mL food/environmental
M9	Quantitative	Multi-element analysis for 2-5 compounds of the same chemical class	Simple instrumentation available in most labs	2-12	Multi-step sample preparation required	5-10	Need to be synthesized in the lab with advanced equipment or know how	Preconcentration required after complicated stages	Semi-automated with common devices (e.g. HPLC autosampler)	<10 mL food/environmental
M10	Quantitative	Multi-element analysis for 2-5 compounds of the same chemical class	Simple instrumentation	1	Miniaturized extraction sample preparation	1	Need to be synthesized in the lab with common	Preconcentration required after complicated stages	Semi-automated with common devices (e.g. HPLC autosampler)	<10 mL food/environmental

No.	Criterion 1	Criterion 2	Criterion 3	Criterion 4	Criterion 5	Criterion 6	Criterion 7	Criterion 8	Criterion 9	Criterion 10
	Type of analysis	Multi- or single-element analysis	Analytical technique	Simultaneous sample preparation	Sample preparation	Samples per h	Reagents and materials	Preconcentration	Degree of automation	Amount of sample
			available in most labs				instrumentation and in simple way			
M11	Quantitative	Multi-element analysis for 2-5 compounds of the same chemical class	Simple instrumentation available in most labs	2-12	Multi-step sample preparation required	2-4	Commercially available reagents not commonly used in QC laboratories	Preconcentration required after complicated stages	Semi-automated with common devices (e.g. HPLC autosampler)	<10 mL food/environmental
M12	Quantitative	Multi-element analysis for 6-15 compounds of the same chemical class	Simple instrumentation available in most labs	13-95	Multi-step sample preparation required	2-4	Need to be synthesized in the lab with advanced equipment or know how	Preconcentration required after complicated stages	Semi-automated with common devices (e.g. HPLC autosampler)	<10 mL food/environmental
M13	Quantitative	Multi-element analysis for 2-5 compounds of the same chemical class	Simple instrumentation available in most labs	2-12	Miniaturized extraction sample preparation	2-4	Need to be synthesized in the lab with common instrumentation and in simple way	Preconcentration required after complicated stages	Semi-automated with common devices (e.g. HPLC autosampler)	<10 mL food/environmental
M14	Quantitative	Multi-element analysis for 6-15 compounds of the same chemical class	Simple instrumentation available in most labs	2-12	Multi-step sample preparation required	2-4	Commercially available reagents not commonly used in QC laboratories	Preconcentration required after complicated stages	Semi-automated with common devices (e.g. HPLC autosampler)	<10 mL food/environmental
M15	Quantitative	Multi-element analysis for 6-15 compounds of the same chemical class	Simple instrumentation available in most labs	1	Simple, low cost sample preparation required	>10	Common commercially available reagents	Preconcentration required after complicated stages	Semi-automated with common devices (e.g. HPLC autosampler)	<10 mL food/environmental
M16	Quantitative	Multi-element analysis for 6-15 compounds of the same chemical class	Simple instrumentation available in most labs	1	Simple, low cost sample preparation required	2-4	Common commercially available reagents	Preconcentration required after complicated stages	Semi-automated with common devices (e.g. HPLC autosampler)	<10 mL food/environmental

Table S4. Detailed criterion-level assessment of the selected methods using IMOGI.

No.	Criterion 1	Criterion 2	Criterion 3	Criterion 4	Criterion 5	Criterion 6	Criterion 7	Criterion 8	Criterion 9	Criterion 10
	To what extent is the analytical process automated, including sample preparation, instrumental analysis, and data processing?	To what extent is the analytical system miniaturized or based on micro-scale or nano-scale technology?	To what extent does the method employ advanced instrumentation for sample preparation or analysis?	To what extent does the method utilize advanced digital tools, data analytics, or artificial intelligence (AI/ML) for analysis, optimization, or interpretation?	How adaptable or scalable is the method for future AI, automation integration, or application to new analytes or matrices	To what extent does the method introduce a novel concept, principle, or mechanism?	To what extent does the method utilize novel reagents, materials, or probes?	To what extent does the method improve efficiency, throughput, or overall performance compared to existing methods?	To what extent does the method integrate knowledge from multiple scientific fields or foster interdisciplinary collaboration?	To what extent does the analytical method consider the principles of green and white analytical chemistry and incorporate recognized evaluation metrics or indexes (e.g. AGREE, AGREEprep, MoGAPI, BAGI, RAPI)?
M1	Partially automated	Partially miniaturized	Only conventional technologies (HPLC-UV)	Uses standard or routine data processing or software	Adaptable with some modifications	Routine application and applies an established method without significant change	Uses conventional materials or reagents	Moderate improvement in method performance (speed/sensitivity/throughput)	Stays within a single field with no integration	Does not consider any principles/no metrics are used
M2	Partially automated	Conventional large-scale system	Only conventional technologies (HPLC-UV)	Uses standard or routine data processing or software	Adaptable with some modifications	Routine application and applies an established method without significant change	Uses conventional materials or reagents	Comparable or worse performance	Stays within a single field with no integration	Does not consider any principles/no metrics are used
M3	Partially automated	Conventional large-scale system	Only conventional technologies (HPLC-UV)	Uses standard or routine data processing or software	Adaptable with some modifications	Entirely new approach/principle/technology	Uses completely new reagents/nanomaterials/selective probes	Moderate improvement in method performance (speed/sensitivity/throughput)	Stays within a single field with no integration	Does not consider any principles/no metrics are used
M4	Partially automated	Partially miniaturized	Only conventional technologies (HPLC-UV)	Uses standard or routine data processing or software	Adaptable with some modifications	Routine application and applies an established method without significant change	Uses completely new reagents/nanomaterials/selective probes	Moderate improvement in method performance (speed/sensitivity/throughput)	Stays within a single field with no integration	Considers all principles/many metrics are used
M5	Partially automated	Partially miniaturized	Only conventional technologies (HPLC-UV)	Uses standard or routine data processing or software	Adaptable with some modifications	Modifies an existing method in a meaningful way or extends method to new contexts	Uses completely new reagents/nanomaterials/selective probes	Comparable or worse performance	Stays within a single field with no integration	Does not consider any principles/no metrics are used
M6	Partially automated	Conventional large-scale system	Only conventional technologies (HPLC-UV)	Uses standard or routine data processing or software	Adaptable with some modifications	Routine application and applies an established method without significant change	Uses conventional materials or reagents	Comparable or worse performance	Stays within a single field with no integration	Does not consider any principles/no metrics are used
M7	Partially automated	Partially miniaturized	Partially advanced technologies (e.g. UHPLC)	Uses standard or routine data processing or software	Adaptable with some modifications	Modifies an existing method in a meaningful way or extends method to new contexts	Uses completely new reagents/nanomaterials/selective probes	Moderate improvement in method performance (speed/sensitivity/throughput)	Stays within a single field with no integration	Considers all principles/many metrics are used
M8	Partially automated	Conventional large-scale system	Only conventional technologies (HPLC-UV)	Uses standard or routine data processing or software	Adaptable with some modifications	Modifies an existing method in a meaningful way or extends method to new contexts	Uses conventional materials or reagents	Moderate improvement in method performance (speed/sensitivity/throughput)	Stays within a single field with no integration	Considers all principles/many metrics are used
M9	Partially automated	Conventional large-scale system	Only conventional technologies (HPLC-UV)	Uses standard or routine data processing or software	Adaptable with some modifications	Modifies an existing method in a meaningful way or extends method to new contexts	Uses completely new reagents/nanomaterials/selective probes	Moderate improvement in method performance	Stays within a single field with no integration	Does not consider any principles/no metrics are used

No.	Criterion 1	Criterion 2	Criterion 3	Criterion 4	Criterion 5	Criterion 6	Criterion 7	Criterion 8	Criterion 9	Criterion 10
								(speed/sensitivity/throughput)		
M10	Partially automated	Partially miniaturized	Only conventional technologies (HPLC-UV)	Uses standard or routine data processing or software	Adaptable with some modifications	Modifies an existing method in a meaningful way or extends method to new contexts	Uses completely new reagents/nanomaterials/selective probes	Moderate improvement in method performance (speed/sensitivity/throughput)	Stays within a single field with no integration	Does not consider any principles/no metrics are used
M11	Partially automated	Conventional large-scale system	Only conventional technologies (HPLC-UV)	Uses standard or routine data processing or software	Adaptable with some modifications	Routine application and applies an established method without significant change	Uses conventional materials or reagents	Moderate improvement in method performance (speed/sensitivity/throughput)	Stays within a single field with no integration	Does not consider any principles/no metrics are used
M12	Partially automated	Conventional large-scale system	Only conventional technologies (HPLC-UV)	Uses standard or routine data processing or software	Adaptable with some modifications	Modifies an existing method in a meaningful way or extends method to new contexts	Uses completely new reagents/nanomaterials/selective probes	Substantially faster/more sensitive/higher throughput than conventional methods	Stays within a single field with no integration	Considers all principles/many metrics are used
M13	Partially automated	Partially miniaturized	Only conventional technologies (HPLC-UV)	Uses standard or routine data processing or software	Adaptable with some modifications	Modifies an existing method in a meaningful way or extends method to new contexts	Uses completely new reagents/nanomaterials/selective probes	Moderate improvement in method performance (speed/sensitivity/throughput)	Stays within a single field with no integration	Considers all principles/many metrics are used
M14	Partially automated	Conventional large-scale system	Partially advanced technologies (e.g. UHPLC)	Uses standard or routine data processing or software	Adaptable with some modifications	Routine application and applies an established method without significant change	Uses conventional materials or reagents	Comparable or worse performance	Stays within a single field with no integration	Does not consider any principles/no metrics are used
M15	Partially automated	Fully miniaturized	Only conventional technologies (HPLC-UV)	Uses standard or routine data processing or software	Adaptable with some modifications	Modifies an existing method in a meaningful way or extends method to new contexts	Uses conventional materials or reagents	Moderate improvement in method performance (speed/sensitivity/throughput)	Stays within a single field with no integration	Does not consider any principles/no metrics are used
M16	Partially automated	Conventional large-scale system	Only conventional technologies (HPLC-UV)	Uses standard or routine data processing or software	Adaptable with some modifications	Routine application and applies an established method without significant change	Uses conventional materials or reagents	Moderate improvement in method performance (speed/sensitivity/throughput)	Stays within a single field with no integration	Does not consider any principles/no metrics are used

Table S5. Database-specific literature search strategies.

Database	Web of Science	Scopus
Search platform / collection	Advanced Search; Web of Science Core Collection	Advanced Document Search
Search strategy	Parabens* cosmetics* liquid chromatography*	TITLE-ABS-KEY (paraben*) AND TITLE-ABS-KEY ((cosmetic* OR "personal care product*") AND ("liquid chromatography" OR HPLC OR UHPLC OR "LC-MS" OR "LC-MS/MS"))
